# Sensing centrosome amplification: the interface between centriole duplication and autophagy

**DOI:** 10.1038/s41467-026-74702-9

**Published:** 2026-06-21

**Authors:** Paula A. Coelho, Agnieszka Fatalska, Marco Geymonat, Ramona Lattao, David M. Glover

**Affiliations:** 1 Division of Biology and Biological Engineering, California Institute of Technology, Pasadena, CA USA; 2 Department of Genetics, University of Cambridge, Cambridge, UK; 3 Present Address: Institute of Biochemistry and Cell Biology (IBBC), National Research Council (CNR), Monterotondo (Rome), Italy

**Keywords:** Tumour-suppressor proteins, Macroautophagy, Centrosome

## Abstract

Multipolar mitotic spindles with extra centrosomes, first observed in cancer cells in the late nineteenth century, remain poorly understood. Here, to address how cells overcome proliferation arrest imposed by centrosome amplification, we describe a genome-wide screen revealing that downregulation of the Wnt, Hippo, Tpr53, PIDDosome, ciliary biogenesis, or autophagy pathways enables proliferation of mouse embryonic stem cells having PLK4-mediated centrosome amplification. We select the tumor suppressor, Guanine-nucleotide Activating Protein ARHGAP15, for further study as its depletion activates autophagy, overcomes centrosome amplification, and enables embryonic fibroblast proliferation. Reduction of centrosomes following ARHGAP15 depletion requires autophagy protein, ATG16L1, which associates with ARHGAP15 when the autophagy pathway is inactive. ARHGAP15 is opposed by Guanine-nucleotide Exchange Factor ARHGEF2, which is activated by the centriolar protein CEP170 to generate RAC1-GTP and promote autophagy. Together our findings add extra dimensions to the roles of RAC1 in cytoskeletal regulation and ARHGAP15 as a potential tumor suppressor.

## Abstract (web summary)

A genome wide screen reveals down-regulating the Wnt, Hippo, Tpr53, PIDDosome, ciliary biogenesis, or autophagy pathways overcomes inhibitory effects of PLK4-mediated centrosome amplification on cell proliferation. Depletion of tumor suppressor, ARHGAP15, activates autophagy to reduce centrosomes. ARHGAP15 is opposed by ARHGEF2, which is activated by centriolar protein CEP170 to generate RAC1-GTP and promote autophagy.

## Introduction

Centrosomes, discovered in the late 19^th^ century^1,2^, organize microtubules at the mitotic spindle poles. Proliferating cells must, therefore, coordinate centrosome and cell duplication to ensure each cell has just two centrosomes. Such coordination is lost in cancer cells, where centrosome amplification^3^ characterizes chromosome instability^4,5^ and invasiveness^6,7^. Supernumerary centrosomes in pre-neoplastic lesions indicate greater risk for malignant transformation^8^. However, the relationship of centrosome amplification to tumorigenesis remains poorly understood despite having been recognized for over a century^9,10^. Based on 19th century observations of tumor cells and his demonstration of aneuploidy in sea urchin eggs containing multiple centrosomes, Boveri proposed that centrosome amplification led to aneuploidy, a root cause of cancer^11^. We now know that perturbation of myriad signalling pathways contributes to malignant transformation and it is not unreasonable that these interface with the centrosome duplication cycle through an equally diverse set of molecular pathways.

Centrosomes can be amplified in transgenic mice by elevating Polo-like-kinase-4 (PLK4) and accelerate tumour incidence or hyperplasia caused by loss of the Trp53 tumor suppressor^12,13^ or by a single truncated allele of the adenomatous-polyposis-coli tumor suppressor, ApcMin/+^14^. This accords with clinical data and suggests extra-centrosomes are not a cancer driver per se but that cancer cells can overcome the survival block imposed by centrosome amplification in poorly understood Tp53- or Apc-mediated processes. The extra centrosomes resulting from cytokinesis failure also block cell proliferation by signalling through LATS2 protein kinase leading to the stabilization of p53^15^. Moreover, extra centrosomes trigger the activation of the PIDDosome^16^ through the centriolar distal appendage protein ANKRD26^17,18^ leading to p53 stabilization, and p21-dependent cell cycle arrest. Although one cannot exclude toxic effects due to *Plk4* over-expression, the consequential inhibition of cell proliferation or apoptosis has been directly linked to centrosome amplification^6^, whereas non-centrosomal PLK4 functions reported to date are predominantly pro-survival or pro-invasive^19^.

Whether these two pathways acting through p53 represent the entirety of the interface between the centrosome-duplication and cell-division cycles, and how centrosomes might interact with the variety of signalling pathways that mediate cancer cell survival, is far from clear. In part, this is because cancer subtypes have distinct genetic abnormalities and rely on distinct signalling pathways for survival as reflected in tissue specificity or response to anticancer agents^20,21^. The absence of tight regulation of centrosome numbers due to additional mutations promoting cancer cell survival is therefore perhaps not surprising. Here, we wished to identify pathways that, upon their inactivation, would permit the proliferation and survival of cells with extra-centrosomes and to this end carried out a genome wide CRISPR/Cas9-mediated screen. We carried out this screen in pluripotent embryonic stem cells (ESCs) because they display some common properties with pre-cancer cells: they do not undergo quiescence or senescence; they are refractory to contact inhibition and less dependent on extracellular proliferation signals; they have low expression of cyclin-dependent-kinase inhibitor p21; they do not show a p53 response to DNA damage; they have inactive retinoblastoma protein resulting in a short G1; and they show impaired control of several cell cycle checkpoints^22^. Yet, proliferation of ESCs is prevented by supernumerary centrosomes, resulting from elevated *Plk4* expression. In addition to the Hippo/Lats^15,23,24^ and Piddosome^16^ pathways acting through Tpr53^25^, our screen identified the Wnt signalling^19,26,27^, Proteasome-dependent degradation^28–31^, and autophagy pathways as mechanisms that signal arrest of cell proliferation in the presence of extra centrosomes.

Here we focus upon the ability of extra centrosomes to signal autophagy. During autophagy, a double membrane (the phagophore) elongates from the endoplasmic reticulum (ER) and to form autophagosomes, which either fuse with late endosomes to form amphisomes and later with lysosomes or directly with lysosomes. Substrates to be degraded are recognized by the autophagy-related protein ATG16L1, which also contributes to phagosome elongation^32^. We show that the cell proliferation block induced by centrosome amplification can be overcome by loss of ARHGAP15, a RAC1-specific GTPase–Activating Protein (GAP), previously shown to be downregulated in several cancer cell types^33–37^. We show ARHGAP15 negatively regulates ATG16L1’s role in autophagy which is triggered by ATG1/ULK1 kinase. We find the centriole’s subdistal appendage protein CEP170 complexes with ARHGEF2, a Rho/Rac Guanine-nucleotide Exchange Factor 2 (GEF) that opposes ARHGAP15 to enhance autophagy and reduce centrosome overduplication.

## Results

### Genes blocking proliferation of embryonic stem cells exhibiting centrosome amplification

**Fig. 1:**
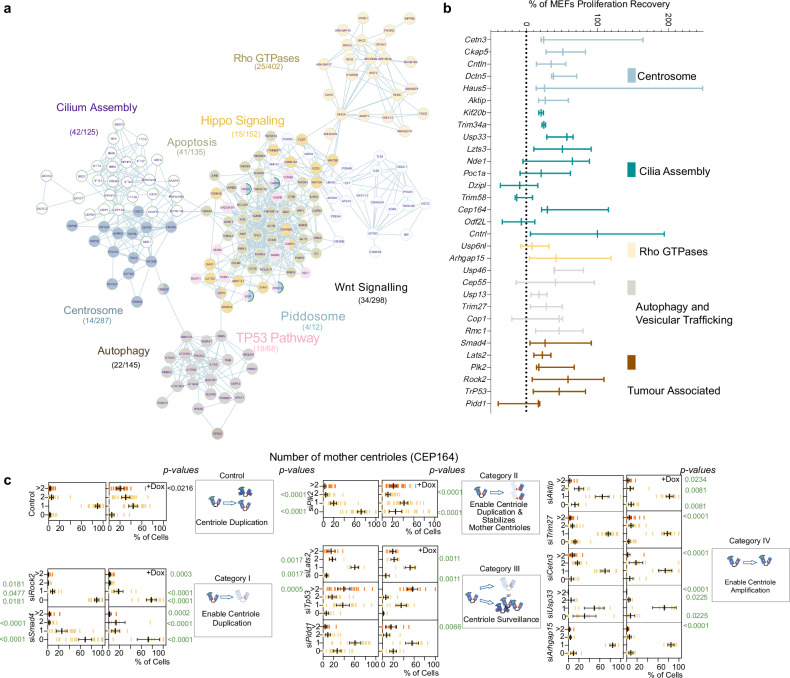
A genome-wide CRISPR–Cas9 screen identifies mouse genes that suppress proliferation in the presence of extra centrosomes. **a** Schematic representation of gene networks clustered within enriched pathways identified by overrepresentation analysis and functional class scoring (FCS). Genes were grouped using STRING analysis to identify protein–protein interaction subnetworks (PINs). **b** Thirty-two genes identified in the CRISPR–Cas9 screen in embryonic stem cells (ESCs) were selected for validation by siRNA-mediated depletion in mouse embryonic fibroblasts (MEFs) derived from *Tet*^*ON*^–*Plk4* transgenic mice. Proliferation assays were performed in the absence or presence of doxycycline (−Dox/+Dox) following gene knockdown. Data are derived from *n* = 3 independent biological replicates and are plotted showing the minimum and maximum values. Genes are colour-coded according to KEGG classification: centrosome (light blue), cilia assembly (dark blue), autophagy and vesicle trafficking (grey), Rho GTPases (yellow), and tumour-associated pathways (brown). **c** Centrosome surveillance responses following siRNA knockdown in Tet^ON^–*Plk4* MEFs. The percentage of cells in each mother centriole category is shown: 0 or 1–2 mother centrioles (yellow) and >2 mother centrioles (orange). Data are from independent biological replicates: n = 11 for Control, Control + Dox, si*Smad4*, si*Pidd1* + Dox, si*Cetn3* +Dox, si*Trim27* + Dox, si*Usp33*, si*Usp33* + Dox; n = 10 for si*Trp53*, si*Trp53* + Dox, si*Plk2*, si*Plk2* + Dox, si*Arhgap15*, si*Trim27*; and *n* = 9 for si*Smad4* + Dox, si*Lats2*, si*Lats2* + Dox, si*Rock2*, si*Rock2* + Dox, si*Aktip*, si*Aktip* + Dox, si*Pidd1*, si*Arhgap15* + Dox and si*Cetn3*. Error bars indicate mean ± 95% confidence interval (CI). Statistical analysis was performed using the Kruskal–Wallis test followed by Dunn’s multiple comparisons test. The *p*-values associated with statistical significance are indicated in black (control −Dox versus control +Dox) and green (control versus the corresponding samples under equivalent *Plk4*^OE^ conditions). Cellular responses were grouped into four phenotypic categories: category I, genes required for centriole duplication; category II, genes required for maintenance of mother centrioles; category III, genes supporting proliferation in the presence of extra centrosomes; and category IV, genes required for centriole amplification, defined by failure to amplify centrosomes despite *Plk4* overexpression. Source data and full statistical analyses are provided in Supplementary Data 2.

To identify genes that when mutated can overcome the proliferation block imposed by extra centrosomes, we performed a genome-wide CRISPR-Cas9 knockout screen on mouse (m)ESCs in which PLK4 can be elevated by doxycycline to drive centrosome duplication (hereafter, *Plk4*^*OE*^ *ESC*s; Fig. 1a; Supplementary Fig. 1a; Supplementary Fig. 2a, b)^13^. The *Plk4*^*OE*^ *ESC* line was first transduced with lentivirus carrying *Cas9-EGFP* (*Plk4*^*OE*^*, Cas9 mESC*) and subsequently with a lentivirus library of single-guide RNAs (sgRNAs), in which each mouse gene was represented by approximately 10 unique sgRNAs^38^ (~180,000 sgRNAs total), and cultured in the presence or absence (control) of doxycycline for 9 further days following the 4^th^ day post transduction (Supplementary Fig. 1a). Centrosome amplification and maintenance of euploidy in the *Plk4-OE ESC*s was observed during this period of *Plk4* overexpression (Supplementary Fig. 1a–b; Supplementary Fig. 2a–d) This treatment resulted in quiescence or cell death of control *Plk4-OE, Cas9 ESCs* expressing elevated *Plk4* and not transduced with the sgRNA library (Supplementary Fig. 1a). Following approximately 9 population doublings of *Plk4-OE, Cas9 mESC*s induced to overexpress PLK4 and transduced with the sgRNA library, genomic DNA was prepared and subjected to PCR and deep sequencing from primers complementary to sequences flanking the sgRNA targets (Supplementary Fig. 1a; Supplementary Data 1 & 12; Methods). We selected genes of interest that, following knockout, were represented by 1000 reads or more for at least two sgRNAs.

We searched the Gene Ontology (GO) or Kyoto Encyclopaedia of Genes and Genomes (KEGG) databases to group gene products identified in the screen into interacting networks. In addition to the Hippo^15,23,39,40^ and Piddosome^16^ pathways, previously shown to activate Tpr53^25^ to block the proliferation of cells with amplified centrosomes, and the Wnt pathway, which has several effects upon centrosome function^19,41,42^ (Fig. 1a, Supplementary Data 1), we found enrichment of pathways not previously implicated in centrosome surveillance including small GTPases and their regulatory proteins, and proteins functioning in autophagy, vesicle trafficking, cilium assembly, and in the late stages of centrosome assembly (Fig. 1a, Supplementary Data 1).

To validate these hits and establish their function in cells having conventional cell cycles with more functional checkpoints, we used RNAi to deplete their gene products in mouse embryonic fibroblasts established from transgenic mice homozygous for the *Tet*^*ON*^ *Plk4* transgene at *rosa26*^13^, (*Tet*^*ON*^*-Plk4* MEFs). Centrosomal phenotypes were characterized by assessing the effects of gene depletion under basal conditions and following *Plk4* overexpression, with comparisons to the corresponding controls in the presence or absence of centrosome amplification (+ or – Dox (Doxycycline)). Following siRNA-mediated knockdown, immunofluorescence analysis revealed altered centrosome numbers for a subset of genes. Two centriolar markers were employed: CEP164, a distal appendage protein that labels mother centrioles and is typically observed as a single punctate signal in interphase or two signals in mitotic cells, with higher numbers indicating centrosome amplification; and CEP152, a pan-centriolar marker that labels all centrioles and is normally detected as one or two pairs in cycling cells but greater than two pairs in cells with amplified centrosomes (Supplementary Fig. 2e-f).

We selected 32 such genes for siRNA-mediated knockdown (Supplementary Fig. 2g-j) and monitored proliferation recovery in *Tet*^*ON*^*-Plk4* MEFs with extra centrosomes following *Plk4* overexpression compared to control cells (Fig. 1b). Knockdown of *Pidd1*, *Trp53*, and *Lats2* all enabled recovery of cell proliferation in *Plk4* ^*OE*^ cells; p53 depletion gave the strongest response and PIDD1 depletion, the weakest. In *Tet*^*ON*^*-Plk4* MEFs, we found a similar range of recovery of proliferation following depletion of proteins exemplifying small GTPase regulation, autophagy, cilium assembly, and centrosome assembly categories confirming their roles in preventing cell proliferation following centrosome amplification (Fig. 1b).

We next determined the consequences of knockdown of these protein categories upon centriole duplication with and without elevating PLK4. To this end we stained untreated or doxycycline-treated *Tet*^*ON*^*-Plk4* MEFs to reveal CEP164 and classified cells as having no mother centrioles; one or two mothers as in wild-type G1 or G2/M cells, respectively; or more than 2 mothers typifying centrosome amplification (Fig. 1c; Supplementary Fig. 1c, Supplementary Fig. 2f). Staining for CEP152 revealed all mother centrioles and daughter centrioles following centriole to centrosome conversion (Supplementary Fig. 1d, e).

We grouped the cellular responses to downregulation of the selected genes into four categories: Category I proteins, exemplified by SMAD4 and ROCK2, appear to be required for centriole duplication. Their depletion increased cell numbers lacking centrioles and elevated PLK4 could not rescue this phenotype (Fig. 1c; Supplementary Fig. 1c–e; Supplementary Data 2). This accords with previous findings that ROCK2 kinase, activated by Rho GTPases to regulate the actin cytoskeleton^43–46^, is partnered with Nucleophosmin at centrosomes^43,47–50^ and required to enable centrosome duplication. A similar phenotype was seen following depletion of the SMAD4, component of the nuclear SMAD2/SMAD3-SMAD4 complex and mediator of signal transduction by TGF-β (transforming growth factor)^51^ but, to our knowledge, not previously implicated in centrosome duplication.

Category II genes, best exemplified by *Plk2*, are also required for centriole duplication, but their knockdown can be rescued by *Plk4* ^*OE*^. PLK2 depletion also decreases the percentage of cells having mother centrioles and is likely to have multiple roles in centriole duplication. It indirectly activates ROCK2^52,53^ and can also phosphorylate S595 of CPAP to facilitate centriole elongation^52^. PLK4 can also phosphorylate this residue on CPAP and can perhaps also phosphorylate other PLK2 substrates, accounting for the ability of elevated PLK4 to rescue the PLK2 knockdown and drive centriole duplication (Fig. 1c; Supplementary Fig. 1c–e; Supplementary Data 2).

Category III is represented by proteins previously shown to have a role in centrosome surveillance. A common feature following the depletion of three such proteins, LATS2, P53, or PIDD1, is generation of amplified CEP152-marked centrioles in the absence of *Plk4* ^OE^ (Supplementary Fig. 1d, e). The response to P53 knockdown per se accords with previous reports of P53 involvement in centrosome integrity in non-transformed cells^54^ and P53’s role in centrosome surveillance^55^. Ganem and colleagues have shown extra centrosomes alter small G protein signalling and activate LATS2 kinase, which in turn stabilizes p53^15^. PIDD1 is part of the PIDDosome complex, whose activation by supernumerary centrosomes results in cleavage of p53’s key inhibitor, MDM2, to stabilize p53 and activate CASP2 to trigger apoptosis^16–18,56^.

Category IV proteins enable centriole amplification; we exemplify five such gene products, AKTIP, TRIM27, ARHGAP15, USP33 and CETN3, the deletion of any of which suppresses centrosome amplification by PLK4 and promotes cell proliferation. In addition, AKTIP (AKT Interacting Protein) depletion also reduces the numbers of CEP152 positive, presumptive daughter centrioles, following *Plk4* ^OE^. AKTIP localizes at centrosomes^57^ and enhances phosphorylation of AKT protein kinase that regulates cell proliferation and the anti-apoptotic response^58^. Inhibition of AKT is also reported to reduce recruitment of γ-tubulin and PLK1 to mitotic centrosomes^59^. Two other of these five proteins participate directly in centriole assembly: USP33 deubiquitinates CP110 in S and G2 phase and USP33 depletion destabilizes CP110 to inhibit centriole duplication^60^. Calcium-binding CETN3 is a mammalian centriolar protein whose role in centriole duplication was hypothesized by the observation that HsCen3p blocks yeast Spindle Pole Body (SPB) and frog centrosome duplication by competition with its counterparts^61^. TRIM27 is a multifunctional ubiquitin protein ligase. It interacts with the IκB kinase (IKK) family and with TBK1, which inhibits NF-κB-dependent and IRF3-dependent transcriptional activation^62^. More recently, TRIM27 was found to polyubiquitinate and so oppose autophagy promoting ULK1 kinase^63^.

**Fig. 2:**
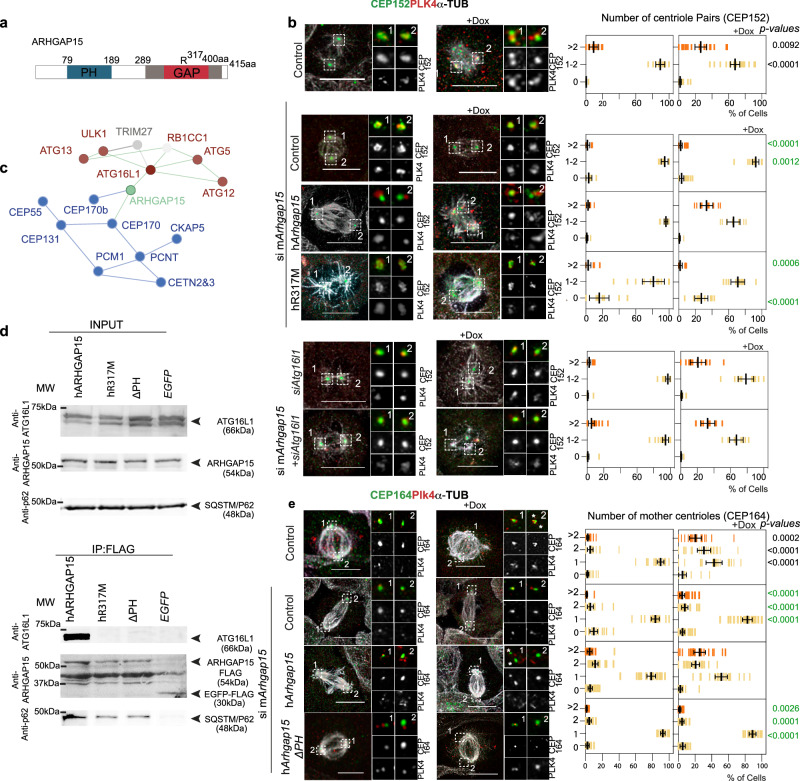
ARHGAP15 regulates centrosome number through ATG16L1. **a** ARHGAP15 is a GTPase-activating protein containing a canonical GAP domain with an arginine finger, in which R317 is essential for GTP hydrolysis. **b** The indicated cell lines were treated with siRNAs and with or without doxycycline (−/+Dox) to induce PLK4 overexpression, then stained for PLK4 (red), CEP152 (green) and α-tubulin (white). Scale bar, 10 µm. Graphs show the percentage of cells with the indicated numbers of CEP152-stained centrioles; each bar represents one randomly selected field, as described in the Methods. Data are from independent biological replicates: *n* = 27 for Control + Dox; *n *= 20 for si*mArhgap15* + Dox and si*mArhgap15* + si*Atg16l1*; *n* = 16 for si*Atg16l1*; *n* = 13 for hARHGAP15 + si*mArhgap15* and R317M + si*mArhgap15*; *n* = 12 for Control, si*mArhgap15* and si*Atg16l1* + Dox; *n* = 10 for hARHGAP15 + si*mArhgap15* + Dox and R317M + si*mArhgap15* + Dox; and *n *= 9 for si*mArhgap15* + si*Atg16l1* + Dox. Black error bars indicate mean ± 95% confidence interval (CI). Statistical analysis was performed using the Kruskal–Wallis test followed by Dunn’s multiple comparisons test. P values are shown in black for control −Dox versus control +Dox, and in green for control versus samples under equivalent *Plk4*^OE^ conditions. Centriole number was quantified for each category, and all values are included in Supplementary Data 3. The specificity of *Arhgap15* siRNA was assessed in *Plk4*^OE^ MEFs stably expressing human wild-type ARHGAP15 or the catalytically inactive R317M mutant. *Plk4*^OE^ MEFs were also subjected to siRNA-mediated depletion of endogenous mouse *Arhgap15* or *Atg16l1*, alone or in combination, as indicated. **c** Schematic representation of the ARHGAP15 subnetwork identified by pull-down and mass spectrometry. Proteins proximal to ARHGAP15 are classified according to roles in autophagy (brown) or centrosomal association (blue). The corresponding mass spectrometry datasets are shown in Supplementary Data 4. **d** Extracts of *Plk4*^OE^ MEFs expressing FLAG-tagged wild-type ARHGAP15, the GAP-deficient R317M mutant, the ΔPH mutant lacking the pleckstrin homology (PH) domain, or EGFP–FLAG control were analysed in total lysates (input) and anti-FLAG immunoprecipitates by western blotting. Protein samples were loaded in parallel on polyacrylamide gels and, after transfer, the membranes were incubated separately with the indicated antibodies. Experiments were repeated independently three times with reproducible results (*n* = 3), and raw data are provided in Supplementary Data 3.Experiments were performed independently three times (*n* = 3), and raw data are provided in Supplementary Data 3. **e** Representative images of MEFs expressing wild-type hARHGAP15 or hARHGAP15_ΔPH after depletion of endogenous mouse *Arhgap15*. Cells were stained for PLK4 (red), CEP164 (green) and α-tubulin (white). Scale bar, 10 µm. Graphs show the percentage of cells in each mother centriole category per cell. Data are from independent biological replicates: *n *= 28 for Control; *n* = 24 for hARHGAP15 + si*mArhgap15*; *n* = 23 for hARHGAP15 + si*mArhgap15* + Dox; *n* = 21 for Control + Dox and si*mArhgap15* + Dox; *n *= 12 for hARHGAP15_ΔPH + si*mArhgap15* and hARHGAP15_ΔPH + si*mArhgap15* + Dox; and *n *= 11 for si*mArhgap15*. Black error bars indicate mean ± 95% CI. Statistical analysis was performed as in (**b**). All quantifications and statistics are included in Supplementary Data 3.

Of the above Category IV proteins, our attention was caught by ARHGAP15, a RAC1 specific GAP domain containing protein with an N-terminal Pleckstrin Homology (PH) domain^64^ (Fig. 2a). Depletion of ARHGAP15 rescued the proliferation of *Plk4*-overexpressing MEFs and restored the PLK4-promoted increase in centrosomes to control levels; whereas 28.25 ± 3.115 (sem)% of doxycycline-treated *Plk4* ^*OE*^ cells had greater than 2 centrosomes, just 2.32 ± 0.94 (sem)% of such cells had more than 2 centrosomes after ARHGAP15 depletion (Fig. 2b, Supplementary Data 3).

### ARHGAP15 is a negative regulator of autophagy

**Fig. 3:**
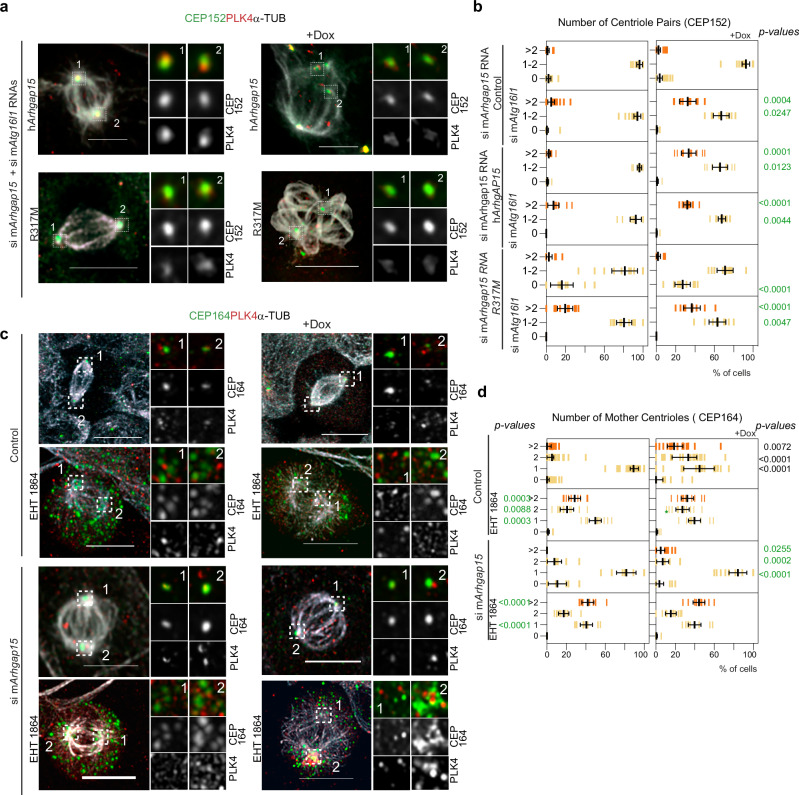
ARHGAP15 is a negative regulator of autophagy, and RAC1 regulates centrosome amplification. **a** *Tet*^*ON*^*-Plk4* MEFs stably expressing hARHGAP15 or the hARHGAP15 R317M mutant were stained to visualize centrosomes following siRNA-mediated depletion of endogenous mouse *Arhgap15* and *Atg16l1*, in the absence or presence of doxycycline (+Dox) to induce PLK4 expression. Representative immunofluorescence images are shown. Scale bar, 10 µm. **b** Quantification of the percentage of cells with 0, 1–2, or >2 centriole pairs per cell. Data are from independent biological replicates:* n* = 9 for si*Arhgap15* + si*Atg16l1* + Dox; *n* = 10 for hARHGAP15 + si*Arhgap15* + Dox, R317M + si*Arhgap15* + Dox and R317M + si*Arhgap15* + si*Atg16l1*; *n* = 11 for R317M + si*Arhgap15* + si*Atg16l1* + Dox; *n* = 12 for Control, si*Atg16l1* + Dox, si*Arhgap15*, si*Arhgap15* + Dox and hARHGAP15 + si*Arhgap15* + si*Atg16l1*; *n* = 13 for hARHGAP15 + si*Arhgap15*, R317M + si*Arhgap15* and hARHGAP15 + si*Arhgap15* + si*Atg16l1* + Dox; *n* = 16 for si*Atg16l1*; *n* = 20 for si*Arhgap15* + si*Atg16l1*; and *n* = 27 for Control + Dox. Black error bars indicate mean ± 95% confidence interval (CI). Statistical analysis was performed using the Kruskal–Wallis test followed by Dunn’s multiple comparisons test. *P* values are indicated in the figure: black, control −Dox versus control +Dox; green, control versus equivalent *Plk4*^OE^ conditions. Raw data and statistical analyses are included in Supplementary Data 5. **c** Representative mitotic figures of control *Tet*^*ON*^*-Plk4* MEFs and *Tet*^*ON*^*-Plk4* MEFs depleted of ARHGAP15 by siRNA, cultured in the absence or presence of doxycycline. RAC1 activity was inhibited with EHT 1864 in control MEFs or following *Arhgap15* siRNA, with or without doxycycline. Immunostaining reveals PLK4 (red), the distal appendage protein CEP164 (green), and α-tubulin (white, spindle). CEP164 marks mother centrioles throughout the cell cycle, allowing centrosome amplification to be scored as cells containing >2 mother centrioles. Scale bar, 10 µm. Insets show individual centrosomes at higher magnification. **d** Quantification of the percentage of cells with 0, 1–2, or >2 mother centrioles per cell, with individual field values shown as yellow or orange bars. Data are from independent biological replicates: *n* = 38 for Control and *n* = 21 for Control + Dox; *n *= 12 for Control + RAC1 inhibitor (EHT 1864) and Control + RAC1 inhibitor (EHT 1864) + Dox; *n* = 11 for si*Arhgap15*, si*Arhgap15* + RAC1 inhibitor (EHT 1864), and si*Arhgap15* + RAC1 inhibitor (EHT 1864) + Dox; and *n* = 14 for si*Arhgap15* + Dox. Black error bars indicate mean ± 95% CI. Statistical analysis was performed using the Kruskal–Wallis test followed by Dunn’s multiple comparisons test. *P* values are indicated in the figure: black, control −Dox versus control +Dox; green, control versus equivalent *Plk4*^OE^ conditions. Raw data and statistical analyses are included in Supplementary Data 5.

We first asked whether ARHGAP15’s catalytic activity was required to regulate centrosome numbers. To this end, we established one line of *Tet*^*ON*^*-Plk4* MEFs stably expressing human (h*)*ARHGAP15, and a second, expressing (h)ARHGAP15 carrying an R317M mutation inactivating the GTPase domain’s catalytic arginine finger, both being resistant to siRNAs targeting the endogenous mouse protein. Restoration of amplification of CEP152-marked centrioles was seen in cells following depletion of endogenous ARHGAP15 from doxycycline-treated *Tet*^*ON*^*-Plk4* MEFs expressing wild-type hARHGAP15 protein demonstrating the specificity of the siRNAs knockdown. However, only 1.678% ± 1.124 (sem)% of doxycycline-treated *Tet*^*ON*^*-Plk4* MEFs expressing R317M mutant hARHGAP15 showed centriole amplification indicating a functional requirement for GTPase activity (Fig. 3a, b; Supplementary Data 5). As ARHGAP15 inactivation would elevate RAC1-GTP, these observations suggest RAC1-GTP activity is crucial in reducing supernumerary centrosomes and the subsequent cellular response.

To gain greater mechanistic understanding, we searched for ARHGAP15’s molecular partners by pulling-down the tagged protein (Fig. 2c; Supplementary Data 4). This identified members of the autophagy pathway essential for substrate recognition and autophagosome biogenesis, most prominent of which was ATG16L1, which binds the ubiquitin-like folds of ATG5 and shows membrane-binding activity for LC3B/ATG8 lipidation^65^. Several centrosomal proteins also coprecipitated with ARHGAP15, the major one being the subdistal appendage protein, CEP170. Transfection of *Tet*^*ON*^*-Plk4* MEFs with FLAG tagged ARHGAP15 followed by anti-FLAG purification confirmed ARHGAP15’s interaction with ATG16L1 (Fig. 2d). However, in cells transfected with either the R317M mutant (deficient in GAP activity) or the ΔPH mutant lacking the Pleckstrin Homology (PH) domain, ATG16L1 was not detected in the pulldown. These results demonstrate that the ARHGAP15–ATG16L1 interaction requires both the GAP activity and the PH domain of ARHGAP15, the latter being critical for binding regulatory proteins and influencing subcellular localization. We also detected p62/SQSTM1 in the pulldown suggesting that this adaptor protein may potentially connect ARHGAP15 to selective autophagy pathways (Fig. 2d). In agreement with these finding the *Tet*^*ON*^*-Plk4* MEFs expressing hARHGAP15 ΔPH mutant did not show centriole amplification indicating a functional requirement for the PH activity (Fig. 2e).

RNAi-mediated depletion of ATG16L1 revealed that 20.75% ± 4.20 (sem)% of doxycycline-treated *Tet*^*ON*^*-Plk4* MEFs had greater than two centrosomes (Fig. 2b, Supplementary Data 3). We then tested the epistatic relationship between ARHGAP15 and ATG16L1 by simultaneously depleting both gene products. This phenocopied the ATG16L1 knockdown suggesting that ATG16L1 is required for depletion of ARHGAP15 to overcome centrosome duplication induced by elevated PLK4. Thus, ARHGAP15 appears to act as a negative regulator of an autophagic pathway utilizing ATG16L1 to eliminate supernumerary centrosomes. Moreover, expression of the catalytically dead hARHGAP15 R317M resulted in a substantial fraction of cells (16.08% ± 5.37(sem)%) lacking detectable centrosomes. This phenotype was reverted after ATG16L1 knockdown (Fig. 3a, b and Supplementary Data 5), indicating that loss of centrosomes is dependent on autophagy. These findings support a role of ARHGAP15 in regulating centrosome number through ATG16L1. This unusually strong centrosome-loss phenotype of R317M, even in the presence of elevated PLK4, suggests that hyperactivation of the RAC1–ATG16L1–dependent autophagy can totally override PLK4-driven centriole biogenesis, pointing to additional layers of ARHGAP15 regulation and centrosome homeostasis that remain to be elucidated. Together, these findings suggest that supernumerary centrosomes can be eliminated through autophagy and that ARHGAP15 regulates this process through active RAC1–GTP. To directly test the involvement of RAC1, we inhibited its activity using EHT 1864, a selective RAC1 inhibitor that prevents guanine nucleotide binding and thereby blocks RAC1 activation^66^. Treatment with EHT 1864 led to a significant increase in centrosome amplification (Fig. 3c, d; Supplementary Data 5), independently of Arhgap15 levels. These results indicate that RAC1, the only specific interactor of Arhgap15 identified to date, regulates centrosome amplification through autophagy, and establish RAC1 as a key mediator of the elimination of supernumerary centrosomes by autophagy.

### Autophagic flux is required to regulate centrosome numbers

**Fig. 4:**
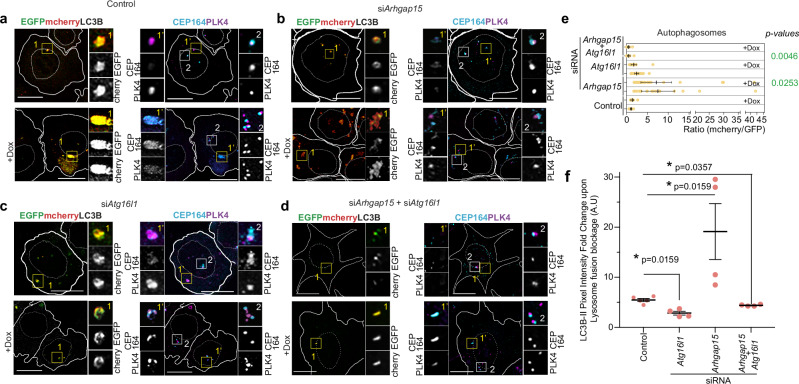
ARHGAP15 is a negative regulator of autophagy. **a** *Tet*^*ON*^*-Plk4* MEFs were subjected to siRNA-mediated depletion of (**b**) Arhgap15 or (**c**) Atg16l1, alone or in (**d**) combination as indicated, in the absence or presence of doxycycline ( + Dox) to induce PLK4 overexpression. Cells expressing mCherry::EGFP::LC3B were stained for CEP164 and PLK4 to visualize autophagic vesicles (yellow to red, indicating autophagosome versus lysosome localization; insets labelled 1) and centrosomes (PLK4, magenta; CEP164, cyan; insets labelled 2). Representative immunofluorescence images are shown. Scale bar, 10 µm. **e** Quantification of the autophagosome:lysosome ratio under the indicated conditions, measured as the ratio of red (mCherry) to green (EGFP) pixel intensity. Data are from ***n*** = **11** independent biological replicates. Two-group comparisons were analysed using the Mann–Whitney U test. Bars and black error bars indicate mean ± 95% confidence interval (CI). Raw data are provided in Supplementary Data 6. **f** LC3B-II levels were quantified by densitometry from western blots to assess autophagic flux in Tet^ON^-*Plk4* MEFs cultured in the absence or presence of doxycycline ( + Dox), with or without bafilomycin A1, following RNAi with the indicated siRNAs. LC3B-II signal was normalized to the corresponding GAPDH levels and is shown as mean ± s.e.m. from *n* = 3 independent biological replicates. Two-group comparisons were analyzed using the Mann–Whitney U test. Corresponding western blots are shown in Supplementary Fig. 3a. Full-length uncropped blots are included in the Supplementary Information. Individual data points are provided in Supplementary Data 6.

To determine the roles of ARHGAP15 in autophagy, we monitored the effects of its depletion on the flux of mCherry- and EGFP-tagged LC3B, a protein recruited from the cytosol to the phagophore early in autophagy. The combined yellow fluorescence of these two tags marks autophagosome formation and the transition to solely red fluorescence reflects the fusion of autophagosomes with lysosomes where green fluorescence is quenched by the acidic environment. Treatment of *Tet*^*ON*^*-Plk4* MEFs with doxycycline increased the intensity of yellow fluorescent LC3B puncta in autophagosomes, also containing PLK4 and CEP164, while simultaneously enabling an increase in centrosome number (Fig. 4a, e). ARHGAP15 siRNA knockdown resulted in reduced numbers of PLK4- and CEP164-containing centrosomes and a dramatic increase in red fluorescent LC3B bodies in lysosomes, which showed some residual, colocalized PLK4 and CEP164 (Fig. 4b, e). Thus, ARHGAP15 depletion increases autophagy and the elimination of centrosomal antigens, thereby correcting the PLK4-mediated increase in centrosomes. In accord, the ratio of mCherry to EGFP fluorescence following ATG16L1 knockdown (Fig. 4c, e) or double knockdown of ATG16L1 and ARHGAP15 (Fig. 4d, e) indicates a decrease in autophagy. We also monitored autophagic flux by assessing autophagosome-associated LC3B-II levels following perturbation of ARHGAP15 or ATG16L1 function, in the presence or absence of doxycycline, and in comparison, with cells treated with bafilomycin A1, which blocks autophagosome–lysosome fusion (Fig. 4f, Supplementary Fig. 3a; Supplementary Data 6). ATG16L1 depletion resulted in reduced autophagic flux, whereas knockdown of *Arhgap15* led to an increase in flux. In line with our hypothesis that ARHGAP15 acts through ATG16L1, the combined depletion of ATG16L1 and ARHGAP15 inhibited autophagic flux. Together, these findings demonstrate that ARHGAP15 negatively regulates ATG16L1 activity during centrosome-associated autophagy.

**Fig. 5:**
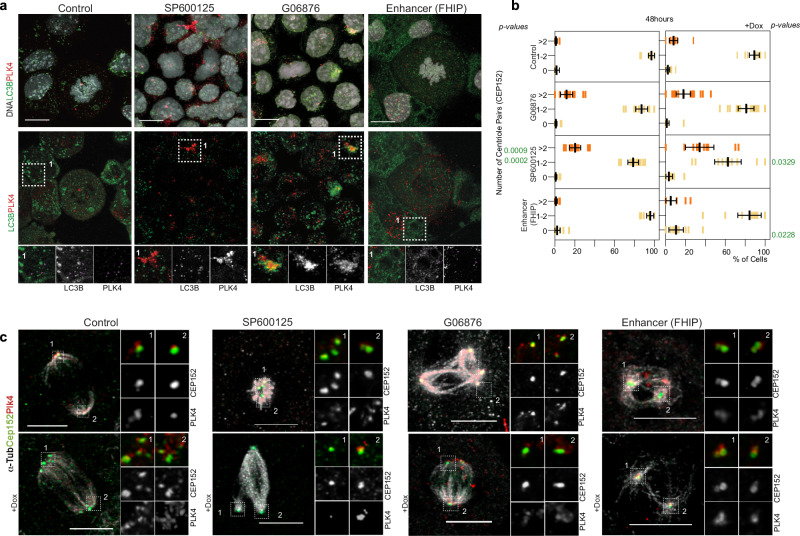
Inhibition of autophagy affects centrosome number. **a** Effects of inhibiting autophagy initiation with SP600125, blocking autophagosome–lysosome fusion with G06876, or enhancing autophagy with FHIP. LC3B (green) labels autophagic vesicles from early stages throughout the autophagy pathway. Cells were co-stained for PLK4 to visualize centrioles (red) and DAPI to stain DNA (white, upper panels). Control MEFs show circular LC3B-positive structures in the cytoplasm that are absent following SP600125 treatment, consistent with inhibition of phagophore formation. G06876 treatment results in large LC3B-positive vesicles containing PLK4. In FHIP-treated cells, LC3B-positive vesicles are smaller or undetectable. **b** Quantification of the percentage of cells with 0 and 1–2 (yellow bars), or >2 (orange bars), centriole pairs per cell. Data are from independent biological replicates: *n *= 17 for G06876 + Dox; *n* = 14 for G06876 and SP600125; *n* = 13 for Control, Control + Dox and SP600125 + Dox; and *n* = 12 for FHIP and FHIP + Dox. Black error bars indicate mean ± 95% confidence interval (CI). Statistical analysis was performed using the Kruskal–Wallis test followed by Dunn’s multiple comparisons test. *P* values are indicated in the figure: black, control −Dox versus control +Dox; green, control versus equivalent *Plk4*^OE^ conditions. Raw data and statistical analyses are included in Supplementary Data 7. **c** Representative mitotic figures of control and experimental groups treated with the indicated autophagy modulators in MEFs cultured in the absence or presence of doxycycline (Dox) and stained for PLK4 (red), CEP152 (green), and α-tubulin (white). Scale bar, 10 µm.

The above findings suggested that supernumerary centrosomes should accumulate if autophagy was to be blocked by other means. To test this, we treated *Tet*^*ON*^*-Plk4* MEFs with two different pharmacological inhibitors of autophagy: SP600125, which downregulates Beclin-1 preventing autophagophore formation^67^; and G06976, which blocks autophagosome-lysosome fusion and thereby autophagic flux^68^. SP600125 treated cells lacked the LC3B positive vesicles characteristic of mature phagosomes, and centrosomal components including PLK4 were visible at the centrosomes and as aggregates in the cell (Fig. 5a). By contrast, G06976 treated cells exhibited large LC3B vesicles that colocalized with multiple centrosomal components, including PLK4 (Fig. 5a). Both SP600125 and G06876 treatment led to centriole amplification within 48 hours irrespective of *Plk4* expression levels (Fig. 5b, c; Supplementary Data 7). Thus, pharmacological inhibition of autophagy results in an accumulation of centrosomal components in the autophagy pathway and permits formation of supernumerary centrosomes independently of PLK4 overexpression. In accord, treatment with FHIP, a p38 inhibitor^69^ resulted in enhancement of autophagy, and lysosome biogenesis such that LC3B positive vesicles became small or undetectable (Fig. 5a). We found that centrosomes were also undetectable following p38 inhibition in a significant percentage of cells overexpressing *Plk4* (Fig. 5b, c; Supplementary Data 7). These findings sustain the hypothesis that centrosomal components can be eliminated by autophagy to achieve centriole homeostasis.

### ULK1 activated ATG16L1 is required to eliminate supernumerary centrosomes

**Fig. 6:**
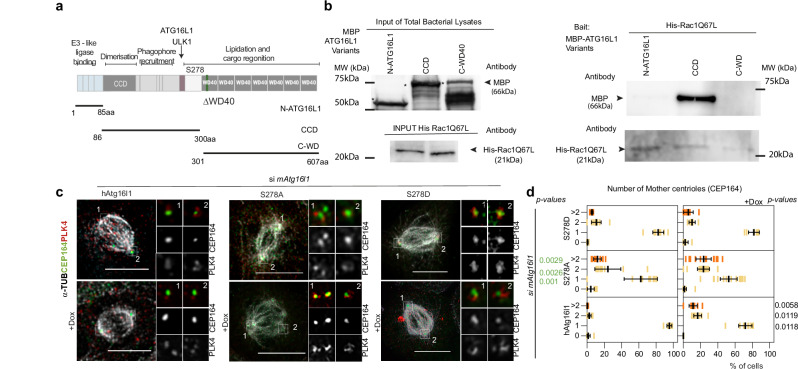
ATG16L1 binds RAC1 through the coiled-coil domain, and ULK1 kinase activates ATG16L1 in centrosome autophagy. **a** Schematic representation of the three functional domains of ATG16L1: the N-terminal alpha-helical region, required for binding the ATG5 E3-like ligase; the central region, required for homodimerization through the coiled-coil domain (CCD) and association with the phagophore; and the C-terminal domain containing seven WD40 repeats. Serine 278 (Ser278) is the ULK1 phosphorylation site that promotes autophagy. **b** Binding assay of purified recombinant His–RAC1 Q67L with three distinct ATG16L1 domains fused to MBP: N-terminal, CCD and WD. Lysates from bacteria expressing each MBP-tagged domain were incubated with His–RAC1 immobilized on cobalt beads. Input and pull-down fractions were analysed by SDS–PAGE and immunoblotting with anti-MBP and anti-His antibodies, revealing specific binding of His–RAC1 to the ATG16L1 CCD domain. Binding assays were performed in three independent experiments (***n*** = **3**) with reproducible results. Raw data are included in Supplementary Data 8. **c** TetON-Plk4 MEFs constitutively expressing EGFP-tagged human wild-type ATG16L1 (hATG16L1), EGFP-tagged human ATG16L1 S278A, or the phospho-mimetic ATG16L1 S278D were immunostained for the distal appendage protein CEP164 (green), PLK4 (red), and α-tubulin (white). Scale bar, 10 µm. **d** Quantification of the percentage of cells with 0, 1, 2, or >2 mother centrioles per cell. Individual values are shown as yellow bars, except for the >2 category, which is shown in orange. Data are from independent biological replicates: ***n*** = **12** for hATG16L1_S278A + si*Atg16l1* + Dox and hATG16L1_S278D + si*Atg16l1*; ***n*** = **11** for hATG16L1 + si*Atg16l1*, hATG16L1 + si*Atg16l1* + Dox, hATG16L1_S278A + si*Atg16l1* and hATG16L1_S278D + si*Atg16l1* + Dox. Black error bars indicate mean ± 95% confidence interval (CI). Statistical analysis was performed using the Kruskal–Wallis test followed by Dunn’s multiple comparisons test. *P* values are indicated in the figure: black, hATG16L1 versus hATG16L1 + Dox; green, hATG16L1 versus equivalent *Plk4*^OE^ conditions. Raw data and statistical analyses are included in Supplementary Data 8.

ATG16L1 is defined by three principal domains: an N-terminal ATG5/RB1CC1/ATG13-binding domain; a central coiled-coil dimerization domain (CCD); and a C-terminal region containing seven WD40 repeats (C-WD) (Fig. 6a). To gain mechanistic insight into how ARHGAP15 and ATG16L1 cooperate in centrosome homeostasis and autophagy, we examined the importance of these domains for their potential interactions. In vitro binding experiments of GST-ARHGAP15 and the GTP-locked small GTPase His-RAC1-Q67L confirmed previous findings that ARHGAP15 binds RAC1 isoform (Supplementary Fig. 4a, b)^64,70^. Strikingly, we found that ATG16L1 also interacts with RAC1-Q67L, placing ATG16L1 as a RAC1 interactor. Further mapping revealed that this interaction occurs through the central coiled-coil domain (CCD) of ATG16L1 (Fig. 6b).

ATG16L1 is activated by ULK1 kinase-mediated phosphorylation on S278^71^ (Fig. 6a). In accord, depletion of ULK1 promoted similar moderate levels of centrosome amplification as ATG16L1 depletion that were enhanced when PLK4 levels were elevated (Supplementary Fig. 4c, d). Whereas expression of wild-type human ATG16L1 in *Tet*^*ON*^*-Plk4* MEFs reverted the centrosome amplification following depletion of endogenous ATG16L1, expression of hATG16L1 S278A, which cannot be phosphorylated by ULK1, did not, resulting in increased cells with amplified centrosomes independent of PLK4 levels (Fig. 6c, d). On the other hand, the phosphomimic hATG16L1 S278D led to evasion of centrosome amplification (Fig. 6c, d; Supplementary Data 8).

**Fig. 7:**
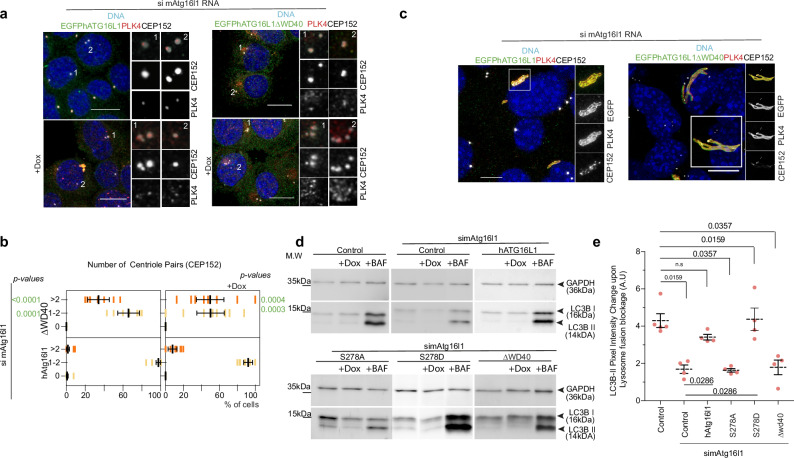
ULK1 kinase activates ATG16L1 in centrosome autophagy. **a** *Tet*^*ON*^*–Plk4* MEFs were stably transformed with either EGFP-tagged wild-type human (h)*Atg16l1* or its EGFP-tagged variant with a WD40 motif deletion, as described in the main text. Cells depleted of endogenous ATG16L1 were stained for CEP152 (white), PLK4 (red), and DNA (DAPI, blue). EGFP::ATG16L1 (green) appears as elongated vesicles close to the nucleus. Scale bar, 10 µm. **b** Graphic representation of the percentage of cells showing 0, 1–2, or >2 centrioles per cell (yellow and orange bars). Data are from independent biological replicates: *n* = 20 for h*Atg16l1* + si*mAtg16l1*; n = 14 for ΔWD40 + si*mAtg16l1* + Dox; *n* = 12 for h*Atg16l1* + si*mAtg16l1* + Dox; and ***n*** = **11** for ΔWD40 + si*mAtg16l1*. Black error bars indicate mean ± 95% confidence interval (CI). All values are provided in Supplementary Data 9. Statistical analysis was performed using the Kruskal–Wallis test followed by Dunn’s multiple comparisons test. P values are indicated in the figure: black, h*Atg16l1* + si*mAtg16l1* versus h*Atg16l1* + si*mAtg16l1* + Dox; green, h*Atg16l1* + si*mAtg16l1* versus equivalent *Plk4*^*OE*^ conditions. (**c**) Expression of EGFP::hATG16L1∆WD40 leads to large, reticulated vesicles. Vesicles observed in cells expressing wild-type hATG16L1 and hATG16L1∆WD40 contain PLK4 and CEP152 decorating the lumen. Scale bar, 10 µm. **d** Analysis of autophagic flux based on LC3B-II and LC3B-I levels in *Tet*^*ON*^*–Plk4* MEFs depleted of endogenous ATG16L1 and expressing the indicated hATG16L1 variants, cultured without or with doxycycline (+Dox) to induce PLK4 expression. Cells were analysed in the presence or absence of bafilomycin A1 (+BAF). **e** LC3B-II levels were quantified by densitometry and normalized to the density of the corresponding GAPDH band from the same western blot. Fold changes between each sample and the corresponding BAF-treated control are shown as mean ± SEM. These quantifications were performed from 3 independent biological replicates (*n* = 3). Two-group comparisons were analysed using the Mann–Whitney U test. Full-length uncropped blots, densitometry quantifications and statistical analyses are included in Supplementary Data 9.

We also established a stable *Tet*^*ON*^*-Plk4* MEF line expressing a mutant variant of human ATG16L1 with a deletion of its N-terminal-most WD40 repeat. Whereas wild-type hATG16L1 was able to rescue centriole numbers following knockdown of the endogenous mouse ATG16L1, the WD40 deleted variant could not (Fig. 7a–c; Supplementary Data 9). Thus, disruption of the N-terminal WD40 repeat results in loss of this function of ATG16L1. We observed that exogenous wild-type EGFP::hATG16L1 typically accumulated in an elongated phagophore-like vesicle close to the nucleus (Fig. 7b). EGFP::hATG16L1-∆WD40 was also present in phagophore-like vesicles that were even more prominent (Fig. 7b). In both cases, these vesicles accumulated PLK4 and its partner protein CEP152 but this was accentuated in cells expressing EGFP:: hATG16L1-∆WD40 (Fig. 7b).

To evaluate autophagic progression, LC3B-II levels were assessed by western blotting in cell lines depleted of endogenous ATG16L1 and reconstituted with wild-type ATG16L1, phospho-mutant (S278A), or phospho-mimetic ATG16L1 (S278D), in the presence or absence of bafilomycin A1 (Fig. 7d, e; Supplementary Data 9). As expected, ATG16L1 depletion reduced LC3B-II accumulation compared with controls, paralleling the effects observed with the WD40 deletion or the S278A mutant, both indicative of impaired autophagic flux. In contrast, expression of the phospho-mimetic ATG16L1 S278D promoted autophagy, whereas re-expression of wild-type ATG16L1 restored LC3B-II accumulation to control levels (Fig. 7d, e; Supplementary Data 9). Together, these experiments suggest that phosphorylation at serine 278 can activate ATG16L1 and promote centrosome autophagy.

### ARHGEF2 counteracts ARHGAP15 to regulate autophagy and centrosome numbers

**Fig. 8:**
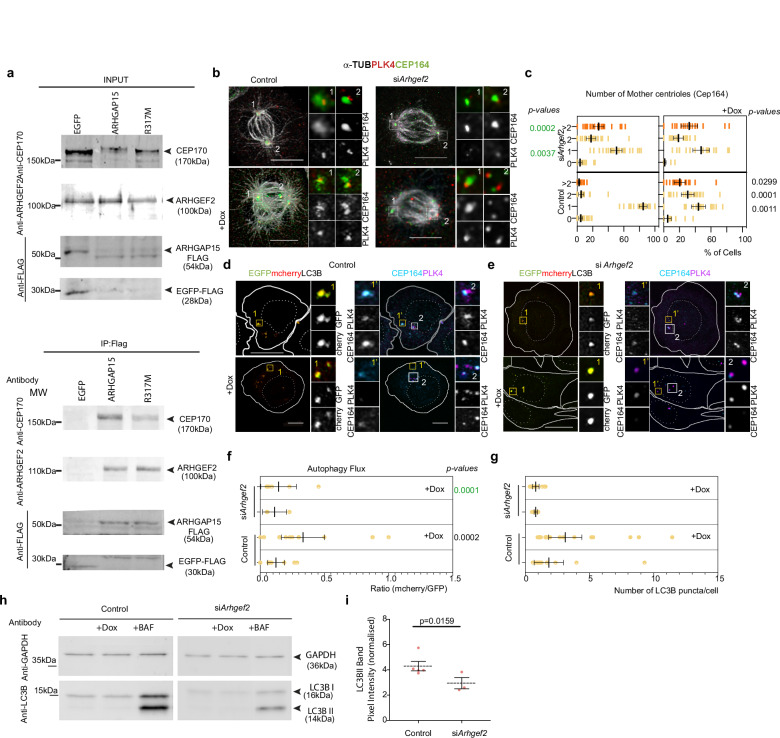
Regulation of centrosome number by the GTP exchange factor ARHGEF2. **a** *Tet*^*ON*^*–Plk4* MEFs were transfected with FLAG-tagged wild-type hARHGAP15, hARHGAP15 R317M (hR317M), or EGFP–FLAG control, as indicated. Extracts were subjected to anti-FLAG purification followed by western blot analysis. ARHGAP15 serves as a loading control. ARHGAP15 co-precipitates with ARHGEF2 and the centriolar protein CEP170. Pull-down and western blot experiments were performed independently three times (*n* = 3) with reproducible results. **b** Representative mitotic figures in the presence or absence of doxycycline (Dox)-induced Plk4 overexpression following siRNA-mediated knockdown of Arhgef2. Scale bars, 10 µm. Immunostaining shows centrosomal PLK4 (green), CEP164 (red), and spindle microtubules stained with anti-α-tubulin antibody (white). **c** Percentage of cells with 0, 1, 2, or >2 centrioles per cell. Individual values are shown as yellow bars for 0, 1, or 2 centrioles, and orange bars for >2 centrioles. Data are from independent biological replicates: *n *= 9 for Control and siA*rhgef2*; *n* = 7 for Control + Dox; and *n* = 6 for si*Arhgef2* + Dox. Black error bars indicate mean ± 95% confidence interval (CI). Statistical analysis used the Kruskal–Wallis test followed by Dunn’s multiple comparisons test. *P* values are indicated in the figure: black, control −Dox versus control +Dox; green, control versus samples with equivalent *Plk4*^*OE*^ conditions. Raw data and statistical analyses are included in Supplementary Data10. Representative images in the presence or absence of doxycycline-induced Plk4 expression in control MEFs (**d**) or after siRNA-mediated knockdown of *Arhgef2* (**e**). Immunostaining shows centrosomal PLK4 (magenta), CEP164 (cyan; right panels), and mCherry::EGFP::LC3B (red and green; left panels). Scale bars, 10 µm. **f** Ratio of red (mCherry) to green (EGFP) pixel intensity (A.U.). Individual values are shown as yellow circles. Data are from *n* = 12 independent biological replicates. Statistical analysis used the Kruskal–Wallis test followed by Dunn’s multiple comparisons test. *P* values are indicated in the figure: black, control −Dox versus control +Dox; green, control versus samples with equivalent *Plk4*^*OE*^ conditions. **g** Number of LC3B puncta per cell in the indicated conditions. Bars show mean ± 95% CI. Data are from *n *= 12 independent biological replicates. Statistical analysis used the Kruskal–Wallis test followed by Dunn’s multiple comparisons test. P values are indicated in the figure: black, control −Dox versus control +Dox; green, control versus samples with equivalent *Plk4*^*OE*^ conditions. **h**, **i** Analysis of autophagic flux by western blot showing LC3B-II levels after Arhgef2 siRNA knockdown, in the presence or absence of bafilomycin A1 ( + BAF), normalized to GAPDH from the same western blot. Fold changes between each sample and the corresponding BAF-treated control are shown as mean ± SEM. These quantifications were performed from 3 independent biological replicates (*n* = 3). Two-group comparisons used the Mann–Whitney U test. Full-length uncropped blots are included in the Supplementary Data 10.

To understand the centrosome signalling mechanism that could trigger a rise in RAC1-GTP and activate autophagy, we sought to identify a guanine-nucleotide exchange-factor (GEF) counteracting ARHGAP15. We focused on ARHGEF2, a RHO/RAC GEF that modulates cytoskeleton dynamics, which was pulled down with ARHGAP15, ATG16L1, and the centrosomal protein CEP170^72,73^ (Fig. 8a; Supplementary Data 4, Supplementary Fig. 5). Strikingly, depletion of ARHGEF2 from *Tet*^*ON*^*-Plk4* MEFs resulted in centriole amplification independently of PLK4 levels (Fig. 8b, c; Supplementary Data 10) as anticipated for a GEF counteracting ARHGAP15 in regulating centriole numbers. Cells displaying signs of cytokinesis failure associated with siArhgef2 depletion (binucleated, multinucleated nuclei, or markedly increased DAPI signal) were excluded from all quantifications (Supplementary Fig. 5a, b).

With the expectation that the depletion of a GEF counteracting ARHGAP15 would reduce autophagy, we ascertained the role of ARHGEF2 in the progression of autophagy, by assessing the fluorescence of mCherry::EGFP::LC3B stably expressed in *Tet*^*ON*^*-Plk4* MEFs as outlined above (Fig. 8d–g). Indeed, both the ratio of mCherry/GFP fluorescence (Fig. 8f) and the total number of mCherry::EGFP::LC3B puncta per cell (Fig. 8g; Supplementary Data 9) were lower following ARHGEF2 depletion than in control cells, indicating a reduction in LC3B lipidation and autophagosome formation (Fig. 8d–g; Supplementary Data 9). We also assessed LC3B-II levels by western blotting in the presence and absence of bafilomycin A1 (Fig. 8h, i). ARHGEF2 depletion resulted in reduced LC3B-II accumulation, indicating impaired autophagic flux, consistent with the findings described above.

Together, these findings suggest that ARHGEF2 is required in the initiation of autophagy and elongation of the autophagosomes, potentially linked to centrosome amplification.

### C*E*P170 subdistal appendage protein triggers ARHGEF2

The copurification of CEP170 with ARHGAP15 and ATG16L1 and with ARHGEF2^74,75^ suggested that this might be the centrosomal protein that signals autophagy. The distal appendage protein CEP170 is not required for centriole duplication but has been implicated in regulating microtubule organization and cell morphology^76^, We confirmed previous reports that CEP170 delocalizes from centrosomes following their amplification^76^; CEP170 localized with mother centrioles in 84.98% ± 4.09(sem)% control *Tet*^*ON*^*-Plk4* MEFs but was only detected in 31.15% ± 10.02(sem)% of centrioles following *Plk4* overexpression (Supplementary Fig. 6a, b; Source Data Supp. Fig. 6). Depletion of endogenous mouse CEP170 in cells expressing the tagged human protein showed that hCEP170 localized to LC3B vesicles and accumulated in autophagosomes following PLK4 overexpression (Supplementary Fig. 6c, d). Moreover, if elongation of autophagosomes was inhibited by ATG16L1 knockdown, hCEP170 aggregates appeared in the vicinity of LC3B positive puncta (Supplementary Fig. 6c).

**Fig. 9:**
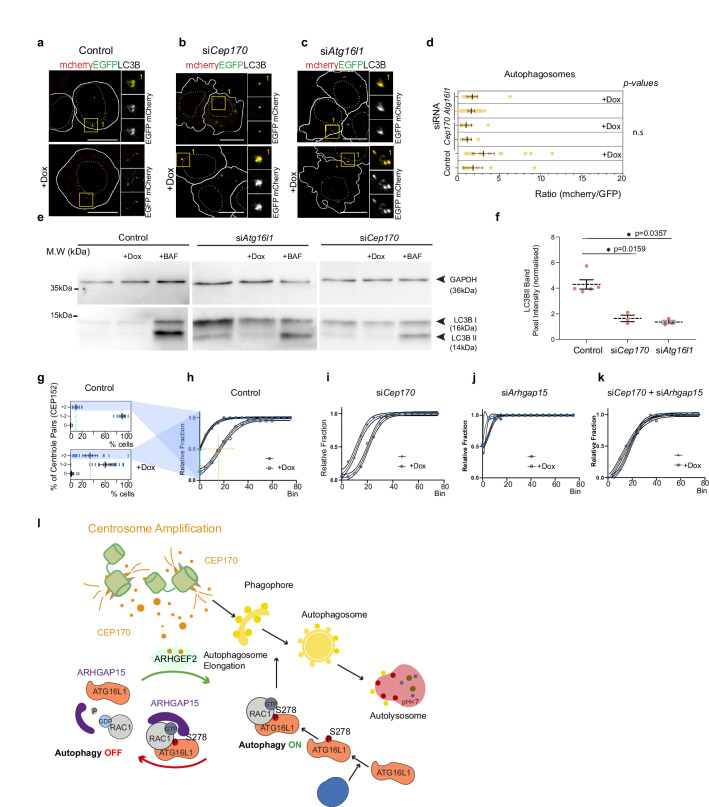
The relationship between CEP170 and regulators of autophagy. Representative mitotic figures in the presence or absence of doxycycline (Dox)-induced PLK4 expression in (**a**) control TetON-Plk4 MEFs expressing the mCherry::EGFP::LC3B autophagy reporter, (**b**) following siRNA-mediated knockdown of Cep170, and (**c**) following siRNA-mediated knockdown of Atg16l1. Staining reveals mCherry::EGFP::LC3B (red and green), as indicated. Scale bar, 10 μm. **d** Graphic representation of the ratio of red (mCherry) to green (EGFP) pixel intensity for each condition. Each value is shown by a yellow circle. Data are from n = 6 independent biological replicates. Two-group comparisons used the Mann–Whitney U test. Bars and black error bars indicate mean ± 95% CI. The raw data are included in Supplementary Data11. **e** Autophagic flux was analysed based on levels of phagophore/autophagosome-associated LC3B-II in extracts from TetON-Plk4 MEFs: control cells, cells depleted of endogenous ATG16L1, or cells depleted of endogenous CEP170. Cells were analysed in the absence or presence of bafilomycin A1 ( + BAF), and LC3B-II band intensity was quantified by densitometry and normalized to GAPDH from the same western blot. **f** Fold changes relative to the corresponding BAF-treated control are shown as mean ± SEM from three independent experiments (*n* = 3). Two-group comparisons used the Mann–Whitney U test. Full-length uncropped blots are included in theSupplementary Information. *P* values are shown in Supplementary Data11. **g** Cumulative frequency distribution of centrosome amplification (>2 centriole pairs per cell) before Plk4 overexpression (−Dox, blue filled circles) and after Plk4 overexpression (+Dox, open circles), corresponding to the data shown in Supplementary Fig.7. **h–k** The x axis indicates bins corresponding to the percentage of cells with >2 centrosomes, and the y axis indicates the cumulative fraction of cells within or below each bin. Points represent the mean of independent biological replicates. Solid lines show interpolated fitted curves, and dashed lines indicate the 95% confidence bands. Cumulative frequency distributions of centrosome amplification are shown in control MEFs (**h**) and TetON-Plk4 MEFs following siRNA-mediated knockdown of (**i**) Cep170, (**j**) Arhgap15, or (**k**) Cep170 and Arhgap15. **l** Model: supernumerary centrosomes release the centriolar protein CEP170 to activate ARHGEF2, which generates the active GTP-bound form of RAC1. RAC1-GTP binds to its effector ATG16L1, which has been pre-phosphorylated by ULK1 at Ser278. This initiates centrosome autophagy, allowing centrosome-containing autophagosomes (yellow) to elongate and direct their cargo to autolysosomes (red). RAC1 cycles to its GDP-bound inactive form through the action of ARHGAP15, which thus acts as a negative regulator of centrosome autophagy. RAC1-GDP, ARHGAP15 and ATG16L1 dissociate, and autophagy is inactivated.

To understand the relationship of CEP170 to autophagy, we analyzed autophagic progression by the ratio of red:green fluorescence of mCherry::EGFP::LC3B stably expressed in *Tet*^*ON*^*-Plk4* MEFs (Fig. 9a–d). We also assessed autophagic flux by western blot quantification of LC3B-II in extracts of MEFs following CEP170 siRNA knockdown, in the presence or absence of bafilomycin A1 (Fig. 9e). The levels observed after CEP170 depletion were comparable to those seen following ATG16L1 knockdown, and both were significantly different from wild-type MEFs. These results reveal that CEP170 depletion reduces autophagic flux to a similar extent as ATG16L1 knockdown (Fig. 9a–e; Supplementary Data 11). Thus, loss of CEP170 impairs autophagy to an equivalent degree as depletion of ATG16L1.

Together, the above findings suggested a role for CEP170 in triggering autophagy to regulate centriole numbers. This led us to relate the effects on centrosome numbers by impairing autophagy through CEP170 depletion to the activation of autophagy by depletion of ARHGAP15. To this end, we carried out RNAi as before (Supplementary Fig. 7 & Source Data Supp. Fig. 7), but also analyzed the cumulative frequency distribution of cells with greater than 2 centriole pairs (CEP152) to illustrate centrosome amplification (Fig. 9g–k, Supplementary Data 11). Depletion of CEP170 resulted in centrosome amplification in the absence of PLK4 ^OE^ in comparison to control cells and in both cases centrosome amplification was increased to similar levels following PLK4 ^OE^ (Fig. 9g–i). However, whereas ARHGAP15 knockdown prevented centriole over-replication in control *Plk4*^OE^ MEFs independently of PLK4 overexpression (Fig. 9j), centrosome amplification could not then be counteracted by ARHGAP15 knockdown in CEP170-depleted cells where it became independent of PLK4 ^OE^ (Fig. 9k). Thus, CEP170 function is required to generate the anticipated high levels of RAC1-GTP following ARHGAP15 knockdown or they fail to trigger autophagic elimination of centrosomes if the subdistal appendage protein CEP170 is also depleted (Fig. 9i, k; Supplementary Fig. 7; Supplementary Data 11). This points to a direct role for CEP170 in regulating the autophagic elimination of supernumerary centrosomes.

## Discussion

To understand why 85% of all cancers have cells that can proliferate with multiple centrosomes necessitates first understanding the response of the healthy mammalian cell to centrosome amplification. An earlier genome-wide screen for gene products that undertake surveillance of centrosome numbers used hTERT immortalized retinal pigment epithelial 1 (RPE1) cells additionally deleted for the *USP28* or *TRIM37* genes that enable cell proliferation following centrosome loss but not amplification^17^. The targeted nature of this previous screen identified a smaller number of proteins that respond to centrosome amplification that included PIDDosome components, particularly PIDD1^16^ and its centriolar distal appendage partner protein ANKRD26 alongside other distal appendage proteins^60,62^. The PIDDosome complex contains Caspase-2, which can cleave and inactivate MDM2, leading to p53 stabilization, in addition to promoting Caspase-2-mediated cell death^16,77^. Our current screen with ES cells, which have reduced checkpoint activities, has provided an unbiased means to identify unappreciated routes permitting cell proliferation in the presence of extra centrosomes. Our screen is vindicated through the identification of the PIDDosome^16^, and Tpr53-mediated pathways^25,74^ together with Hippo/Lats signalling, also previously implicated in centrosome surveillance^15,23,39^ (Fig. 1b, Supplementary Data 1). The Hippo responsive Lats kinase shows increased phosphorylation in tetraploid cells, which have extra centrosomes consequential to failed cytokinesis, and in cells with centrosome amplification. This results in phosphorylation and inactivation of YAP/TAZ transcriptional regulators, and inhibition of MDM2 to stabilize p53^15,75^. The central role played by p53 in centrosome surveillance has been evident from the hyper amplification of centrosomes in breast ductal carcinomas and squamous cell carcinomas of the head and neck coinciding with either mutation in p53 or Mdm2 overexpression^78^. We cannot exclude that reduced expression of some genes might affect proliferation independently of centrosome amplification. This is clearly the case for canonical cell-cycle regulators such as p53 or LATS2.

We also identified members of the Wnt pathway previously implicated to involve the centrosome^79,80^ through mechanisms that require clarification. Stabilization of β-catenin is required for Wnt signalling and Adenomatous Polyposis Coli protein (APC) mutant colorectal cancers show stabilization of β-catenin even when Wnt signalling is off, thereby activating the downstream pathway, The recent description of the association of a β-catenin destruction complex as a condensate associated with centrosomes marks a step towards understanding the interplay between centrosomes and the Wnt pathway^79^. The other proteins identified in our screen represent a variety of biological processes that includes, but is not limited to, centrosome-associated proteins such as AKTIP^57,58^; proteins that participate directly in centriole assembly including USP33, known to destabilize CP110 in S and G2 phase^60^; the centriolar protein, CETN3^61^; and multifunctional proteins such as the TRIM27 ubiquitin protein ligase^62,63,81,82^. In addition, we identified several small GTPases that were capable of regulating centrosome numbers and cell proliferation. The characterization of each of these cellular responses permitting cell proliferation in the presence of multiple centrosomes requires future study and is beyond the scope of the current report. Here, we have begun by focusing upon a previously unrecognized pathway regulating autophagy as a response that enables cell proliferation following the over production of centrioles.

Our findings underline a role for RAC1-GTP in regulating centrosome homeostasis and suggest the model depicted in Fig. 9i. Knockdown of GTPase activating, ARHGAP15, overcomes centrosome amplification induced by elevated PLK4 in a manner that depends upon the availability of autophagy protein, ATG16L1. Thus, ARHGAP15 is a negative regulator of autophagy that we show to be opposed by the guanine-nucleotide exchange factor ARHGEF2. We propose that loss of microtubule-associated CEP170^76^ from amplified centrosomes is one of the signals triggering ARHGEF2 activation to bring about the nucleotide exchange event that converts RAC1-GDP to its active GTP-bound form. The association of RAC1-GTP with ATG16L1 activates the autophagy pathway and can be promoted by ULK1-mediated phosphorylation at Ser 278, that activates the autophagy pathway and elongation of autophagosomes that leading to centrosome elimination upon fusion of the autophagosomes with lysosomes. ARHGAP15 is required to turn off centrosome autophagy by GTP hydrolysis resulting in dissociation of RAC1-GDP, ATG16L1 and ARHGAP15.

Our identification of CEP170, ARHGEF2, and ARHGAP15 as regulators of centrosome number through autophagy has not been previously recognized. Only two prior studies have suggested links between centrosome-associated proteins and autophagy. CEP63, a protein that interacts with CEP152^83^, the loading partner of PLK4, was reported to bind the selective autophagy receptor p62, although the mechanism by which CEP63 might activate selective autophagy^84^ remained unclear. In addition, the centrosomal satellite organizer PCM1 has been shown to associate with GABARAP through a C-terminal LC3-interacting region (LIR), leading to the accumulation of satellite aggregates when autophagy is inhibited^85^. Joachim and colleagues showed that PCM1 binding to GABARAP, but not to another ATG8 family member LC3B, required this LIR motif and that loss of PCM1 destabilized GABARAP via the proteasome^86^. They found that PCM1 enhances GABARAP/WIPI2/p62-positive autophagosome formation and flux but has no significant effect on LC3B-positive autophagosome formation suggesting one mechanism for centrosome-autophagosome crosstalk. It is of interest that PCM1 a centriolar satellite together with centriolar proteins were also part of the complex with ATG16L1 (Supplementary Data 3), suggesting they may be part of the same pathway. It should be noted, however, that the role of centriolar satellites in centriole biology remains elusive. However, CEP170 was the most abundant component we detected in association with ATG16L1, ARHGAP15 and ARHGEF2. Thus, our findings offer another step towards a mechanism whereby autophagy can regulate centrosome homeostasis in relation to sustained cell proliferation and viability.

Suppression of *Arhgap15* expression in malignant gliomas, colorectal, lung, pancreatic and breast cancer correlates with tumor grade or shorter disease-free survival^33,37,87^ and it has been proposed that ARHGAP15 acts as a tumor suppressor working through the PTEN/AKT/FOXO1-signaling pathway^33^. Our current study suggests that loss of ARHGAP15 in cancer cells should be associated with increased autophagy to eliminate supernumerary centrioles thereby overcoming the inhibitory effect on cell proliferation. This would add to the multiple roles of RAC1, principally known for regulating actin and microtubule dynamics and regulating cell movements and the migration and invasion of tumour cells^88–90^. In this context, our findings significantly impact our understanding of the multiple cellular processes that go awry in tumour cells and point to a need to consider pleiotropic and sometimes competing cellular events in the design of therapeutic strategies.

## Methods

### Establishment of mouse embryonic fibroblasts (MEFs)

Mouse embryonic fibroblasts (*Tet*^*ON*^*-Plk4* MEFs) were prepared from individual E13.5-14.5 embryos from *Plk4* ^*OE*^*/ Plk4* ^*OE*^ mice. Briefly, embryos were dissected from *decidua*, followed by the elimination of internal organs and by washes in PBS to eliminate blood. Embryos were mechanically disrupted and cultured in DMEM-Glutamax (Gibco, #10569-010) supplemented with 10% fetal bovine serum (Hyclone, #SH30071.03TH) with penicillin-streptomycin. The initial plating was defined as passage zero (P0), and cells were subsequently maintained and expanded following standard protocols.

### Pathway enrichment and network analysis

The hits from CRISPR-Cas9 screen were selected if they had been the target of at least two sgRNAs and had 1000 sequencing reads (Supplementary Data 1). The analysis of pathway enrichment was performed by consulting multiple open access databases including GO process, function and components, as well as Reactome and KEGG Pathways (Supplementary Data 1). Each protein network enrichment was then established using the STRING algorithm and database^91^. The identified nodes were exported, integrated and mapped using Cytoscape v.3.8.0^92^. The networks were scrutinized for presence of nodes. For visualization, the yFiles organic layout algorithms were used and redeployed manually.

### *Re-screening of target genes* by transfecting MEFs with siRNA

MEFs were used to re-screen targets identified in the CRISPR-Cas9 screening. To knock down the expression of each gene, siRNA was transfected into MEFs in a 24-well format. Each transfection mixture consisted of 6 pmol siRNA duplex (siRNA sequences are detailed in Supplementary Data 12) and 1 µl Lipofectamine RNAi MAX (Invitrogen, #13778075) in 100 µl Opti-MEM (Gibco, #31985062) per tube. The diluted RNAi molecules were gently mixed, added to every well, and incubated for 20 min at room temperature. MEFs were then diluted to 4 × 10^4^ cells per mL in DMEM complete growth medium and 500 µL of MEFs were added per well and mixed with the siRNA mixture. Doxycycline was added 24 h later to a final concentration of 4 µg/mL and replaced after 48 h. Cells were counted and fixed 96 h after transfection with siRNA. The calculation of cell proliferation recovery in response to siRNA treatment with doxycycline (DOX) is normalized against cell growth in siRNA treated cells without doxycycline and compared with the variation observed in MEFS with and without doxycycline, according to the formula: 100 x [(Gene query siRNA_+DOX_ / Gene query siRNA) - (Control_+DOX_/Control)]. This recovery value reflects how much the presence of DOX affects cell proliferation in siRNA-treated cells relative to the control.

### CRISPR-Cas9 screen and sequencing

*Plk4*^*OE*^ mES cell lines expressing Cas9-GFP were generated by lentiviral transduction with pLenti-Cas9-GFP. Lentivirus was produced in HEK-293T cells by co-transfection of pLenti-Cas9-GFP (Supplementary Data 12), the envelope plasmid pMD2.G and the lentiviral packaging plasmid psPAX2 using TransIT-293 Transfection Reagent (MirusBio, Cat. MIR 2704) (Supplementary Data 12). Fluorescence-activated cell sorting (FACS) of GFP-positive mES cells was performed to isolate single-cell clones. In parallel, packaged lentivirus containing the pooled sgRNA-lentiGuide library was generated in HEK-293T cells by co-transfection with pMD2.G and psPAX2, as described above. Lentiviral supernatants were harvested 48 h after transfection and filtered through a syringe-mounted 0.45 μm filter (Merck Millipore). A total of 1 × 10¹⁰ *Plk4*^*OE*^ ESC cells were plated across twelve 15 cm tissue-culture dishes coated with 0.1% gelatine 24 h before viral infection, to allow cells to reach 70% confluence on the day of infection. *Plk4*^*OE*^ ESC cells were infected with a mouse genome-wide guide RNA (gRNA) lentiviral library at a multiplicity of infection of 0.1–1.5 and a library coverage of 1000-fold. Fresh culture medium containing 8 μg/mL polybrene was added with lentiviral particle suspension at 500 μL per plate. Infected and non-infected mouse embryonic stem cells were incubated overnight at 37 °C in 5% CO₂. DMEM-Glutamax medium (Gibco, #10569-010) containing 10% FBS (Hyclone, #SH30071.03TH) was changed 24 h after infection and every two days thereafter. Plk4 overexpression was induced by adding doxycycline four days after infection, at a final concentration of 4 μg/mL, to the 12 infected and 12 uninfected dishes. Medium and doxycycline were changed every two days for 13 days, until all doxycycline-treated uninfected cells had died. At the end of the experiment, cells were harvested and frozen at −20 °C. Genomic DNA was prepared using the QIAamp DNA Blood Kit (Qiagen, Cat. #51192) for PCR amplification of sgRNAs present in the samples. The Mouse Two Plasmid Activity-Optimized CRISPR Knockout Library was a gift from David Sabatini and Eric Lander (Addgene #1000000096). Amplification was carried out following the authors’ protocol provided by Addgene. Primers used are listed in Supplementary Data 12.

### Immunoprecipitation and mass spectrometry analysis

Flash-frozen cell pellets were thawed on ice at 4 °C and resuspended in 1 ml cell lysis buffer containing 10 mM Tris-HCl pH 7.2, 140 mM NaCl, 1 mM EDTA, 0.5 mM EGTA, 0.1% Triton X-100 and 10% glycerol, freshly supplemented with Benzonase diluted 1:1000 (Sigma/Millipore, #E1014), 1 mM PMSF and cOmplete EDTA-free protease inhibitor cocktail (Sigma/Millipore, #4693132001). The resuspension was sonicated at 20 kHz for no longer than 30 s and centrifuged at 700 × *g* for 15 min at 4 °C. The lysis buffer was also used as the washing buffer in all immunoprecipitation assays. The supernatant was transferred to a fresh tube, and the pellet was discarded. For immunoprecipitation of C-terminally tagged ARHGAP15, anti-FLAG magnetic agarose beads (Pierce/Thermo Fisher Scientific, #A36797) or Myc-Trap Magnetic Agarose beads (Chromotek, #ytma) were equilibrated with lysis buffer according to the manufacturers’ instructions. For ATG16L1-GFP precipitation, GFP-Trap magnetic agarose beads (Chromotek, #gtma) were used. Beads were diluted in lysis buffer to 1 mg/ml, and 50 µl of bead suspension was used per sample. The protein–bead–antibody mixture was incubated on a rotary shaker overnight at 4 °C. Samples were then washed three times for 10 min each in 1 ml lysis buffer at room temperature, followed by a final wash with PBS for 5 min on the rotary shaker at room temperature. Input and flow-through samples were prepared by adding the appropriate amount of protein and 4× SDS loading buffer. These samples were heated at 95 °C for 5 min, loaded onto 7% or 15% Bio-Rad Tris-Glycine gels, and subjected to electrophoresis at 90 V for 1 h 30 min. For immunoprecipitated protein samples analysed by mass spectrometry, an equivalent IP protocol was followed but scaled up. Proteins were kept associated with the beads at 4 °C until proteolytic digestion before mass spectrometry analysis.

Mass spectrometry was performed at the Mass Spectrometry Laboratory, Institute of Biochemistry and Biophysics, Polish Academy of Sciences (IBB PAS), using an Orbitrap Exploris 480 mass spectrometer. Raw data were pre-processed using Mascot Distiller version 2.7 (Matrix Science Ltd.) and searched using Mascot Search Engine version 2.7 (Matrix Science Ltd.) against *Mus musculus* or *Homo sapiens* protein sequences derived from Swiss-Prot version 2021_03. The databases contained 17,089 *Mus musculus* sequences. Searches were performed with the following parameters: enzyme, trypsin; fixed modification, methyl Thio (C); variable modification, oxidation (M); peptide mass tolerance, 5 ppm; fragment mass tolerance, 0.01 Da; and up to two missed cleavages. Files were re-calibrated offline using results from preliminary searches. The false discovery rate was calculated using the target-decoy strategy implemented in Mascot and kept below 1%. The immunoprecipitated samples analysed, corresponding two processed protein-identification results are listed in Supplementary Data 4. The mass spectrometry proteomics data have been deposited to the ProteomeXchange Consortium via the PRIDE partner repository with the dataset identifier PXD077984 and 10.6019/PXD077984. Two immunoprecipitation mass spectrometry datasets were analysed, corresponding to EGFP–ATG16L1 and ARHGAP15 immunoprecipitations. The analysed samples, controls and processed protein-identification results are listed in Supplementary Data 4.

### Expression of ATG16L1 and ARHGAP15

Recombinant MBP fusions of wild-type or R317M mutant ARHGAP15 and GST fusions of wild-type or S278A/D mutant ATG16L1 were expressed and purified from yeast using an auto-selection expression system^93^. PCR cassettes encoding the relevant ORF, flanked by sequences homologous to the ends of the gapped pMG3 expression vector for MBP–ARHGAP15 or the pMH925 expression vector for GST–ATG16L1, were used for gap-repair transformation of the host strain MGY853 (MATa ura3-1 trp1-28 leu2Δ0 lys2 his7 cdc28::LEU2 pep4::LEU2 [URA3-CDC28]). Transformants were plated on 5-FOA to counter-select the resident CDC28-carrying plasmid. Thereafter, the expression construct was maintained in rich medium to allow efficient protein expression. Protein expression was induced in YEP-galactose for 8 h at 30 °C. Cells were harvested, rinsed with ice-cold water, snap-frozen and stored at −80 °C. Pellets were resuspended in cold breaking buffer at 1/20 of the original cell culture volume. Breaking buffer contained 50 mM Tris pH 7.5, 250 mM NaCl, 0.2% NP-40, 10% v/v glycerol, 5 mM EDTA, Roche cOmplete EDTA-free protease inhibitor, 1 mM PMSF and 5 mM DTT. Cells were disrupted in a FastPrep-24™ grinder (MP Biomedicals) using 0.5 mm glass beads. Cell lysates were cleared by two rounds of centrifugation at 16,000 × *g* for 5 min at 4 °C. Amylose resin (New England Biolabs) or Glutathione Sepharose 4B (Cytiva) was added and allowed to bind for 2 h on a rotator at 4 °C. The resin was washed six times with washing buffer containing 50 mM Tris pH 7.5, 250 mM NaCl, 0.2% NP-40 and 1 mM DTT. Where required, proteins were eluted with 20 mM maltose or reduced glutathione, respectively.

### In vitro pull-down assay with MBP and GST resins

In vitro pull-down assays were performed using either amylose resin beads (MBP-Binding Resin, New England Biolabs) or Glutathione–Sepharose 4B beads (GE Healthcare). MBP-Binding Resin was preloaded with MBP-tagged proteins (MBP–ARHGAP15, MBP–ATG16L1 or MBP as control), whereas Glutathione–Sepharose 4B beads were preloaded with GST-tagged proteins (GST–ATG16L1 or GST as control). Beads were washed in buffer A containing 20 mM Tris–HCl pH 7.5, 150 mM NaCl, 5% v/v glycerol, 1 mM DTT and 0.1% v/v Triton X-100, and blocked for 45 min at 4 °C with 10 mg/ml BSA. After removal of the blocking solution, beads were washed once in buffer A and incubated with the indicated prey proteins for 1 h at 4 °C with gentle rotation. Beads were subsequently washed three times for 10 min each with buffer A. Bound proteins were eluted by boiling in Laemmli sample buffer, separated by SDS–PAGE, and analyzed by PageBlue protein staining (Thermo Fisher Scientific) and immunoblotting with anti-MBP, anti-GST, anti-ATG16L1 and anti-His antibodies.

### In vitro binding assay with cobalt affinity resin

In vitro binding assays were also performed using cobalt affinity resin (HisPur™ Cobalt Resin, Thermo Fisher Scientific, #89964) preloaded with His–RAC1 Q67L. Resin was washed in buffer A and blocked for 1 h at 4 °C with 10 mg/ml BSA. After removal of the blocking solution, resin was washed once in buffer A and incubated with MBP-tagged ATG16L1 fragments (MBP–N-ATG16L1, MBP–CCD or MBP–C-WD) for 1 h at 4 °C with gentle rotation. Resin was subsequently washed three times for 10 min each in buffer A. Bound proteins were eluted by boiling in Laemmli sample buffer, separated by SDS–PAGE, and analyzed by PageBlue protein staining and immunoblotting with anti-MBP and anti-His antibodies. Details included in Supplementary data 12.

### Immunoblotting

Cells were scraped from dishes in 4× Laemmli sample buffer (Bio-Rad, #1610747). After total protein quantification, equal amounts of protein were subjected to electrophoresis on 7% or 15% Tris-Glycine acrylamide gels (Bio-Rad, 30% acrylamide/bis 29:1, #1610156), transferred to nitrocellulose membranes (Bio-Rad, #1704150) and immunoblotted with the indicated primary and secondary antibodies (Supplementary Data 12).

### Immunofluorescence

Cells were grown on coverslips, washed with 1× PBS after removal of the medium, and fixed in cold methanol at −20 °C for at least 12 min. Cells were rehydrated in 1× PBS, followed by permeabilization in PBS containing 0.5% Triton X-100 for 15 min and three washes for 10 min each in PBS containing 0.1% Triton X-100. Blocking was performed in PBS containing 0.1% Tween-20 and 10% FBS for 1 h, followed by incubation with primary antibodies overnight at 4 °C and secondary antibodies for 1 h at room temperature in PBS containing 0.1% Tween-20 and 10% FBS. Washes were performed using PBS containing 0.1% Tween-20. Coverslips were mounted in Vectashield Mounting Medium with DAPI (Vector Laboratories, H1200-10). Images were collected on a Leica SP8 Stellaris with 63×/1.4 oil objectives using Leica Application Suite X software (LAS-X). Images were deconvolved with Huygens Professional version 19.04 (Scientific Volume Imaging, The Netherlands); processing and analysis were performed with ImageJ version 1.53a and Adobe Illustrator 2024. All images shown are projections of all Z optical sections, acquired with a Z-step of 0.5 µm.

### Statistics and reproducibility

No statistical method was used to predetermine sample size. Experiments were performed with at least three independent biological replicates, unless otherwise stated in the figure legends. For microscopy-based quantifications, at least 50 cells were quantified per biological replicate from multiple randomly selected microscopy fields. The total number of cells quantified for each condition is indicated in the corresponding source data. To quantify centriole number revealed by CEP152 or CEP164 staining, cells were scored from randomly selected microscopy fields and assigned to the indicated categories: 0, 1, 2, or >2 centrioles. The percentage of cells in each category was calculated for each replicate and is shown in the source data. Where indicated, individual microscopy fields are shown in the graphical representation of the data; statistical analyses were performed on biological replicate-level values unless otherwise stated. No data were excluded from the analyses. For the experiments described in Fig. 1b, c and Fig. 2, samples were coded and blinded to gene identity during outcome assessment. Normality was not assumed. Statistical analyses were performed using the Kruskal–Wallis H test to assess differences among groups. When significant differences were detected, post-hoc pairwise comparisons were performed using Dunn’s multiple comparisons test with Bonferroni correction. All statistical tests were two-sided, unless indicated otherwise in figure legends. Exact n values, statistical tests and P values are provided in the corresponding source data.

### Graphical representation of centriole number

Individual microscopy fields are represented as bars in the graphical representation of the data, with yellow indicating cells with 0–2 centrioles and orange indicating cells with >2 centrioles. Data were plotted using GraphPad Prism version 10.6.0. Data represent at least three independent biological replicates, unless otherwise stated in the figure legends. The mean is shown with a black bar, and error bars represent the mean ± 95% confidence interval (CI).

### LC3B western blotting for quantifying autophagic flux

Cells were treated with bafilomycin A1 (Baf A1; Cell Signaling Technology, #50-205-0494) at 1 µM for 16 h. Cells were washed with ice-cold PBS and lysed in RIPA buffer containing protease and phosphatase inhibitors. Western blotting was performed using membranes sequentially incubated with antibodies to detect LC3B and GAPDH. Band intensities were quantified using Image Lab v6.1.0 (Bio-Rad) from ChemiDoc MP images and normalized to GAPDH from the same membranes. Individual data points are provided in the Supplementary Data indicated for each figure. Quantifications were performed from three independent biological replicates. Two-group comparisons were analyzed using the Mann–Whitney U test. Full-length uncropped blots are included in the Supplementary Information.

### qRT-PCR analysis

RNA samples were prepared using the Qiagen RNeasy Micro Kit (#74004). Primer pairs were designed for each specific gene and are listed in Supplementary Data 12. qRT-PCR was performed using the Luna Universal One-Step RT-qPCR Kit (New England Biolabs) according to the manufacturer’s instructions. Amplification was monitored with SYBR Green chemistry on an Applied Biosystems StepOnePlus Real-Time PCR System. Each reaction was run in triplicate. Gene expression levels were normalized to GAPDH as the housekeeping gene, and relative expression was calculated using the 2^−ΔΔCt^ method. For statistical analysis, relative expression values were analyzed using one-way ANOVA to determine overall group differences. Post-hoc comparisons between groups were performed using Tukey’s multiple comparisons test. All statistical analyses were carried out in GraphPad Prism 10.3.0.

### Animal ethics

All animal work was conducted in compliance with the relevant institutional and national regulations governing the use of animals in research. Experiments performed in the United Kingdom were authorized by the UK Home Office under Project License 70/8453. Experiments performed at the California Institute of Technology were approved by the Institutional Animal Care and Use Committee under protocol 1874.

### Reporting summary

Further information on research design is available in the Nature Portfolio Reporting Summary linked to this article.

## Supplementary information

Supplementary Information

Description of Additional Supplementary Files

Supplementary Data 1

Supplementary Data 2

Supplementary Data 3

Supplementary Data 4

Supplementary Data 5

Supplementary Data 6

Supplementary Data 7

Supplementary Data 8

Supplementary Data 9

Supplementary Data 10

Supplementary Data 11

Supplementary Data 12

Reporting Summary

Transparent Peer Review file

## Source data

Source Data

## Data Availability

The mass spectrometry proteomics data generated in this study have been deposited to the ProteomeXchange Consortium via the PRIDE partner repository with the dataset identifier **PXD077984** and 10.6019/PXD077984. The dataset is publicly available at: https://www.ebi.ac.uk/pride/archive/projects/PXD077984. Source data are provided with this paper.
