# Supplementary material for: Sensing centrosome amplification: the interface between centriole duplication and autophagy
Source: Nat Commun. 2026 Jun 21;17:7815. doi: 10.1038/s41467-026-74702-9 (PMC13439419; doi:10.1038/s41467-026-74702-9)
Supplement: Supplementary file 1 [file 41467_2026_74702_MOESM15_ESM.pdf]

nature portfolio

Corresponding author(s): Paula Almeida Coelho
David M. Glover

Last updated by author(s): May 7, 2026

Reporting Summary

Nature Portfolio wishes to improve the reproducibility of the work that we publish. This form provides structure for consistency and transparency in reporting. For further information on Nature Portfolio policies, see our Editorial Policies and the Editorial Policy Checklist.

Statistics

For all statistical analyses, confirm that the following items are present in the figure legend, table legend, main text, or Methods section.

n/a	Confirmed	
☐	☒	The exact sample size (*n*) for each experimental group/condition, given as a discrete number and unit of measurement
☐	☒	A statement on whether measurements were taken from distinct samples or whether the same sample was measured repeatedly
☐	☒	The statistical test(s) used AND whether they are one- or two-sided<br>*Only common tests should be described solely by name; describe more complex techniques in the Methods section.*
☐	☒	A description of all covariates tested
☐	☒	A description of any assumptions or corrections, such as tests of normality and adjustment for multiple comparisons
☐	☒	A full description of the statistical parameters including central tendency (e.g. means) or other basic estimates (e.g. regression coefficient) AND variation (e.g. standard deviation) or associated estimates of uncertainty (e.g. confidence intervals)
☐	☒	For null hypothesis testing, the test statistic (e.g. F, t, r) with confidence intervals, effect sizes, degrees of freedom and P value noted<br>*Give P values as exact values whenever suitable.*
☒	☐	For Bayesian analysis, information on the choice of priors and Markov chain Monte Carlo settings
☐	☒	For hierarchical and complex designs, identification of the appropriate level for tests and full reporting of outcomes
☒	☐	Estimates of effect sizes (e.g. Cohen's d, Pearson's r), indicating how they were calculated

Our web collection on statistics for biologists contains articles on many of the points above.

Software and code

Policy information about availability of computer code

Data collection	Leica software and Fiji Version 2.16.0/1.54p, Adobe Creative Cloud (Version 6.9.11)
Data analysis	GraphPad Prism Version 10.6.1

For manuscripts utilizing custom algorithms or software that are central to the research but not yet described in published literature, software must be made available to editors and reviewers. We strongly encourage code deposition in a community repository (e.g. GitHub). See the Nature Portfolio guidelines for submitting code & software for further information.

Data

Policy information about availability of data

All manuscripts must include a data availability statement. This statement should provide the following information, where applicable:

- Accession codes, unique identifiers, or web links for publicly available datasets
- A description of any restrictions on data availability
- For clinical datasets or third party data, please ensure that the statement adheres to our policy

The mass spectrometry proteomics data generated in this study have been deposited to the ProteomeXchange Consortium via the PRIDE partner repository under accession code PXD077984 and DOI 10.6019/PXD077984. The dataset is publicly available at: https://www.ebi.ac.uk/pride/archive/projects/PXD077984.

Research involving human participants, their data, or biological material

Policy information about studies with human participants or human data. See also policy information about sex, gender (identity/presentation), and sexual orientation and race, ethnicity and racism.

Reporting on sex and gender	N/A.
Reporting on race, ethnicity, or other socially relevant groupings	N/A
Population characteristics	N/A
Recruitment	N/A
Ethics oversight	N/A

Note that full information on the approval of the study protocol must also be provided in the manuscript.

Field-specific reporting

Please select the one below that is the best fit for your research. If you are not sure, read the appropriate sections before making your selection.

☒ Life sciences ☐ Behavioural & social sciences ☐ Ecological, evolutionary & environmental sciences

For a reference copy of the document with all sections, see nature.com/documents/nr-reporting-summary-flat.pdf

Life sciences study design

All studies must disclose on these points even when the disclosure is negative.

Sample size	Sample sizes are indicated in the corresponding figure legends and Source Data or Supplementary Data files. No statistical method was used to predetermine sample size.
Data exclusions	No data were excluded from the analyses.
Replication	All experiments were carried out at least three times independently, unless otherwise indicated. The number of replicates is indicated for each experiment in the figure legends and corresponding Source Data or Supplementary Data files.
Randomization	Samples and fields of view were selected randomly where applicable. Quantification was performed according to predefined criteria.
Blinding	The reconfirmation of the screening with the initial candidate genes was performed under blinded conditions, with the experimenters unaware of the gene identities (Figure 1 and Extended Data Figure 1). All subsequent experiments were conducted after unblinding and following the analysis of control samples.

Reporting for specific materials, systems and methods

We require information from authors about some types of materials, experimental systems and methods used in many studies. Here, indicate whether each material, system or method listed is relevant to your study. If you are not sure if a list item applies to your research, read the appropriate section before selecting a response.

Materials & experimental systems

n/a	Involved in the study	
☐	☒	Antibodies
☐	☒	Eukaryotic cell lines
☐	☐	Palaeontology and archaeology
☐	☒	Animals and other organisms
☐	☐	Clinical data
☐	☐	Dual use research of concern
☐	☐	Plants

Methods

n/a	Involved in the study	
☐	☐	ChIP-seq
☐	☐	Flow cytometry
☐	☐	MRI-based neuroimaging

Antibodies

Antibodies used	All the information is included in Supplementary Data Table 12 with links and catalogue numbers clearly provided. However, please

Antibodies used	find here the entire list.<br>Anti-alpha-tubulin Mouse Sigma-Aldrich Cat# T6199, RRID:AB_ 477583 1.5000 (IF) & (WB)<br>Anti-alpha-tubulin Guinea Pig ABCD Antibodies Cat# abcd_aa345, RRID:AB_3106979 1:2000 (IF)<br>Anti-ArhGap15 Rabbit Thermo Fisher Scientific Cat# PA5-100389, RRID:AB_2849901 1:1000 (WB)&(IF)<br>Anti-ArhGap15 Rabbit Proteintech Cat# 11940-1-AP, RRID:AB_874081 1:1000 (WB)<br>Anti-Arhgef2/GEF-H1 Rabbit Thermo Fisher Scientific Cat# PA5-32213, RRID:AB_2549686 1:1000 (WB)&(IF)<br>Anti-ATG16L1 Rabbit Abcam Cat# ab187671 1.1000 (IF)<br>Anti-ATG16L1-phospho_ser278 Rabbit Cell Signaling Technology Cat# 84300, RRID:AB_2915984 1:1000 (WB)<br>Anti-Cep152 Rabbit Abcam Cat# ab183911, RRID:AB_3106957 1.1000 (IF)<br>Anti-Cep164 Rabbit Proteintech Cat# 22227-1-AP, RRID:AB_2651175 1.1000 (IF)<br>Anti-CEP170 Rabbit Proteintech Cat# 27325-1-AP, RRID:AB_2880844) 1:1000 (WB)&(IF)<br>Anti-CEP170 Mouse Thermo Fisher Scientific Cat# 41-3200, RRID:AB_2533502 1:1000 (WB)&(IF)<br>Anti-Flag (M2) Mouse Cell Signaling Technology Cat# F3165, RRID:AB_259529 1:5000 (WB)<br>anti-γ-tubulin Rabbit Sigma-Aldrich Cat# T3559, RRID:AB_477575 1:2000 (IF)<br>FluoTag®-X4 anti-GFP Alpaca NanoTag Biotechnologies Cat# N0304-AT488-L, RRID:AB_2744629 1.1000 (IF)<br>Anti-GFP Rabbit Abcam Cat# ab290, RRID:AB_303395 1:1000 (WB)<br>Anti-GST 3G10/1B3 Mouse Abcam Cat#: ab92; RRID:AB_307067 1:1000 (WB)<br>anti-GTU88 Mouse Sigma-Aldrich Cat# T6557, RRID:AB_477584 1.1000 (IF)<br>Anti-His Mouse Qiagen Cat# 34660, RRID:AB_2619735 1:1000 (WB)<br>Anti-LC3B Rabbit Abcam Cat#: ab192890, RRID:AB_2827794 1:2000(IF); 1:3000(WB)<br>Anti-MBP Mouse New England Biolabs Cat# E8032, RRID:AB_1559730 1:1000 (WB)<br>Anti-Myc Mouse Santa Cruz Biotechnology Cat# sc-40, RRID:AB_627268 1:500 (IF)<br>Anti-Plk4 Rat Coelho et al., (2013) N/A 1.1000 (IF)<br>Anti-p62 /SQTM1 Rabbit Proteintech Cat# 18420-1-AP, RRID:AB_10694431 1:5000(WB)<br>Anti-p62 /SQTM1 Rabbit Abcam Cat# 88588, RRID:AB_2800125 1:5000 (WB); 1:1000(IF).<br>The secondary antibodies used:<br><br>Anti-pericentrin Mouse BD Biosciences Cat# 611815, RRID:AB_399295 1.1000 (IF)<br>Anti-ULK1 (D8H5) Rabbit Cell Signaling Technology Cat# 8054, RRID:AB_11178668 1:200 (WB)<br>Anti-USP33 Rabbit OriGene Cat# TA383160, RRID:AB_3106965 1:1000 (WB)
Validation	All the antibodies, with the exception of the one generated by our lab, validated and published prior to this publication (Coelho et al., 2013), are registered antibodies widely used. The registration number is indicated.registered antibodies

Eukaryotic cell lines

Policy information about cell lines and Sex and Gender in Research

Cell line source(s)	Two types of cell lines were used in this publication: Mouse Embryonic Stem Cells (mESC) and mouse Embryonic Fribroblasts (MEFs).
Authentication	The source for mouse stem cell lines is provided- Sanger Institute, William Skarnes, UK. The mESCs were published previously (Coelho et al., 2015). The MEFs were prepared for this work and the source is the Transgenic mice generated and described previously.
Mycoplasma contamination	All cell lines were regularly tested and confirmed negative for mycoplasma contamination using the Mycoplasma Detection Kit (SouthernBiotech, Ref. 13100-01).
Commonly misidentified lines<br>(See ICLAC register)	*Name any commonly misidentified cell lines used in the study and provide a rationale for their use.*

Palaeontology and Archaeology

Specimen provenance	*Provide provenance information for specimens and describe permits that were obtained for the work (including the name of the issuing authority, the date of issue, and any identifying information). Permits should encompass collection and, where applicable, export.*
Specimen deposition	*Indicate where the specimens have been deposited to permit free access by other researchers.*
Dating methods	*If new dates are provided, describe how they were obtained (e.g. collection, storage, sample pretreatment and measurement), where they were obtained (i.e. lab name), the calibration program and the protocol for quality assurance OR state that no new dates are provided.*

☐ Tick this box to confirm that the raw and calibrated dates are available in the paper or in Supplementary Information.

Ethics oversight	*Identify the organization(s) that approved or provided guidance on the study protocol, OR state that no ethical approval or guidance was required and explain why not.*

Note that full information on the approval of the study protocol must also be provided in the manuscript.

Animals and other research organisms

Policy information about studies involving animals; ARRIVE guidelines recommended for reporting animal research, and Sex and Gender in Research

Laboratory animals	All animal work was conducted in compliance with the relevant institutional and national regulations governing the use of animals in research. Experiments performed in the United Kingdom were authorized by the UK Home Office under Project License 70/8453. Experiments performed at the California Institute of Technology were approved by the Institutional Animal Care and Use Committee under protocol 1874.
Wild animals	N/A
Reporting on sex	The mESC line used in this study was male. MEFs were prepared from E12.0 embryos, with sex determined after establishment of the cell lines. Both male and female MEF lines were included. Sex was not expected to influence the centrosome, proliferation, or autophagy-related assays performed in this study, and no sex-specific analyses were conducted.
Field-collected samples	N/A
Ethics oversight	All animal work was performed in accordance with the approved UK Home Office Project Licence 70/8453 and Caltech IACUC protocol 1874.

Note that full information on the approval of the study protocol must also be provided in the manuscript.

Clinical data

Policy information about clinical studies

All manuscripts should comply with the ICMJE guidelines for publication of clinical research and a completed CONSORT checklist must be included with all submissions.

Clinical trial registration	N/A
Study protocol	N/A
Data collection	N/A
Outcomes	N/A

Dual use research of concern

Policy information about dual use research of concern

Hazards

Could the accidental, deliberate or reckless misuse of agents or technologies generated in the work, or the application of information presented in the manuscript, pose a threat to:

No	Yes	
☒	☐	Public health
☒	☐	National security
☒	☐	Crops and/or livestock
☒	☐	Ecosystems
☒	☐	Any other significant area

Experiments of concern

Does the work involve any of these experiments of concern:

No	Yes	
☒	☐	Demonstrate how to render a vaccine ineffective
☒	☐	Confer resistance to therapeutically useful antibiotics or antiviral agents
☒	☐	Enhance the virulence of a pathogen or render a nonpathogen virulent
☒	☐	Increase transmissibility of a pathogen
☒	☐	Alter the host range of a pathogen
☒	☐	Enable evasion of diagnostic/detection modalities
☒	☐	Enable the weaponization of a biological agent or toxin
☒	☐	Any other potentially harmful combination of experiments and agents

Plants

Seed stocks	*Report on the source of all seed stocks or other plant material used. If applicable, state the seed stock centre and catalogue number. If plant specimens were collected from the field, describe the collection location, date and sampling procedures.*
Novel plant genotypes	*Describe the methods by which all novel plant genotypes were produced. This includes those generated by transgenic approaches, gene editing, chemical/radiation-based mutagenesis and hybridization. For transgenic lines, describe the transformation method, the number of independent lines analyzed and the generation upon which experiments were performed. For gene-edited lines, describe the editor used, the endogenous sequence targeted for editing, the targeting guide RNA sequence (if applicable) and how the editor was applied.*
Authentication	*Describe any authentication procedures for each seed stock used or novel genotype generated. Describe any experiments used to assess the effect of a mutation and, where applicable, how potential secondary effects (e.g. second site T-DNA insertions, mosiacism, off-target gene editing) were examined.*

ChIP-seq

Data deposition

☐ Confirm that both raw and final processed data have been deposited in a public database such as GEO.

☐ Confirm that you have deposited or provided access to graph files (e.g. BED files) for the called peaks.

Data access links<br>*May remain private before publication.*	*For "Initial submission" or "Revised version" documents, provide reviewer access links. For your "Final submission" document, provide a link to the deposited data.*
Files in database submission	*Provide a list of all files available in the database submission.*
Genome browser session<br>(e.g. UCSC)	*Provide a link to an anonymized genome browser session for "Initial submission" and "Revised version" documents only, to enable peer review. Write "no longer applicable" for "Final submission" documents.*

Methodology

Replicates	*Describe the experimental replicates, specifying number, type and replicate agreement.*
Sequencing depth	*Describe the sequencing depth for each experiment, providing the total number of reads, uniquely mapped reads, length of reads and whether they were paired- or single-end.*
Antibodies	*Describe the antibodies used for the ChIP-seq experiments; as applicable, provide supplier name, catalog number, clone name, and lot number.*
Peak calling parameters	*Specify the command line program and parameters used for read mapping and peak calling, including the ChIP, control and index files used.*
Data quality	*Describe the methods used to ensure data quality in full detail, including how many peaks are at FDR 5% and above 5-fold enrichment.*
Software	*Describe the software used to collect and analyze the ChIP-seq data. For custom code that has been deposited into a community repository, provide accession details.*

Flow Cytometry

Plots

Confirm that:

- ☐ The axis labels state the marker and fluorochrome used (e.g. CD4-FITC).
- ☐ The axis scales are clearly visible. Include numbers along axes only for bottom left plot of group (a 'group' is an analysis of identical markers).
- ☐ All plots are contour plots with outliers or pseudocolor plots.
- ☐ A numerical value for number of cells or percentage (with statistics) is provided.

Methodology

Sample preparation	N/A
Instrument	N/A
Software	N/A
Cell population abundance	N/A
Gating strategy	N/A

☐ Tick this box to confirm that a figure exemplifying the gating strategy is provided in the Supplementary Information.

Magnetic resonance imaging

Experimental design

Design type	N/A
Design specifications	N/A
Behavioral performance measures	N/A

Acquisition

Imaging type(s)	N/A
Field strength	N/A
Sequence & imaging parameters	N/A
Area of acquisition	*State whether a whole brain scan was used OR define the area of acquisition, describing how the region was determined.*

Diffusion MRI ☐ Used ☒ Not used

Preprocessing

Preprocessing software	N/A
Normalization	N/A
Normalization template	N/A
Noise and artifact removal	N/A
Volume censoring	N/A

Statistical modeling & inference

Model type and settings	N/A
Effect(s) tested	N/A

Specify type of analysis: ☐ Whole brain ☐ ROI-based ☐ Both

Statistic type for inference<br>(See Eklund et al. 2016)	N/A
Correction	N/A

Models & analysis

n/a	Involved in the study
☒	☐ Functional and/or effective connectivity
☒	☐ Graph analysis
☒	☐ Multivariate modeling or predictive analysis
