# Supplementary material for: Sensing centrosome amplification: the interface between centriole duplication and autophagy
Source: Nat Commun. 2026 Jun 21;17:7815. doi: 10.1038/s41467-026-74702-9 (PMC13439419; doi:10.1038/s41467-026-74702-9)
Supplement: Supplementary file 2 [file 41467_2026_74702_MOESM16_ESM.pdf]

nature portfolio

Peer Review File

Sensing Centrosome Amplification: the Interface between Centriole Duplication and Autophagy

Corresponding Author: Dr Paula Almeida Coelho

This file contains all reviewer reports in order by version, followed by all author rebuttals in order by version.

Version 0:

Reviewer comments:

Reviewer #1

(Remarks to the Author)
Nat Comm Revision

Coelho and co-authors performed an elegant CRISPR-Cas9 screen to identify new proteins/pathways that allow overcoming cell proliferation blockage imposed by centrosome amplification. While confirming the relevance of pathways already known to be involved in this process, the authors also shed light on a novel role for the Guanine-nucleotide Activating Protein ARHGAP15 and autophagy. This study is highly relevant to the understanding of the mechanism(s) underlying cancer cell capability to escape cell proliferation arrest in case of centrosome duplication. Also, it adds new potential mechanistic insights to the intriguing relationship between autophagy and mitosis/cell proliferation regulation. However, this manuscript suffers major flaws affecting both the technical and rationale aspects of the study. As a result, several conclusions are not fully supported by the data, while autophagy is not properly assessed in several experiments, as well as not being fully explored in terms of autophagy pathways involved. In addition, the molecular mechanism(s) underlying ARHGAP15-mediated regulation of autophagy and autophagy-mediated regulation of cell cycle upon centrosome duplication is not clear. Major and minor concerns regarding the study are as reported below.

Major comments

1. The statistical analysis of the experiments is missing. A proper assessment of the statistical significance should be included in each experiment, and the relevant related information reported in the figure legends and methods.

2. Protein levels of the proteins knock-down and/or over-expressed should be checked in each experiment, or at least the first time the knock-down/over-expression is shown.

3. ULK1-mediated phosphorylation of ATG16L1 at S278 (p-ATG16L1) drives ATG16L1 localization to the site of bacteria internalization during xenophagy (PMID: 31267703). Since the authors find this phosphorylation event to be relevant for cell cycle regulation upon centrosome duplication, it would be interesting to assess whether p-ATG16L1 localizes at amplified centrosomes. Also, is this phosphorylation triggered upon PLK4 over-expression (i.e. centrosome amplification). Does ULK1 inhibition affect ATG16L1 role in autophagy-mediated regulation upon centrosome amplification?

4. Fig. 2D and 5A. Co-immunoprecipitation (Co-IP) experiments miss the proper controls (i.e., total extracts/inputs run on the same gel of the Co-IP samples, as well as a negative control for the Co-IP samples). Also, the bands corresponding to Flag-ARHGAP15 have a different appearance in the western blots in 2D and 5A, and the same applies to ATG16L1 bands in the same figures. Since the Co-IPs are performed in the same cell line, and with the same Flag-ARHGAP15 construct, this difference in the band appearance is unexpected. Also, the interaction between ARHGAP15 should be also shown in endogenous conditions, to further strengthen the result.

5. Fig. 3F, 4G, 5I, 6E. The biochemical analysis of LC3B per se is not informative in terms of autophagy levels. An autophagy flux assay has to be performed instead, by using lysosomal inhibitors to measure the degradation rate of LC3BII (PMID: 33634751). Densitometry of LC3BII levels from independent experiments, each normalized on the relative loading control, should be shown, and statistically analysed.

6. It is not clear why the authors conclude that ARHGAP15 affects autophagy through its interaction with ATG16L1. Is the interaction modulated upon Plk4 over-expression, which is when ATG16L1 is reported be responsible to regulate cell proliferation in response to centrosome amplification? In this reviewer´s view, ATG16L1 effect on ARHGAP15-mediated regulation of centrosome amplification may be mediated by a general role of ATG16L1 in autophagy, rather than by its interaction with ARHGAP15. In line with this hypothesis, general autophagy inhibition through autophagy drugs recapitulates siATG16L1 effect (Fig. 3I). Is this also the case for knock-down experiments of autophagy genes other than ATG16L1? Also, the authors should better characterize the ARHGAP15-ATG16L1 interaction. What ATG16L1 domain is relevant to the interaction? Is the interaction affected by ULK1-mediated phosphorylation (a quantification of at least 3 independent experiments as in Extended Fig. 4 should be quantified)?

7. What is the fate of the cells undergoing centrosome amplification and ATG16L1 knock-down/autophagy inhibition? Do they die? Do they undergo mitotic catastrophe? Do they accumulate genomic aberrations? Is cell proliferation affected? Phenotypes other than centrosome amplification should be monitored.

8. TIAM1 role in autophagy regulation upon centrosome amplification is brought up very late in the manuscript, and it has been barely validated, but not characterized. Therefore, it does not add to the molecular mechanism underlying the cross-talk between autophagy and centrosome amplification. I would leave it out of this manuscript.

9. It is not clear whether the authors hypothesize that autophagy selectively targets centrosomes resulting from centrosome duplication. This should be further clarified and properly tested/investigated. Also, is selective autophagy involved? Are selective autophagy regulators (e.g. selective autophagy receptors/adaptors, “eat me” signals, etc.) involved in the autophagy-mediate escape of cell proliferation blockage upon centrosome amplification?

10. Autophagy has been reported to degrade centriolar satellites (CS) to maintain centrosome homeostasis (ref. 86 in this manuscript). Are CS involved in the regulation of centrosome amplification/amplified centrosome cleareance through autophagy?

Minor comments
1. Line 44. “Which is by activated” is probably “Which is activated by”

2. The authors report on an unknown role of PCM1 in autophagy. This is not accurate: Sharon Tooze´s laboratory reported a role for PCM1 in regulating autophagy, through its interaction with GABARAP/L2. Please revise the manuscript discussion accordingly.

Reviewer #2

(Remarks to the Author)
Centrosome amplification has long been described as a feature of human tumors. Despite this well-established association, centrosome amplification can impose negative proliferative effects on cell proliferation. To overcome some of these inhibitory effects, different genome-wide screens have identified the PIDDosome as an inhibitor of cells with extra centrosomes by inhibiting cell cycle via p53 stabilization. However, this complex is not activated in all cell types, and it is likely that other pathways contribute for the proliferation of cells carrying extra centrosomes.

Here, the authors use a CRISPR/Cas9 screening strategy to identify new genes/pathways that allow cells with extra centrosomes to proliferate. They found several genes, whose KO are enriched in cells overexpressing PLK4. Some of the genes are involved in autophagy regulation, such as ARHGAP15. The authors further show that the autophagy protein ATG16L1 is required for centrosome reduction downstream of ARHGAP15 loss and that ARFGAP15 is counteracted by ARHGEF2. The authors propose that activation of autophagy by centriole satellites can prevent centrosome amplification. While overall this work provides a new dimension to how the negative impact of centrosome amplification can be are overcome, there are several issues with how the data is presented and some conclusions lack strong evidence. Clarity and general data presentation could be improved to help readability. There are major issues that should be addressed prior to publication, which are outlined below.

Major concerns:
1. In general, the description of how the screen was conducted is not well explained. It is not clear what was the control used for the screen, were cells without DOX used as control? Or cells treated with DOX but not Cas9? Scheme of Extended figure 1 does not contain this crucial information. This is important because some of the enriched KO could have a general positive impact of cell proliferation. This is a major concern as it is not clear that the treatments/depletions are specific to cells with amplified centrosomes.

2. The authors should provide data on induction of centrosome amplification in the screen setting and what happens to centrosome amplification over time? It is known that, upon induction, extra centrosomes are poorly maintained in tissue culture.

3. Related with the comment above, the authors often state ‘centrosome reduction’ to describe low centrosome numbers. However it is unclear what this means. Are the treatments used reducing centrosomes (for example once these are amplified)? Or do they block centrosome duplication and hence cells will have less centrosomes? This is not the same and should be clarified.

4. The authors should explain why DOX was kept throughout the duration of the screen. Constitutive overexpression of PLK4 can have toxic effects and it needs to be shown whether the loss of viability shown in extended figure a (scheme) is a consequence of centrosome amplification or toxic PLK4 overexpression. If PLK4 is only transiently overexpressed in mESCs, do the cells have the same proliferative defects?

5. A major issue is also data presentation. The graphs used to plot centrosome amplification are not intuitive (e.g. fig1c). What are the different 'little' bars? Individual cells? Experiments? How many experiments were done and how many cells were quantified in each plot? Also, no stats are shown in any graphs, and thus the significance of the data is not always clear.

6. On line 133 the authors state that proliferation recovery was measured in the MEFs. However, the figure the authors mention (Fig 1b) only refers to mESCs. A validation of proliferation data in MEFs to connect the work to the initial data should be provided as this is mentioned in the abstract as well.

7. The phenotypic categories of the different knockdowns seem somehow artificial. For example, category III is not based on phenotype but rather on the known function of these proteins. Lats2 depletion leads to increase in >2 mothers, while depletion of p53 and Pidd1 doesn't. Also unclear how this information is used in the rest of the manuscript, for example when autophagy is inhibited.

8. If the authors want to propose that RAC1 activity is crucial in reducing extra centrosomes, then a more direct experiment should be used to test this idea. For example, inhibiting RAC1 in cells depleted of ARHGAP15 KO to analyze centrosome number could be used. As it stands, link to RAC1 is weak despite being described in the abstract.

9. All pull-down/IP experiments need controls (e.g control for FLAG non-specific binding in Figure 2d) and WB for whole cell lysates.

10. The authors propose that inhibition of the autophagy pathways leads to centrosome amplification even in control cells (figure 3h). Can the authors demonstrate that these inhibitors do not cause cytokinesis failure, which would lead to increased centrosome numbers? Images provided on this figure are not great, microtubules not easy to see for all images and centrosome channels should be separated for better visualization.

11. The data describing phagophore-like vesicles in figure 4f seems irrelevant or its relevance is not well explained.

12. The authors should test if depletion of ARHGEF2 compromises cytokinesis, which would also lead to extra centrosomes.

13. Images provided on fig 5b are not very clear. Centrosome channels need to be separated to enable proper visualization.

14. Authors state that LC3B vesicles co-localise with CEP170 and accumulate on autophagosomes after PLK4 overexpression. However, there is no quantification for these data and since not all cells will have extra centrosomes (even after DOX), the authors should analyses cells with and without amplified centrosomes in the +DOX condition and determine if the association of autophagosomes with hCEP170 depends on centrosome amplification. This will help understading the specificity of the phenotypes to cells with extra centrosomes.

15. The graphs in figure 6i are not intuitive and difficult to follow.

Reviewer #3

(Remarks to the Author)
Through genome-wide screens, the Glover lab identified the RAC1-specific GTPase-activating protein ARHGAP15 as a novel factor permitting centrosome amplification induced by PLK4 overexpression. To understand how loss of ARHGAP15 suppresses centrosome amplification, the authors found that the autophagy protein ATG16L1 is an interacting partner of ARHGAP15, that ARHGAP15 is a negative regulator of ATG16L1-mediated autophagy, and that ATG16L1-mediated autophagic fluxes are required for centrosome reduction induced by loss of ARHGAP15. Moreover, the authors identified ARHGEF2 as the guanine-nucleotide exchange-factor (GEF) counteracting ARHGAP15, which activates RAC1 and autophagy to reduce centrosome numbers. Furthermore, to understand how supernumerary centrosomes are linked to autophagy activation, the authors found that the centrosomal protein CEP170 is in complexes with ARHGEF2, that CEP170 disassociates from amplified centrosomes, and that CEP170 depletion induces autophagy reduction while causing/enhancing centrosome amplification specifically in the absence of PLK4 overexpression, consistent with an idea that CEP170 triggers ARHGEF2-mediated autophagy to regulate centrosome numbers. Together, the authors suggest that autophagy is a key regulatory module of centriole duplication.

The overall result is important and should be published as it uncovers several previously unrecognized points including:
a). Autophagy ensures proper centriole duplication.
b). "CEP170 -> ARHGEF2/ARHGAP15 -> RAC1 -> ATG16L1" connects centriole duplication to autophagy.
c). Uncovering a direct role of RAC1 (together with its associated GEF/GAP) in regulating autophagy adds additional angles through which RAC1 alterations can impact a variety of diseases.

Please address, clarify, or discuss the following points as they appear in the result, which I believe can be helpful for readers:

1. The centrosome amplification phenotype, which was described as “CEP152 double dots”, is not very clear to me. The authors marked two areas of mitotic cells with centrosome amplification to show “CEP152 double dots”, but more often than not, I could not tell if they are double dots or clusters. If centrosomes were amplified in these cells, why select only two areas (two poles?)? If extra centrosomes are randomly clustered to the two spindle poles, why specifically two centrosomes per pole (double dots)?

2. Why does expressing ARHGAP15-R317M in the absence of wild-type ARHGAP15 lead to significantly more cells (~16%) that lose their centrosomes, and even more so (25-30%) when PLK4 is overexpressed? Similarly, why does knocking down endogenous ARHGAP15 alone did not cause cells to lose centrosomes when PLK4 is not overexpressed? Does overexpressing ATG16L1 cause centrosome loss?

3. If autophagy ensures proper centriole duplication, why does ATG16L1 depletion not cause centrosome amplification as a default (without PLK4 overexpression), given that the autophagy pathway is off? Why does it depend on PLK4 overexpression? Perhaps ATG16L1 depletion is incomplete?

4. I think in Fig. 4f, the CEP152 and EGFP panels were swapped.

5. Does ARHGEF2 co-localize with CEP170 at the appendage of mother centrioles? If so, does the loss of CEP170 upon centrosome amplification trigger the loss of ARHGEF2 from mother centrioles as well?

6. Why does the enhancement of centrosome amplification by CEP170 depletion seen only when PLK4 is not overexpressed? Could it mean that there are other centriolar components mediating the condition under PLK4 overexpression?

Reviewer #4

(Remarks to the Author)
I co-reviewed this manuscript with one of the reviewers who provided the listed reports. This is part of the Nature Communications initiative to facilitate training in peer review and to provide appropriate recognition for Early Career Researchers who co-review manuscripts.

Version 1:

Reviewer comments:

Reviewer #2

(Remarks to the Author)
In the revised version of their manuscript, the authors addressed several of the issues raised during the revision, which include the addition of new data to support their findings. There are still some concerns regarding the clarity of data presentation/description, and it will be up to the authors to decide how they want their work to be perceived and understood. Below, are the original points raised that have not been fully addressed.

1. While we understand the gene groupings decided by the authors take into account centrosome number and proliferation, there is no data to show that some of these genes do not impact proliferation in control -DOX cells. Hence, claims of the specificity of proliferation recovery for cells with amplified centrosomes cannot be made. This should be either addressed with data or by ensuring that the text states that the authors cannot exclude that impacts on proliferation may be independent of centrosome number (even if centrosome numbers are reduced).

2. We appreciate the attempt to demonstrate centrosome amplification. However, as it is known that the amplification is lost in vitro over time, the authors should quantify the levels of centrosome amplification after 4ug/ml DOX treatment, and again after 9 days as asked in the initial revision. The images do not provide quantitative data, which I am sure the authors would agree.

4. While the constitutive Plk4 expression may not impact embryonic viability, this does not account for proliferative defects and cytotoxic effects within the cells. This is what the original question was to address. At the present, the authors cannot exclude that within a heterogenous population of cells, PLK4 constitute OE has no cytotoxic effect. The authors could test this by comparing PLK4 transient OE. Furthermore, as no data is provided to the extent of centrosome amplification relative to extended figure 1a (viability data) relative to the concentration of DOX, it is impossible to tell if the loss of viability is associated with levels of centrosome amplification or PLK4 OE.

5. In general, data should be presented per experiment, and it should be clear which fields correspond with each experiment. The current representation of the data does not allow the reader to assess variability between experiments, which is an issue. We appreciate that the authors added statistics to some of the graphs, although not to all. Where stats have been added, it is difficult to assess what is being compared and what is significant. Furthermore, whilst we appreciate that stats analysis can clutter figures, it is important to have the information available in the figure rather than in statistics

tables. It is puzzling that this is not done.

10 and 12. The authors state that they paid careful attention to DNA content. Please could the authors provide evidence of this, and that the cells are not undergoing cytokinesis failure? At least ARHGEF2 has known roles in cytokinesis: https://pubmed.ncbi.nlm.nih.gov/17488622/

14. According to the authors model in figure 6l, increased autophagy of centrosome components in the absence of ARHGAP15, leads to loss of amplified centrosomes in cells treated with DOX. This is also supported by their data in figure 1c where loss of ARHGAP15 in control (-DOX) cells has no impact on normal centrosome number. Thus, according to the authors this is specific for cells with extra centrosomes, otherwise the expectation would be that ARHGAP15 loss drives centrosome loss in normal cells as well. Thus, what was asked to the authors, maybe not as clearly as it should, was to test that co-localization of LC3B and CEP170 puncta in ARHGAP15 depleted +DOX cells with and without amplified centrosomes. Only a fraction of these cells has centrosome amplification, and therefore this distinction is important to test their model.

Reviewer #3

(Remarks to the Author)
This is an important study revealing the number control of centrosome assembly by autophagy through a molecular pathway involving CEP170-ARHGEF2/ARHGAP15-RAC1-ATG16L1. While I fully support its publication, the complete centrosome loss phenotype induced by the R317M mutant of ARHGAP15 even when PLK4 is elevated (see Figure 2b and Extended Figure 2b) is unusual/unexpected with regard to the current understanding of centrosome assembly or other data provided in this study. I think it should be pointed out and discussed.

Reviewer #4

(Remarks to the Author)
I co-reviewed this manuscript with one of the reviewers who provided the listed reports. This is part of the Nature Communications initiative to facilitate training in peer review and to provide appropriate recognition for Early Career Researchers who co-review manuscripts.

Version 2:

Reviewer comments:

Reviewer #2

(Remarks to the Author)
Thanks to the authors for their reply. I would like to stress that ALL the points in the previous revision letter were relative to the initial revision and not addressed, in my opinion, satisfactory. The authors prefer to continue to address issues raised with changes in the text as opposed to add data when appropriate even if it exists (or should exist according to them). Thus, I will not have any further comments on this manuscript and leave to the authors to decide how they want their work to be displayed, as they know best what works for them.

Reviewer #3

(Remarks to the Author)
The authors have addressed my concerns, and I therefore support the publication.

Reviewer #4

(Remarks to the Author)
I co-reviewed this manuscript with one of the reviewers who provided the listed reports. This is part of the Nature Communications initiative to facilitate training in peer review and to provide appropriate recognition for Early Career Researchers who co-review manuscripts.

Open Access This Peer Review File is licensed under a Creative Commons Attribution 4.0 International License, which permits use, sharing, adaptation, distribution and reproduction in any medium or format, as long as you give appropriate credit to the original author(s) and the source, provide a link to the Creative Commons license, and indicate if changes were made.
In cases where reviewers are anonymous, credit should be given to 'Anonymous Referee' and the source.
The images or other third party material in this Peer Review File are included in the article's Creative Commons license, unless indicated otherwise in a credit line to the material. If material is not included in the article's Creative Commons license and your intended use is not permitted by statutory regulation or exceeds the permitted use, you will need to obtain permission directly from the copyright holder.
To view a copy of this license, visit https://creativecommons.org/licenses/by/4.0/

Reviewer #1 (Remarks to the Author):

Nat Comm Revision
The excel and co-authors performed an elegant CRISPR-Cas9 screen to identify new proteins/pathways that allow overcoming cell proliferation blockage imposed by centrosome amplification. While confirming the relevance of pathways already known to be involved in this process, the authors also shed light on a novel role for the Guanine-nucleotide Activating Protein ARHGAP15 and autophagy. This study is highly relevant to the understanding of the mechanism(s) underlying cancer cell capability to escape cell proliferation arrest in case of centrosome duplication. Also, it adds new potential mechanistic insights to the intriguing relationship between autophagy and mitosis/cell proliferation regulation. However, this manuscript suffers major flaws affecting both the technical and rationale aspects of the study. As a result, several conclusions are not fully supported by the data, while autophagy is not properly assessed in several experiments, as well as not being fully explored in terms of autophagy pathways involved. In addition, the molecular mechanism(s) underlying ARHGAP15-mediated regulation of autophagy and autophagy-mediated regulation of cell cycle upon centrosome duplication is not clear. Major and minor concerns regarding the study are as reported below.

We thank the referee for stating that our work represents an "elegant screen". We believe we have identified the core pathway of how the cell responds to supernumerary centrosomes through autophagy and thank the referee for pointing out the parts of the paper that need to be strengthened to support our study.

Major comments

1. The statistical analysis of the experiments is missing. A proper assessment of the statistical significance should be included in each experiment, and the relevant related information reported in the figure legends and methods

To address the referee's concerns, we now present all original data together with the corresponding statistical analyses for each individual figure in as complete form as is possible in the revised Excel files (Extended Tables 1–8). Each file contains a tab entitled Raw data for the respective figure, followed by an additional tab presenting the statistical analysis using non-parametric methods. The statistical method applied for each dataset is indicated, and the individual values plotted in the graphs are provided in detail for every condition. The description of statistical methods is also included in the Materials and Methods section.

All data represent at least three independent experiments, in which more than 200 cells were analyzed per condition. Graphs display the mean, with a black bar representing the mean ± 95% confidence interval (CI). Non-parametric statistical methods were used, as the assumptions of normal distribution and equal variance could not be ensured. Differences across multiple groups were first assessed using the Kruskal–Wallis test. When the overall test was significant, Dunn's multiple comparisons test was applied as a post hoc analysis to identify specific group differences, with adjustment for multiple pairwise comparisons. All analyses were performed using GraphPad Prism (version 10.3.0). This is indicated in the Methods section.

2. Protein levels of the proteins knock-down and/or over-expressed should be checked in each experiment, or at least the first time the knock-down/over-expression is shown.

We thank the reviewer for this important point. Antibodies are not available for all of the gene products that were depleted. Where antibodies were available, we now include Western blot data to confirm protein knockdown or overexpression. In cases where antibodies were not available, qRT–PCR was used to assess mRNA depletion (Supp. Fig. 1g–j, Extended Data Figure 4, Extended Tables 2, 4-8).

The phenotypes were always quantified and compared in the presence and absence of the respective endogenous proteins. In these cases, a dominant effect of the phenotype was observed after siRNA depletion of mouse Arhgap15 or Atg16l1, indicating that the overexpression phenotype does not confound the interpretation. Expression levels were further assessed by immunofluorescence and Western blot, where possible.

3. ULK1-mediated phosphorylation of ATG16L1 at S278 (p-ATG16L1) drives ATG16L1 localization to the site of bacteria internalization during xenophagy (PMID: 31267703). Since the authors find this phosphorylation event to be relevant for cell cycle regulation upon centrosome duplication, it would be interesting to assess whether p-ATG16L1 localizes at amplified centrosomes. Also, is this phosphorylation triggered upon PLK4 over-expression (i.e. centrosome amplification). Does ULK1 inhibition affect ATG16L1 role in autophagy-mediated regulation upon centrosome amplification?

We thank the reviewer for raising this important question. We previously attempted to address this question using anti–phospho-ATG16L1 antibodies from two different sources. Unfortunately, these antibodies produced

inconsistent results and lacked reproducibility in both Western blot and immunofluorescence analyses. This interesting question will therefore be the topic of future studies. The question of whether ULK1 inhibition affects ATG16L1 role in autophagy-mediated regulation upon centrosome amplification is largely addressed by our finding that the consequences of depletion of either ULK1 or ATG16L1 are very similar in leading to centrosome amplification.

4. Fig. 2D and 5A. Co-immunoprecipitation (Co-IP) experiments miss the proper controls (i.e., total extracts/inputs run on the same gel of the Co-IP samples, as well as a negative control for the Co-IP samples). Also, the bands corresponding to Flag- ARHGAP15 have a different appearance in the western blots in 2D and 5A, and the same applies to ATG16L1 bands in the same figures. Since the Co-IPs are performed in the same cell line, and with the same Flag-ARHGAP15 construct, this difference in the band appearance is unexpected. Also, the interaction between ARHGAP15 should be also shown in endogenous conditions, to further strengthen the result.

We appreciate the reviewer's comments. We have repeated the experiment with an EGFP-Flag control and now include inputs and IPs in the revised figure. We have expanded our analysis of the Arhgap15–ATG16L1 interaction to include additional Arhgap15 mutants as well as an internal control (EGFP-Flag), all expressed in the same cell lines. Input controls showing the expression levels of the relevant proteins are now included in Figure 2D and 5A. Our results demonstrate that the interaction between Arhgap15 and ATG16L1 requires the Pleckstrin Homology (PH) domain of Arhgap15, which is known to mediate interactions with key regulatory proteins and influence subcellular localization. Furthermore, we find that this interaction is also dependent on the GTPase-activating (GAP) function of Arhgap15.

New Figure 2D

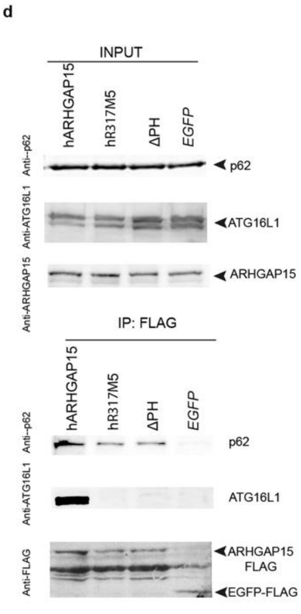

New Figure 5A

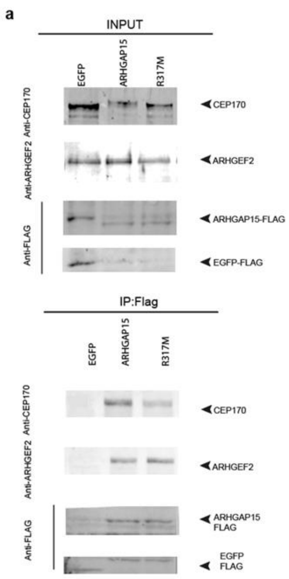

The figure legend is revised to indicate that all immunoprecipitation (IP) experiments were performed using embryonic fibroblasts expressing the indicated Arhgap15 variants. The IP experiments were repeated with the same Antibody. The revised figure legend reads for Fig2 **"**(d**)** Extracts of Plk4OE MEFs transfected with FLAG-tagged wild-type ARHGAP15, the R317M GAP-deficient mutant, the ΔPH mutant lacking the Pleckstrin Homology (PH) domain, or EGFP-FLAG control, as indicated. Total cell extracts (input) and anti-FLAG immunoprecipitates were analyzed by Western blotting. ARHGAP15-FLAG expression served as loading control and is observed with Anti-ARHGAP15 or anti-Flag antibodies in input or pull downs respectively. ATG16L1 and p62/SQSTM1 were detected in inputs and pulldowns using specific antibodies. Pulldown experiments were performed in three independent replicates.". *The revised figure legend for Fig5 "***(a)** *Tet^{ON}-Plk4* MEFs were transfected with FLAG-tagged wild type h*Arhgap15*, h*Arhgap15* R317M (hR317M) or EGFP-FLAG control, as

indicated. Endogenous mouse *Arhgap15* was knocked down using siRNA. Extracts were then subjected to anti-FLAG purification, followed by Western blotting analysis. ARHGAP15 provides loading control. ARHGAP15 co-precipitates with ARHGEF2, and centriolar protein CEP170."

The text of the Results is modified to read "However, in cells transfected with either the R317M mutant (deficient in GAP activity) or the ΔPH mutant lacking the Pleckstrin Homology (PH) domain, ATG16L1 was not detected in the pulldown. These results demonstrate that the ARHGAP15–ATG16L1 interaction requires both the GAP activity and the PH domain of ARHGAP15, the latter being critical for binding regulatory proteins and influencing subcellular localization. We also detected p62/SQSTM1 in the pulldown suggests that this adaptor protein may potentially connect ARHGAP15 to selective autophagy pathways." *Future studies will be needed to confirm this and to identify additional interacting partners (Fig. 2d).*

5. Fig. 3F, 4G, 5I, 6E. The biochemical analysis of LC3B per se is not informative in terms of autophagy levels. An autophagy flux assay has to be performed instead, by using lysosomal inhibitors to measure the degradation rate of LC3BII (PMID: 33634751). Densitometry of LC3BII levels from independent experiments, each normalized on the relative loading control, should be shown, and statistically analysed

We fully acknowledge the reviewer's concern that LC3B-II levels alone are not sufficient to assess autophagic activity. Indeed, autophagic flux measurements are required to evaluate the degradation dynamics of LC3B-II. In response, we performed autophagy flux assays using the lysosomal inhibitor Bafilomycin A1, as previously described. Densitometric analysis of LC3B-II levels was carried out across independent biological replicates, with each sample normalized to its corresponding loading control.

Densitometric quantification of LC3B-II normalized to loading controls is now included in the revised manuscript (Fig. 3f, Extended Fig. 3a; Figs. 4h–i, 5h–i, 6e–f). The quantified data and statistical analyses are provided in the Extended Data Tables, confirming altered autophagic flux under the indicated conditions.

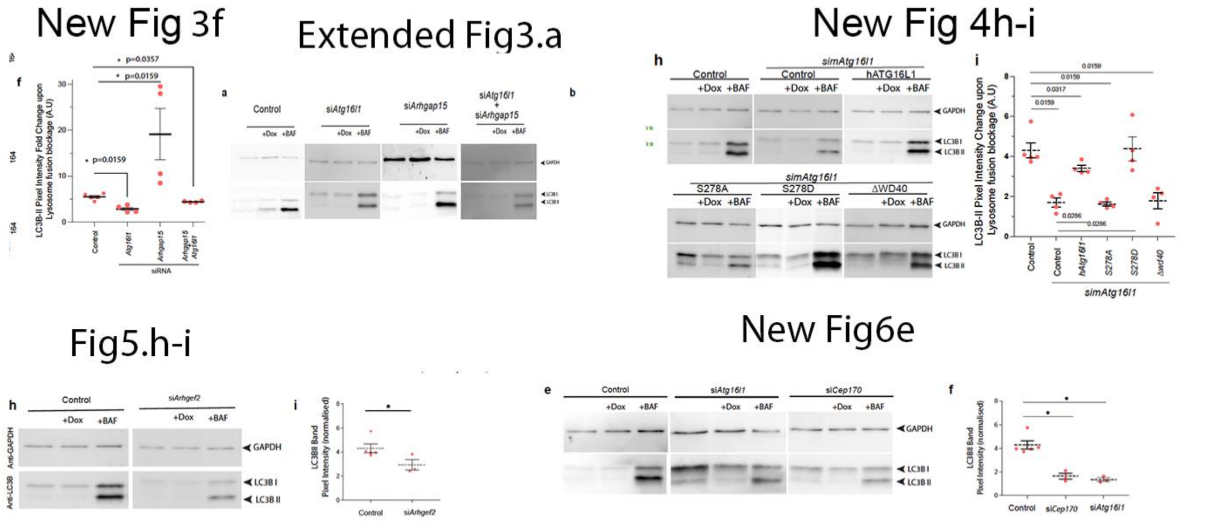

*The figure legends were revised and read: "*Fig3.f Quantification of LC3B-II, the lipidated form of a phagosome associated ATG8 protein, in Western blot analysis to measure autophagic flux in TetON-Plk4 MEFs cultured without or with doxycycline (+Dox) in the presence or absence of bafilomycin A1 and subjected to RNAi using the indicated siRNAs. LC3B-II levels were quantified by densitometry, normalized to GAPDH and expressed as mean ± SEM from three independent experiments (n = 3). *p < 0.05. Western blots are shown in Extended. Fig. 3a".
The accompanying Ext. Fig3 reads: "(a) Autophagic flux was assessed by monitoring LC3B-II levels after siRNA-mediated depletion of Arhgap15, Atg16l1, or both, compared to control MEFs, in the presence or absence of doxycycline (+Dox). Cells were also treated with bafilomycin A1 (16 h incubation, an inhibitor of autophagosome–lysosome fusion, added 80 h after initiation of siRNA knockdown). Loss of Arhgap15 expression resulted in increased LC3B-II levels relative to control, indicating that ARHGAP15 is required to restrain autophagy. LC3B-II was quantified by densitometry of Western blots and normalized to GAPDH. The corresponding quantification is shown in Fig. 3f, with complete data and statistical analyses provided in Extended Data Table 5".

The corrected figure for fig4 (h-I) reads:" **(h)** *Analysis of autophagic flux based on LC3B-II (phagophore/autophagosome-associated) and LC3B-I (cytosolic) levels in Tet^{ON}-Plk4 MEFs depleted of endogenous ATG16L1 and expressing the indicated hATG16L1 variants, cultured without or with doxycycline (+Dox) to induce PLK4 expression. Cells were analyzed in the presence or absence of bafilomycin A1 (BAF).* ***(i)*** LC3B-II levels were quantified by densitometry and normalized to the density of the GAPDH band. Fold changes between each sample and the corresponding BAF-treated control are shown and expressed as mean ± SEM from three independent experiments (n = 3). Statistical significance was determined, with $p < 0.05$ (see Extended Data Table 6)."

Also, the updated Fig 5 (h-i) now reads "Fig5*(h,i) Analysis of autophagic flux by western blot, showing LC3B-II levels in extracts from MEFs after Arhgef2 siRNA knockdown, in the presence or absence of bafilomycin A1, normalized to GAPDH.* Fold changes between each sample and the corresponding BAF-treated control are shown and expressed as mean ± SEM from three independent experiments (n = 3). Statistical significance was determined, with $^{*}p < 0.05$ (see Extended Data Table 7)."

And the updated legend for Fig.6e now reads "Autophagic flux was analyzed based on levels of phagophore/autophagosome-associated LC3B-II in extracts from *Tet^{ON}-Plk4* MEFs: control cells, cells depleted of endogenous ATG16L1, or cells depleted of endogenous CEP170. Cells were analyzed in the absence or presence of bafilomycin A1, and LC3B-II band intensity was quantified by densitometry, normalized to GAPDH. *Fold changes between each sample and the corresponding BAF-treated control are shown and expressed as mean ± SEM from three independent experiments (n = 3). Statistical significance was determined, with $^{*}p < 0.05$ (see Extended Data Table 8)."*

6. It is not clear why the authors conclude that ARHGAP15 affects autophagy through its interaction with ATG16L1. Is the interaction modulated upon Plk4 over-expression, which is when ATG16L1 is reported be responsible to regulate cell proliferation in response to centrosome amplification? In this reviewer´s view, ATG16L1 effect on ARHGAP15-mediated regulation of centrosome amplification may be mediated by a general role of ATG16L1 in autophagy, rather than by its interaction with ARHGAP15. In line with this hypothesis, general autophagy inhibition through autophagy drugs recapitulates siATG16L1 effect (Fig. 3I). Is this also the case for knock-down experiments of autophagy genes other than ATG16L1? Comment Also, the authors should better characterize the ARHGAP15-ATG16L1 interaction. What ATG16L1 domain is relevant to the interaction?

We thank the reviewer for raising this important point. We agree that the primary function of ATG16L1 in this context is likely related to its well-established role in autophagy, which is essential for maintaining centrosome homeostasis. Importantly, our data suggest that the interaction between ARHGAP15 and ATG16L1 is inhibitory for autophagy. We apologize for the lack of clarity in our original manuscript and have now revised the text to explicitly state this interpretation.

*The revised text states: ""*We also monitored autophagic flux by assessing autophagosome-associated LC3B-II levels following perturbation of ARHGAP15 or ATG16L1 function, in the presence or absence of doxycycline, and in comparison with cells treated with bafilomycin A1, which blocks autophagosome–lysosome fusion (Fig. 3f, Extended Fig. 3a). ATG16L1 depletion resulted in reduced autophagic flux, whereas knockdown of *Arhgap15* led to an increase in flux. In line with our hypothesis that ARHGAP15 acts through ATG16L1, the combined depletion of ATG16L1 and ARHGAP15 inhibited autophagic flux. Together, these findings demonstrate that ARHGAP15 negatively regulates ATG16L1 activity during centrosome-associated autophagy"

To clarify our rational we added the following text:
"ATG16L1 is defined by three principal domains: an N-terminal ATG5/RB1CC1/ATG13-binding domain; a central coiled-coil dimerization domain (CCD); and a C-terminal region containing seven WD40 repeats (C-WD) (Fig. 4a). To gain mechanistic insight into how Arhgap15 and ATG16L1 cooperate in centrosome homeostasis and autophagy, we examined the importance of these domains for their potential interactions. *In vitro* binding experiments of GST-ARHGAP15 and the GTP-locked small GTPase His-RAC1-Q67L confirmed previous findings that ARHGAP15 binds RAC1 isoform (Extended Fig. 4a–b). Strikingly, we found that ATG16L1 also interacts with RAC1-Q67L, placing ATG16L1 as a novel RAC1 interactor. Further mapping revealed that this interaction occurs through the central coiled-coil domain (CCD) of ATG16L1 (Fig. 4b). "

To further investigate this, we tested whether ATG16L1 can bind RAC1 and found that this interaction occurs via ATG16L1's central coiled-coil domain. Updated figure 4 includes this data (Fig4 a-b).

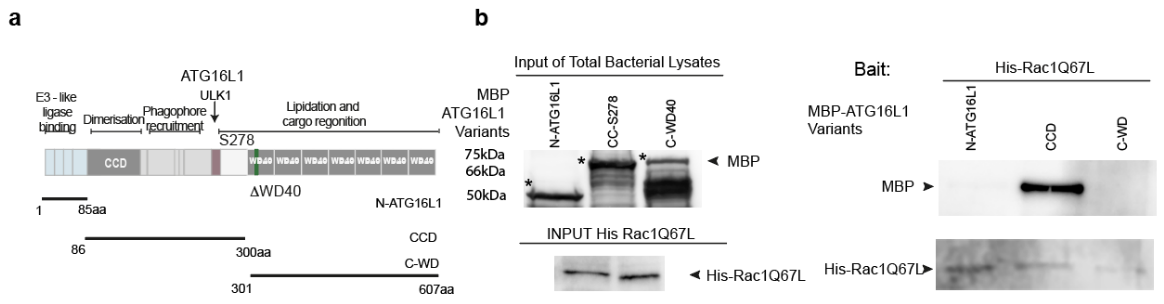

The revised figure legend reads for Fig4 “(b) Binding assay of purified recombinant His–Rac1 Q67L to three distinct ATG16L1 domains fused to MBP (N-terminal, CCD, and WD). Lysates of bacteria expressing each MBP-tagged domain were incubated with His–RAC1 immobilized on cobalt beads. Input and pulldown fractions were analyzed by SDS–PAGE and immunoblotting with anti-MBP and anti-His antibodies, revealing specific binding of His–RAC1 to the ATG16L1 CCD domain. Binding assays were performed in at least three independent experiments”.

To clarify our rationale, we expanded our dataset to define the domains in ARHGAP15 required for its interaction with ATG16L1 (Figure 2A). These experiments demonstrate that both the PH domain and the GAP activity of ARHGAP15 are necessary for its association with ATG16L1, supporting ARHGAP15’s role as a regulator of small GTPases. Based on these findings, we hypothesize that ATG16L1 activity may be modulated through RAC1, a known substrate of ARHGAP15. Please refer to our response to Reviewer 3, where we address the role of RAC1 inhibition in centrosome amplification.

Is the interaction affected by ULK1-mediated phosphorylation (a quantification of at least 3 independent experiments as in Extended Fig. 4 should be quantified)?

To address the potential regulation of the ARHGAP15–ATG16L1 interaction by ULK1-mediated phosphorylation, we provided supporting data in the original Extended Figure 3 showing that ULK1 depletion phenocopies ATG16L1 knockdown - both treatments lead to increased centrosome amplification. Furthermore, we observed significant differences in centrosome number and autophagy levels when comparing cells expressing phospho-mutant and phospho-mimetic forms of ATG16L1, indicating that ULK1-dependent phosphorylation of ATG16L1 plays a functional role in regulating autophagy and, as shown in our study, in centrosome homeostasis.

7. What is the fate of the cells undergoing centrosome amplification and ATG16L1 knock-down/autophagy inhibition? Do they die? Do they undergo mitotic catastrophe? Do they accumulate genomic aberrations? Is cell proliferation affected? Phenotypes other than centrosome amplification should be monitored.

We thank the reviewer for this thoughtful set of questions. In our experiments, knockdown of ATG16L1 in mouse embryonic fibroblasts resulted in reduced cell proliferation, suggesting that autophagy inhibition compromises the proliferative capacity of cells undergoing centrosome amplification. We agree that it would be highly relevant to investigate in detail whether these cells undergo cell death, mitotic catastrophe, or accumulate genomic aberrations. However, a comprehensive analysis of these additional outcomes—both in the presence and absence of centrosome amplification—would require an expanded set of experiments that lies beyond the scope of the present manuscript, which is focused on the mechanistic interplay between ARHGAP15, ATG16L1, and centrosome homeostasis. We greatly appreciate the reviewer’s suggestions and agree that these important questions warrant further exploration in future studies.

8. TIAM1 role in autophagy regulation upon centrosome amplification is brought up very late in the manuscript, and it has been barely validated, but not characterized. Therefore, it does not add to the molecular mechanism underlying the cross-talk between autophagy and centrosome amplification. I would leave it out of this manuscript.

We agree with the reviewer. This is an area that demands future study but is not relevant to the main message of the current paper and so we have removed these data.

9. It is not clear whether the authors hypothesize that autophagy selectively targets centrosomes resulting from centrosome duplication. This should be further clarified and properly tested/investigated. Also, is selective autophagy involved? Are selective autophagy regulators (e.g. selective autophagy receptors/adaptors, “eat me” signals, etc.) involved in the autophagy-mediate escape of cell proliferation blockage upon centrosome amplification?

We appreciate the reviewer's insightful comment. Our data suggest that selective autophagy may indeed be involved in the turnover of amplified centrosomes. Specifically, we observed that p62/SQSTM1, a well-characterized selective autophagy adaptor, co-immunoprecipitates with ARHGAP15 (Figure 2d). While these observations are suggestive, the identification and functional validation of specific selective autophagy receptors and "eat me" signals involved in centrosome clearance are beyond the scope of the current study. This is an area of active investigation in our laboratory, where we are currently characterizing both known and potentially novel adaptors that may mediate this process. We agree that this line of research is highly relevant and could provide further mechanistic insight into the regulation of centrosome homeostasis via autophagy.

10. Autophagy has been reported to degrade centriolar satellites (CS) to maintain centrosome homeostasis (ref. 86 in this manuscript). Are CS involved in the regulation of centrosome amplification/amplified centrosome clearance through autophagy

*We appreciate the reviewer's insightful comments. Indeed, autophagy has been reported to degrade centriolar satellites (CS) to maintain centrosome homeostasis (ref. 86 in this manuscript). Regarding the specific question of whether CS are involved in regulating centrosome amplification or the clearance of amplified centrosomes via autophagy, current evidence remains limited and inconclusive. It is well established that centriolar satellites, such as those marked by PCM1, are dynamic, membraneless granules involved in processes like microtubule nucleation and cilia assembly. The reviewer is also correct in referring to PCM1's interaction with GABARAPL2, which links satellites to the autophagy machinery. We now add reference to another published work about PCM1 by inserting the following statement into the Discussion: "*Joachim and colleagues showed that PCM1 binding to GABARAP, but not another ATG8 family member LC3B, occurred through an LIR motif and that loss of PCM1 destabilizes GABARAP through the proteasome[88]. They found that PCM1 enhances GABARAP/WIPI2/p62-positive autophagosome formation and flux but has no significant effect on LC3B-positive autophagosome formation suggesting one mechanism for centrosome-autophagosome crosstalk.*"*

Minor comments
1. Line 44. "Which is by activated" is probably "Which is activated by"

OK corrected

2. The authors report on an unknown role of PCM1 in autophagy. This is not accurate: Sharon Tooze´s laboratory reported a role for PCM1 in regulating autophagy, through its interaction with GABARAP/L2. Please revise the manuscript discussion accordingly.

OK corrected

Reviewer #2 (Remarks to the Author):

Centrosome amplification has long been described as a feature of human tumors. Despite this well-established association, centrosome amplification can impose negative proliferative effects on cell proliferation. To overcome some of these inhibitory effects, different genome-wide screens have identified the PIDDosome as an inhibitor of cells with extra centrosomes by inhibiting cell cycle via p53 stabilization. However, this complex is not activated in all cell types, and it is likely that other pathways contribute for the proliferation of cells carrying extra centrosomes.

Here, the authors use a CRISPR/Cas9 screening strategy to identify new genes/pathways that allow cells with extra centrosomes to proliferate. They found several genes, whose KO are enriched in cells overexpressing PLK4. Some of the genes are involved in autophagy regulation, such as ARHGAP15. The authors further show that the autophagy protein ATG16L1 is required for centrosome reduction downstream of ARHGAP15 loss and that ARFGAP15 is counteracted by ARHGEF2. The authors propose that activation of autophagy by centriole satellites can prevent centrosome amplification. While overall this work provides a new dimension to how the negative impact of centrosome amplification can be are overcome, there are several issues with how the data is presented and some conclusions lack strong evidence. Clarity and general data presentation could be improved to help readability. There are major issues that should be addressed prior to publication, which are outlined below.

Major concerns:
1. In general, the description of how the screen was conducted is not well explained. It is not clear what was the control used for the screen, were cells without DOX used as control? Or cells treated with DOX but not Cas9? Scheme of Extended figure 1 does not contain this crucial information. This is important because some of the enriched KO could have a general positive impact of cell proliferation. This is a major concern as it is not clear that the treatments/depletions are specific to cells with amplified centrosomes

We greatly appreciate the reviewer's comments and acknowledge that Extended Figure 1 may appear minimalistic. This was primarily due to space constraints within an already overcrowded figure.

*However, the experimental design is described in the main text where we state: "*The Plk4-OE ESC line was first transduced with lentivirus carrying Cas9-EGFP and subsequently with a lentiviral library of single-guide RNAs (sgRNAs), in which each mouse gene was represented by approximately 10 unique sgRNAs[38] (~180,000 sgRNAs total), and cultured in the presence or absence of doxycycline*…"*

We also recognize the reviewer's concern that some of the enriched genes identified in the screen may have a general positive impact on cell proliferation. To address this, we performed a broader phenotypic characterization focusing specifically on centrosome number. We agree that knockdown of Lats2, TP53, and Pidd1 can have general effects on proliferation, and we used these as internal controls in agreement with published data. Importantly, our classification of gene groups was based on both centrosome number and proliferative capacity, and therefore cannot be explained solely by a general effect on cell proliferation.

2. The authors should provide data on induction of centrosome amplification in the screen setting and what happens to centrosome amplification over time? It is known that, upon induction, extra centrosomes are poorly maintained in tissue culture.

We thank the reviewer for their helpful comments regarding the lack of clarity in this section. In response, we have added further labelling to Extended Figure 1 (b) and included a new Supp. Fig. 1a (see also below), which presents immunostaining of mES cells over-expressing Plk4 for 9 days. These images illustrate cells displaying varying degrees of centriole over-amplification (Supp. Fig. 1a).

Additionally, to support our statement regarding ploidy maintenance over time, we now include karyotypic analyses of mES cells after prolonged culture. Specifically, we show a total of 10 karyotypes for mES cells with and without Plk4 overexpression at day 6 of induction (Supp. Fig.1 c-d).

Supplementary Figure 1

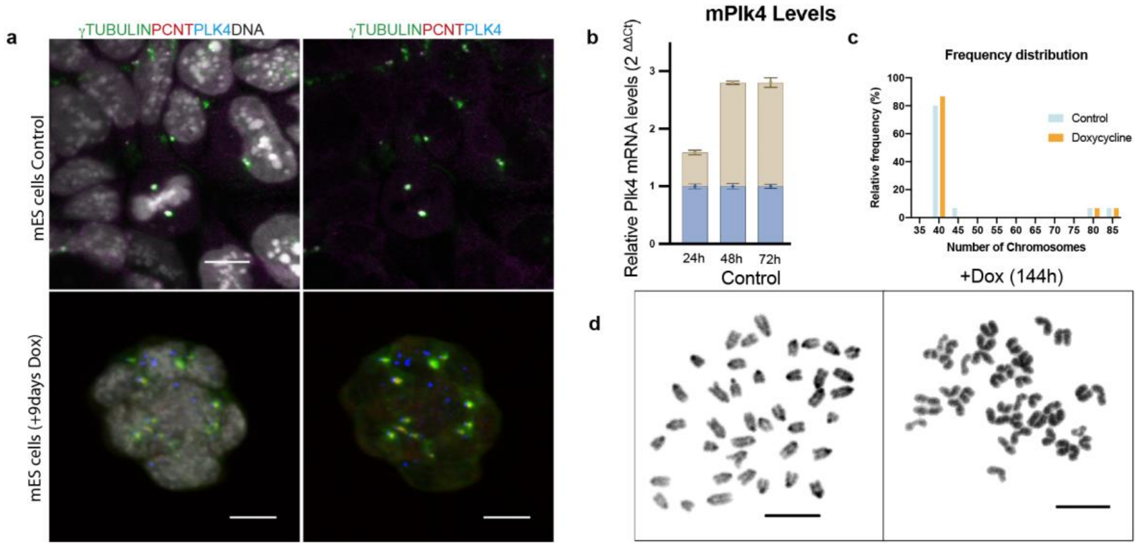

The legend for Supp. Fig.1 states: **Screening conditions in mouse embryonic stem cells (mESCs) and design of the follow-up screen in mouse embryonic fibroblasts (MEFs).** (a) Overexpression of Plk4 in mESCs leads to centrosome amplification. Representative images of control cells and cells treated with doxycycline (+Dox) for 9 days are shown. Immunostaining highlights PLK4 (blue), γ-tubulin (green), and pericentrin (red); DNA is stained with DAPI (white). Magnified centrioles (white boxes) are shown as merged or PLK4-only images. Scale bars are indicated for merged panels. (b) Quantitative RT-PCR analysis of selected transcripts in mESCs during Plk4 overexpression.(c) Karyotyping of control (n = 10) and PLK4 OE (n = 10) mESCs on Day 6. A frequency plot shows that 2n DNA content is unaffected by Plk4 overexpression. Representative karyotypes are shown for control and PLK4 OE cells. (d) Schematic outline of the reconfirmation screen in MEFs. siRNA treatments were applied on Day 0; doxycycline was introduced on Day 1 to induce PLK4 overexpression. Media were refreshed on Day 3, and cells were harvested or fixed on Day 4 (96 h) for immunofluorescence and quantification.

We hope these additions clarify our experimental observations and strengthen the conclusions.

3. Related with the comment above, the authors often state ‘centrosome reduction’ to describe low centrosome numbers. However it is unclear what this means. Are the treatments used reducing centrosomes (for example once these are amplified)? Or do they block centrosome duplication and hence cells will have less centrosomes? This is not the same and should be clarified.

We thank the reviewer for this observation. We agree that our use of the term “centrosome reduction” was vague and requires clarification. At this stage, we cannot definitively distinguish whether the observed reduction in centrosome number arises from active elimination of amplified centrosomes or from the inhibition of further centrosome duplication. This likely depends on the specific mechanism of action of each individual gene or protein identified in the screen. In the case of Arhgap15, our data indicates this encodes a negative regulator of autophagy that can be activated to eliminate centrosomal components thereby keeping centrosome numbers at a level that promotes cell viability. We have avoided use of the word “reduction”

4. The authors should explain why DOX was kept throughout the duration of the screen. Constitutive overexpression of PLK4 can have toxic effects and it needs to be shown whether the loss of viability shown in extended figure a (scheme) is a consequence of centrosome amplification or toxic PLK4 overexpression. If PLK4 is only transiently overexpressed in mESCs, do the cells have the same proliferative defects?

We thank the reviewer for this comment. The mES cells used in the screen were previously used to generate a transgenic mouse line (Coelho et al., 2015). In the course of this previous experimentation, we employed continuous overexpression of Plk4 and this did not affect embryo development throughout pregnancy or subsequent postnatal development. It did have a number of consequences on tissues exhibiting continuous cell proliferation, notably the skin, but was not toxic. Rather the defects predominantly reflected perturbations of the cell division cycle. It therefore appears that as embryos are able to develop to term following the continuous overexpression of Plk4 throughout pregnancy, a particularly exacting requirement, that Plk4 overexpression at the levels promoted in these experiments is not toxic.

5. A major issue is also data presentation. The graphs used to plot centrosome amplification are not intuitive (e.g. fig1c). What are the different ‘little’ bars? Individual cells? Experiments? How many experiments were done and how many cells were quantified in each plot? Also, no stats are shown in any graphs, and thus the significance of the data is not always clear.

We thank the reviewer for raising this point and apologise for any lack of clarity. In the graphs representing centrosome amplification (e.g., Fig. 1c), the "little bars" correspond to the percentage of cells per microscope field (greater than 20 cells per field). Random fields were observed until greater than 200 cells had been examined. This, together with statistical analyses, is indicated in the extended tables. At least three such experiments were carried out for each experimental condition as stated in the text. All of the primary data in given in the extended tables. The graphs presented in the main figures display the mean values, with bars representing the mean ± 95% confidence interval (CI).

6. On line 133 the authors state that proliferation recovery was measured in the MEFs. However, the figure the authors mention (Fig 1b) only refers to mESCs. A validation of proliferation data in MEFs to connect the work to the initial data should be provided as this is mentioned in the abstract as well.

*We thank the reviewer for this comment. We must not have been clear in the original text because the proliferation recovery experiments in Fig. 1b were performed in mouse embryonic fibroblasts (MEFs). This is stated in the figure legend: “*A total of 32 genes selected from the CRISPR/Cas9 screen on ESCs were selected for further study by depletion of their gene products using siRNAs in mouse embryo fibroblasts (MEFs) established from the transgenic mouse carrying tetON-Plk4 (TetON-Plk4 MEFs). Proliferation assays were carried out on TetON-Plk4 MEFs grown in the presence or absence of doxycycline following the knockdown of each of the selected genes. A proliferation recovery index was calculated according to the formula: 100 x [(Gene query siRNA+DOX / Gene query siRNA) - (Control+DOX/Control)]. Data acquired corresponds to a minimum of three independent biological replicates. The error bar indicates mean ± s.e.m.”

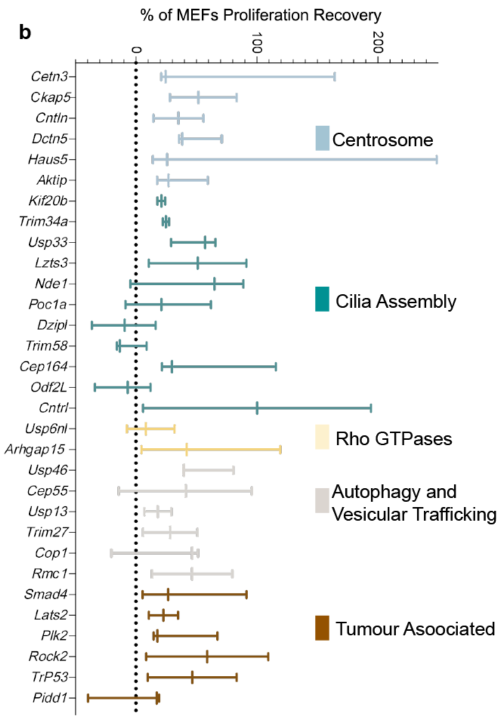

This was indicated in a simplified explanatory scheme summarizing the screen and the validation pipeline (Extended Figure1a) from ESC-based CRISPR screening to MEF proliferation recovery validation.

We have now have added a Supp. Fig.1e showing the experimental workflow in MEFs

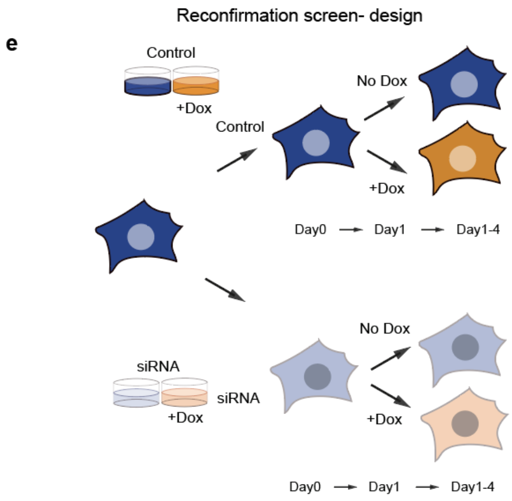

In the Supp. Fig.1 legend reads "(e) Schematic outline of the reconfirmation screen in MEFs. siRNA treatments were applied on Day 0; doxycycline was introduced on Day 1 to induce PLK4 overexpression. Media were refreshed on Day 3, and cells were harvested or fixed on Day 4 (96 h) for immunofluorescence and quantification".

We thank the reviewer again for these valuable suggestions, which have helped us improve the clarity of the manuscript.

7. The phenotypic categories of the different knockdowns seem somehow artificial. For example, category III is not based on phenotype but rather on the known function of these proteins. Lats2 depletion leads to increase in

>2 mothers, while depletion of p53 and Pidd1 doesn't. Also unclear how this information is used in the rest of the manuscript, for example when autophagy is inhibited.

We thank the reviewer for this comment. The gene grouping was based primarily on the phenotypes we analyzed: (1) the ability to sustain proliferation; (2) the impact on centrosome number alone; and (3) the effect on centrosome number in the context of Plk4 overexpression. This classification emerged naturally from the patterns we observed across these assays. Our initial approach to this classification was that genes producing similar phenotypic outcomes might participate in related or converging cellular pathways, potentially acting synergistically within a broader regulatory network. The screen identifies several, not necessarily related pathways. We selected ARHGAP15 for further study as a novel regulator of centrosome number to exemplify the value of the screen and our aim in the subsequent part of the paper was to provide a skeletal view of its function.

Regarding Group III, we agree that this is less phenotype-driven and more functionally defined, as it includes known tumor suppressors including Trp53, Pidd1, and Lats2, which have been previously associated with responses to centrosome amplification. We considered this group as validating the screen as they represent previously characterized genes with expected roles. We appreciate the reviewer's observation that not all genes in this group produced identical effects on centrosome number—specifically, Lats2 depletion led to an increase in cells with >2 mother centrioles, whereas Trp53 and Pidd1 depletion did not. However, knockdown of all facilitated proliferation recovery under Plk4 overexpression, supporting their role in the response to centrosome amplification.

Additionally, during the manuscript revision, we realized that some of the graphs had been inadvertently swapped. This has now been corrected, and statistical analyses have been added, which helps to distinguish the phenotypic differences between the groups.

Finally, we acknowledge that further characterization is needed for several genes, particularly those in Group III and others that modulate centrosome amplification. Work is currently ongoing in our lab to deepen the phenotypic and mechanistic understanding of these candidates, which will be the subject of future studies.

8. If the authors want to propose that RAC1 activity is crucial in reducing extra centrosomes, then a more direct experiment should be used to test this idea. For example, inhibiting RAC1 in cells depleted of ARHGAP15 KO to analyze centrosome number could be used. As it stands, link to RAC1 is weak despite being described in the abstract.

We appreciate the reviewer's comment and fully agree that a more direct experimental approach would strengthen the link between RAC1 activity and centrosome number regulation. In response, therefore, we performed the suggested experiment: we inhibited RAC1 in ARHGAP15 knockout (KO) cells and quantified centrosome numbers. The findings demonstrate that RAC1 activity indeed contributes to the regulation of centrosome number in this context and so accords with our previous observations. We have incorporated these new results in the revised manuscript as Extended Figure 2 (below).

The legend reads: " **Extended Figure 2. ARHGAP15 is a negative regulator of autophagy, and RAC1 regulates centrosome amplification.** (a) *Tet^{ON}-Plk4* MEFs stably expressing hARHGAP15 or the hARHGAP15 R317M mutant (R317M) were stained to visualize centrosomes following siRNA-mediated knockdown of endogenous mouse *Arhgap15* and *Atg16l1*, in the absence or presence of doxycycline (+Dox) to induce PLK4 expression. Representative immunofluorescence images are shown. (b) Quantification of the percentage of cells with 0, 1–2, or >2 centrioles pairs per cell. Data are displayed as yellow or orange bars; error bars represent mean ± 95% confidence interval (CI). p values are indicated as ****$p < 0.0001$, ***$p < 0.001$, **$p < 0.01$, *$p < 0.05$ (black, control vs. control + Dox; green, control vs. equivalent Plk4OE conditions of PLK4 overexpression). Sample sizes and additional p values are provided in Extended Tables 4. **(c)** Representative mitotic figures of control *Tet^{ON}-Plk4* MEFs and *Tet^{ON}-Plk4* MEFs depleted of ARHGAP15 by siRNA, cultured with or without doxycycline. RAC1 activity was inhibited with EHT 1864 in control MEFs or following *Arhgap15* siRNA, in the presence or absence of doxycycline. Immunostaining reveals PLK4 (red), the distal appendage protein Cep164 (green), and α-tubulin (white, spindle). CEP164 marks mother centrioles throughout the cell cycle, enabling scoring of centrosome amplification corresponding to >2 mother centrosomes per cell. Scale bar, 10 µm. Insets highlight individual centrosomes at higher magnification. **(d)** Quantification of the percentage of cells with 0, 1–2, or >2 mother centrioles per cell, displayed as yellow or orange bars. Error bars represent mean ± 95% CI. Significance and statistical annotations are as in (b). Sample sizes and additional p values are provided in Extended Data Tables 4."

Extended Figure 2

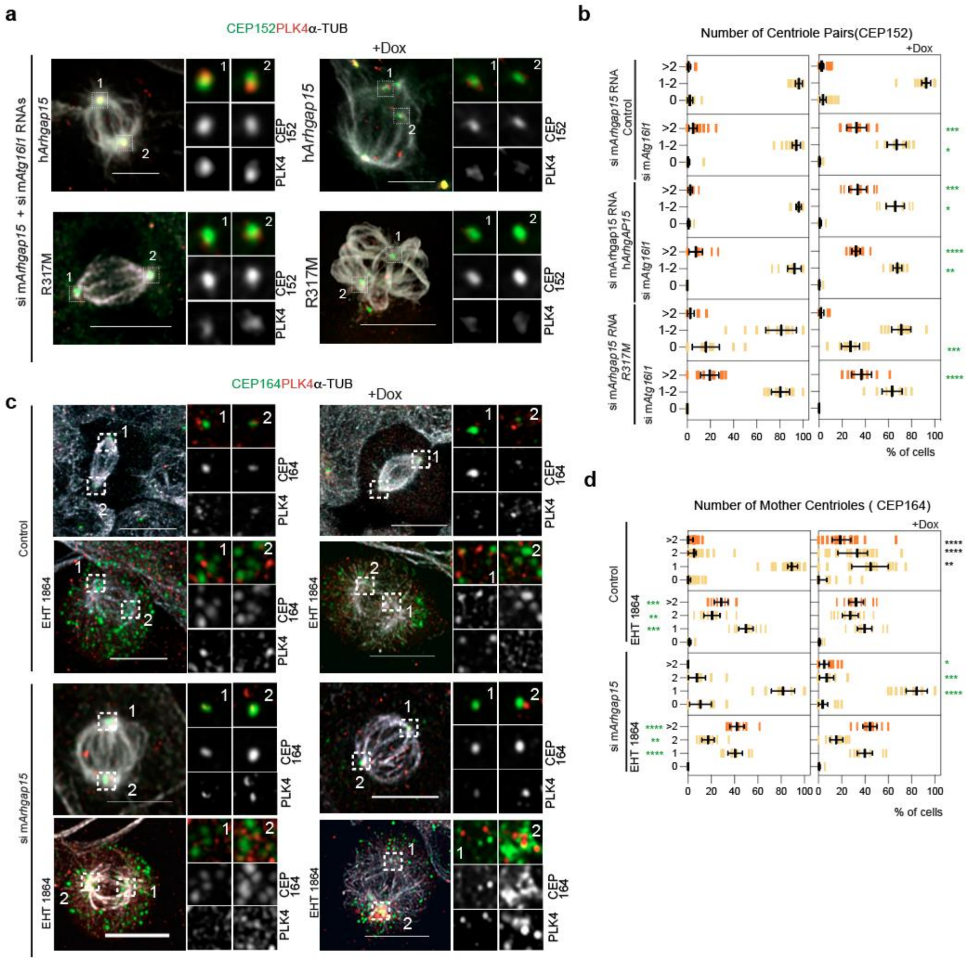

We have added the following text: “Overall, these findings suggest that supernumerary centrosomes can be eliminated through autophagy and that Arhgap15 regulates this process by controlling the active Rac1–GTP form. To directly test the involvement of Rac1, we inhibited its activity using EHT 1864, a selective Rac1 inhibitor that prevents guanine nucleotide binding and thereby blocks Rac1 activation (Shutes et al., 2007). Treatment with EHT 1864 led to a significant increase in centrosome amplification (Extended Fig. 2c–d), independent of Arhgap15 levels. These results indicate that Rac1—the only specific interactor of Arhgap15 identified to date—regulates centrosome amplification through autophagy, establishing Rac1 as a key mediator linking autophagy to the elimination of supernumerary centrosomes.”

Moreover, these data complement the additional pull-downs and binding assays we have performed and now included in the revised manuscript (Extended Figure 4 and Figure 4), further supporting the connection between ARHGAP15, RAC1 activity, autophagy and centrosome homeostasis.

We thank the reviewer for this constructive suggestion, which has helped us strengthen our study.

9. All pull-down/IP experiments need controls (e.g control for FLAG non-specific binding in Figure 2d) and WB for whole cell lysates.

We have added this information into Figure 2, panel d, which is reproduced about together with the amended figure legend.

New Figure 2D

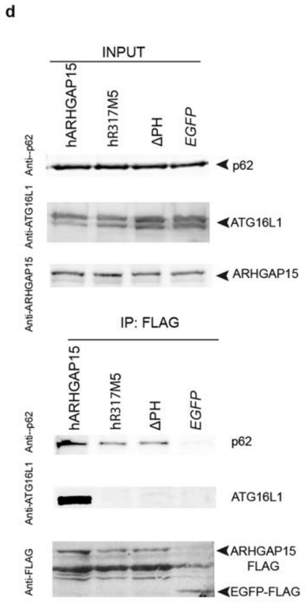

*The updated legend now includes the description of the control: "***(d)** Extracts of Plk4OE MEFs transfected with FLAG-tagged wild-type ARHGAP15, the R317M GAP-deficient mutant, the ΔPH mutant lacking the Pleckstrin Homology (PH) domain, or EGFP-FLAG control, as indicated. Total cell extracts (input) and anti-FLAG immunoprecipitates were analyzed by Western blotting. ARHGAP15-FLAG expression served as loading control and is observed with Anti-ARHGAP15 or anti-Flag antibodies in input or pull downs respectively. ATG16L1 and p62/SQSTM1 were detected in inputs and pulldowns using specific antibodies. Pulldown experiments were performed in three independent replicates."

10. The authors propose that inhibition of the autophagy pathways leads to centrosome amplification even in control cells (figure 3h). Can the authors demonstrate that these inhibitors do not cause cytokinesis failure, which would lead to increased centrosome numbers? Images provided on this figure are not great, microtubules not easy to see for all images and centrosome channels should be separated for better visualization.

We appreciate the reviewer's comment and would like to comment that centrosome amplification per se can lead to cytokinesis failure. For this reason, we have paid careful attention to the DNA content of the cells we analyze to avoid this confounding factor. We see no variation in the size of cell nuclei strongly suggesting that there are no cytokinesis defects during the time of the experiment. This is evident in the images of the treated cells presented in Figure 3g (reproduced below).

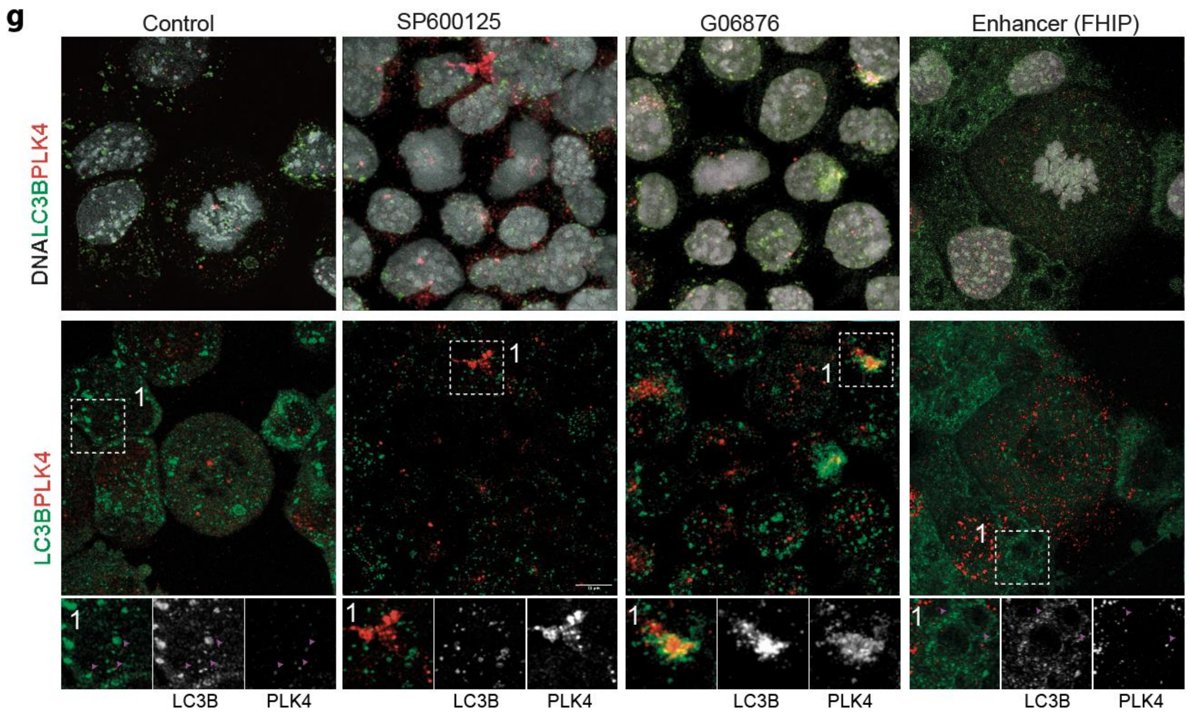

We also acknowledge the reviewer's concern regarding the image quality in Figure 3h. We have therefore provided an alternative image (new Figure 3 h-i)). Please be aware, however, that increased centrosome number also affects microtubule and therefore spindle assembly.

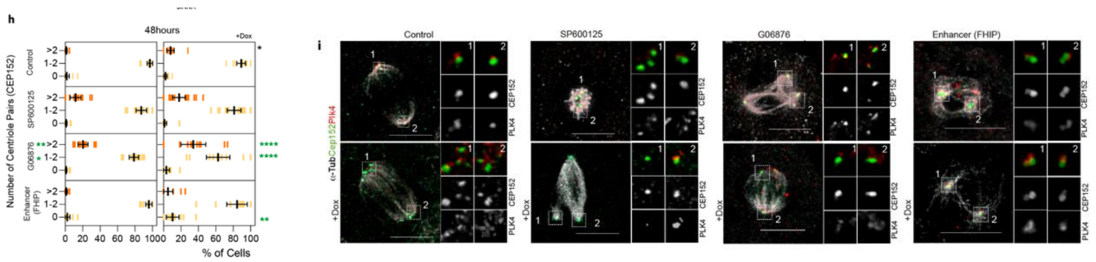

(Note Centrosomes with separated channels)

11. The data describing phagophore-like vesicles in figure 4f seems irrelevant or its relevance is not well explained.

We thank the reviewer for this comment and the opportunity to clarify this point. We consider this finding to be both relevant and exciting, as it reveals that vesicle-like structures positive for Atg16L1 are capable of encapsulating Plk4 and Cep152 — two essential and upstream components of the centriole duplication pathway. The sequestration and colocalization of Plk4 and Cep152 with Atg16L1-positive vesicles suggest a potential mechanism by which autophagy-related structures may directly regulate centrosome biogenesis by targeting key duplication factors.

12. **The authors should test if depletion of ARHGEF2 compromises cytokinesis, which would also lead to extra centrosomes.**
See response to comment 10.

13. Images provided on fig 5b are not very clear. Centrosome channels need to be separated to enable proper visualization.
We have separated the channels as requested. Please see below.

New Figure 3i

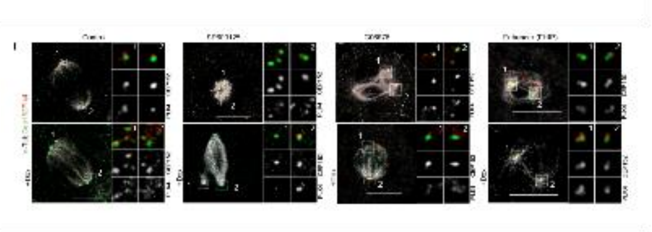

New Figure 5b

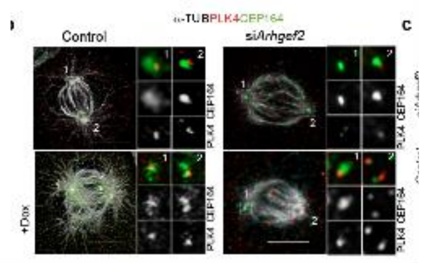

New Extended Figure 4c

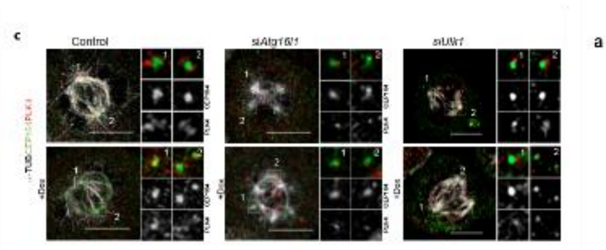

New Extended Figure 6a

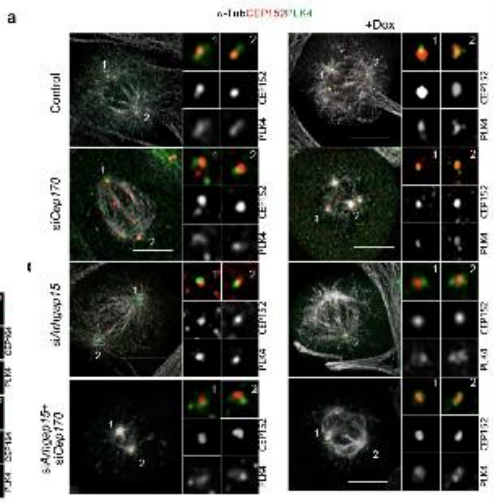

New Figure 4c

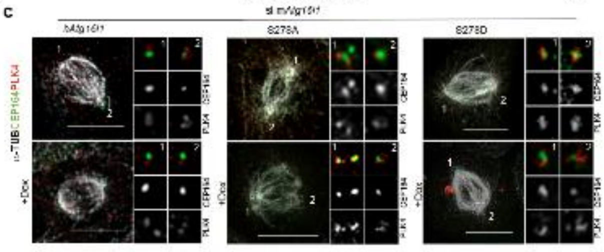

14. Authors state that LC3B vesicles co-localise with CEP170 and accumulate on autophagosomes after PLK4 overexpression. However, there is no quantification for these data and since not all cells will have extra centrosomes (even after DOX), the authors should analyses cells with and without amplified centrosomes in the +DOX condition and determine if the association of autophagosomes with hCEP170 depends on centrosome amplification. This will help understading the specificity of the phenotypes to cells with extra centrosomes.

Ext Fig. 5b presents the quantification of centrioles with CEP170 in two conditions – control and following centrosome amplification. We have added Ext Fig. 5d the quantification of LCB3 puncta colocalising with CEP170.

15. The graphs in figure 6i are not intuitive and difficult to follow.
These represent an alternative way of presenting the distributions depicted in the histogram analyses of the other figures. We adopt this form of graphical presentation here as it more readily allows comparisons of the distribution around the mean values which are being compared in these experiments. Alternatively, the same data is presented in "conventional graphical representation" in Ext Fig. 6.

Reviewer #3 (Remarks to the Author):

Through genome-wide screens, the Glover lab identified the RAC1-specific GTPase-activating protein ARHGAP15 as a novel factor permitting centrosome amplification induced by PLK4 overexpression. To understand how loss of ARHGAP15 suppresses centrosome amplification, the authors found that the autophagy protein ATG16L1 is an interacting partner of ARHGAP15, that ARHGAP15 is a negative regulator of ATG16L1-mediated autophagy, and that ATG16L1-mediated autophagic fluxes are required for centrosome reduction induced by loss of ARHGAP15. Moreover, the authors identified ARHGEF2 as the guanine-nucleotide exchange-factor (GEF) counteracting ARHGAP15, which activates RAC1 and autophagy to reduce centrosome numbers. Furthermore, to understand how supernumerary centrosomes are linked to autophagy activation, the authors found that the centrosomal protein CEP170 is in complexes with ARHGEF2, that CEP170 disassociates from amplified centrosomes, and that CEP170 depletion induces autophagy reduction while causing/enhancing centrosome amplification specifically in the absence of PLK4 overexpression, consistent with an idea that CEP170 triggers ARHGEF2-mediated autophagy to regulate centrosome numbers. Together, the authors suggest that autophagy is a key regulatory module of centriole duplication.

The overall result is important and should be published as it uncovers several previously unrecognized points including:
a). Autophagy ensures proper centriole duplication.
b). "CEP170 -> ARHGEF2/ARHGAP15 -> RAC1 -> ATG16L1" connects centriole duplication to autophagy.
c). Uncovering a direct role of RAC1 (together with its associated GEF/GAP) in regulating autophagy adds additional angles through which RAC1 alterations can impact a variety of diseases.

We thank the reviewer for these encouraging remarks.

Please address, clarify, or discuss the following points as they appear in the result, which I believe can be helpful for readers:

1. The centrosome amplification phenotype, which was described as "CEP152 double dots", is not very clear to me. The authors marked two areas of mitotic cells with centrosome amplification to show "CEP152 double dots", but more often than not, I could not tell if they are double dots or clusters. If centrosomes were amplified in these cells, why select only two areas (two poles?)? If extra centrosomes are randomly clustered to the two spindle poles, why specifically two centrosomes per pole (double dots)?

We thank the reviewer and have replaced the term "CEP152 double dots" with "centriole pairs" or "clusters" as appropriate. We also revise the description of the scoring method in Supp. Fig. 1f.

To address this, we also now provide magnified images with separated channels for the centriolar markers, allowing better visualization of the centrioles within these clusters as part of Fig. 3i, Fig. 4a, Fig.5b, Ext. Fig. 3i and Ext.Fig. 6a (please see response to referee 2).

Please note with reference to the specific images shown above in response to referee 2 that while multipolar spindles are frequently observed in mitotic cells with centrosome amplification, for illustrative purposes we selected two spindle poles where clustering was evident to illustrate this phenotype. This was done to clearly demonstrate centrosome amplification in an interpretable way, rather than to imply that amplification is limited to two centrosomes per pole.

2. Why does expressing ARHGAP15-R317M in the absence of wild-type ARHGAP15 lead to significantly more cells (~16%) that lose their centrosomes, and even more so (25-30%) when PLK4 is overexpressed? Similarly, why does knocking down endogenous ARHGAP15 alone did not cause cells to lose centrosomes when PLK4 is not overexpressed? Does overexpressing ATG16L1 cause centrosome loss?

We thank the reviewer for this important question and agree that further clarification will be necessary in future work.

3. If autophagy ensures proper centriole duplication, why does ATG16L1 depletion not cause centrosome amplification as a default (without PLK4 overexpression), given that the autophagy pathway is off? Why does it depend on PLK4 overexpression? Perhaps ATG16L1 depletion *is incomplete?*

We thank the reviewer for this insightful question. We agree that depletion of ATG16L1 alone does not lead to a dramatic increase in centrosome number under basal conditions. However, when PLK4 is overexpressed -— a condition that forces centriole overduplication -the effect of ATG16L1 depletion becomes apparent. These data suggest that autophagy, mediated by ATG16L1, acts as part of a safeguard mechanism that is particularly engaged when cells are challenged with centrosome amplification. Again, further experimentation would be required, which we believe to be beyond the scope of the current paper.

4. I think in Fig. 4f, the CEP152 and EGFP panels were swapped.

This is correct – we apologize!!. The panels had been swapped and have now been correctly accordingly.

5. Does ARHGEF2 co-localize with CEP170 at the appendage of mother centrioles? If so, does the loss of CEP170 upon centrosome amplification trigger the loss of ARHGEF2 from mother centrioles as well?

We thank the reviewer for this pertinent question, which prompted us to further investigate the potential colocalization of ARHGEF2 and CEP170 at the centrosome. This prompted us to examine this question and, while the pilot experiments suggest that this might be the case, we feel that further studies are required and that this will become beyond the scope of the current paper.

6. Why does the enhancement of centrosome amplification by CEP170 depletion seen only when PLK4 is not overexpressed? Could it mean that there are other centriolar components mediating the condition under PLK4 overexpression?

We appreciate the reviewer's question and agree that CEP170 is unlikely to be the sole protein mediating this process. In the revised discussion we indicate that centriolar satellites may also play an additional role. The relationship between the roles of CEP170 and Centriolar Satellites in this respect will require future study.

Reviewer #4 (Remarks to the Author):

I co-reviewed this manuscript with one of the reviewers who provided the listed reports. This is part of the Nature Communications initiative to facilitate training in peer review and to provide appropriate recognition for Early Career Researchers who co-review manuscripts.

We appreciate the reviewer's work and comments.

REVIEWER COMMENTS

Reviewer #2 (Remarks to the Author):

In the revised version of their manuscript, the authors addressed several of the issues raised during the revision, which include the addition of new data to support their findings. There are still some concerns regarding the clarity of data presentation/description, and it will be up to the authors to decide how they want their work to be perceived and understood. Below, are the original points raised that have not been fully addressed.

1. While we understand the gene groupings decided by the authors take into account centrosome number and proliferation, there is no data to show that some of these genes do not impact proliferation in control -DOX cells. Hence, claims of the specificity of proliferation recovery for cells with amplified centrosomes cannot be made. This should be either addressed with data or by ensuring that the text states that the authors cannot exclude that impacts on proliferation may be independent of centrosome number (even if centrosome numbers are reduced).

The proliferation "recovery" score for each siRNA was ***explicitly normalised to its effect in –DOX cells****. In other words, for each gene, proliferation in +DOX cells was compared to the corresponding –DOX condition for the same siRNA, so that any general impact of gene depletion on proliferation in control cells is intrinsically taken into account in the analysis (Fig. 1b). This is described in the formula for calculation of the Proliferation recovery. Thus, with respect, we did not classify genes solely based on their behaviour in +DOX cells.*

That said, we fully agree with the reviewer that, even having normalised the data in this way, we ***cannot completely exclude*** *that reduced expression of some genes might affect proliferation independently of centrosome amplification. This is clearly the case for canonical cell-cycle regulators such as p53 or LATS2, whose depletion is known to promote proliferation in a broader context and not only in cells with extra centrosomes. Importantly, we have not claimed in the manuscript that these genes act* exclusively *in cells with amplified centrosomes. Rather, our intention was to state that these genes* ***enable or promote proliferation in the presence of extra centrosomes****, which is not equivalent to saying that their effects are restricted* only *to cells with centrosome amplification.*

We have therefore modified the text of the discussion (line 433-435) to state: *"We* ***cannot exclude*** *that reduced expression of some genes might affect proliferation independently of centrosome amplification. This is clearly the case for canonical cell-cycle regulators such as p53 or LATS2"*

2. We appreciate the attempt to demonstrate centrosome amplification. However, as it is known that the amplification is lost in vitro over time, the authors should quantify the levels of centrosome amplification after 4ug/ml DOX treatment, and again after 9 days as asked in the initial revision. The images do not provide quantitative data, which I am sure the authors would agree.

We thank the reviewer for this comment and for the opportunity to clarify the behaviour of the mouse embryonic stem cells (mESCs) in this assay. In this system, continuous exposure to 4 µg/ml DOX and subsequent Plk4 over-expression has an effect in viability and proliferation. By day 9, mES cultures show a typical quiescent stage characterized by cells being largely detached and floating in the medium. Thus, it is not possible to perform a meaningful long-term time course of centrosome amplification.

As shown in Fig. 1c, 4 µg/ml DOX induces robust centrosome amplification at the early time point used throughout the study. To address the reviewer's request as far as feasible, we have quantified centrosome number after 9 days of Dox treatment. The culture was initiated with 1 × 10^6 mESCs per dish and after 9 days of DOX treatment only ~500 cells remained adherent. Among these rare surviving cells, approximately 90% exhibited amplified centrosomes. We have added this comment in the figure legend of supplementary Figure 1, that these cells represent the small residual population present at day 9, when the vast majority of the culture is no longer viable.

4. While the constitutive Plk4 expression may not impact embryonic viability, this does not account for proliferative defects and cytotoxic effects within the cells. This is what the original question was to address. At the present, the authors cannot exclude that within a heterogenous population of cells, PLK4 constitute OE has no cytotoxic effect. The authors could test this by comparing PLK4 transient OE. Furthermore, as no data is provided to the extent of centrosome amplification relative to extended figure 1a (viability data) relative to the concentration of DOX, it is impossible to tell if the loss of viability is associated with levels of centrosome amplification or PLK4 OE.

*We could not find a convincing example where **PLK4 overexpression is toxic** **without** **centrosome amplification**. Where this has been tested, toxicity tracks with extra centrosomes, not with PLK4 itself. We refer to the following papers:*

- ***Holland et al., Genes & Dev 2012** – Preventing PLK4 autoregulated degradation stabilizes PLK4, causes **centrosome amplification**, p53 stabilization and loss of proliferation. Here, the interpretation is that amplified centrosomes activate p53 and that loss of proliferation is not the result of centrosome-independent PLK4 toxicity.*

- ***Levine et al., Dev Cell 2017 ("Centrosome amplification is sufficient to promote tumorigenesis")** – Dox-inducible Plk4 in mouse skin. The authors explicitly use **SAS-6 depletion** to block centriole duplication: when extra centrioles are prevented, the Plk4-induced phenotypes (mitotic errors, inflammation, tumorigenesis) are lost, arguing strongly that the cell proliferation arrest comes from extra centrosomes, not PLK4 overexpression per se.*
- ***Arnandis et al., Nat Commun 2018 ("Oxidative stress in cells with extra centrosomes…")** – This uses a similar inducible Plk4 system. The authors show ROS production and non-cell-autonomous tumor promotion depend on **extra centrosomes**, and say explicitly that these effects are **"not due to unspecific effects of PLK4 overexpression or DOX treatment"**, again using SAS-6 depletion to make the point.*
- ***Shin et al., 2021 ("Generation and fates of supernumerary centrioles in the developing epidermis")** – Plk4 overexpression in mouse epidermis gives centrosome amplification, multipolar divisions, p53 elevation and apoptosis. This again is framed as a consequence of extra centrosomes.*
- ***Braun et al., Sci Adv 2024 ("Extra centrosomes delay DNA damage–driven tumorigenesis")** – A DNA-damage model with Plk4-driven centrosome amplification. Apoptosis in response to damage is associated with **Plk4-overexpression–induced centrosome amplification**, not to Plk4 alone.*

*That said, there are **non-centrosomal functions** of PLK4 that have been described including actin/ARP2/3-dependent migration; EMT/anoikis resistance; and effects on the tumour microenvironment. These mostly represent **pro-survival or pro-invasive** effects, not toxicity, and are explicitly argued to be* independent *of PLK4's centriole-duplication role (e.g. Luo et al. Nat Commun 2019; Fonseca et al. Cell Death Dis 2023; summarized in Byrne 2020 and Garvey 2021).*

*We will therefore add a sentence to the Introduction (line 73-76) to clarify this as follows: "Although one cannot exclude toxic effects due to Plk4 over-expression, the consequential inhibition of cell proliferation or apoptosis has been directly linked to centrosome amplification **(Arnandis et al., Nat Commun 2018)** whereas non-centrosomal PLK4 functions reported to date are predominantly pro-survival or pro-invasive (Luo et al. Nat Commun 2019)."*

5. In general, data should be presented per experiment, and it should be clear which fields correspond with each experiment. The current representation of the data does not allow the reader to assess variability between experiments, which is an issue. We appreciate that the authors added statistics to some of the graphs, although not to all. Where stats have been added, it is difficult to assess what is being compared and what is significant. Furthermore, whilst we appreciate that stats analysis can clutter figures, it is important to have the information available in the figure rather than in statistics tables. It is puzzling that this is not done.

We agree that should be possible for the reader to assess variability and to understand clearly which comparisons are statistically significant.

It is for this reason we have shown the following in both the original and revised version of the manuscript:

1. ***We have displayed all individual data points for each condition** in all quantification plots, rather than showing only summary values/experiment. Each point corresponds to a single cell/field (as indicated in the legends) representing data pooled from at least 3 independent experiments. The number of independent experiments (≥3) and total number of cells/fields (n) is included in each table. This representation allows the reader direct access to the full distribution of the data and the underlying variability, without down-sampling the information to a small number of experiments with 3 means and or standard errors.*
2. ***We have added statistical information directly to the figures for all analyses with calculated p values.** For each graph, we indicate:*
   - *the statistical test used (e.g. one-way ANOVA with appropriate post hoc test, or the corresponding non-parametric test),*
   - *the comparisons that were performed, and*
   - *the significance levels, using conventional asterisks (defined in each table). Only the statistically significant values are shown and non-statistically significant (ns) values are not included). The values being compared are indicated in the figure legend(s). Other comparisons are possible in multiple combinations by accessing the excel files. There is a limit of space for figures and text and, in our opinion, nothing is better that the excel files to enable the curious reader access to all the data.*
3. ***We have provided detailed descriptions of the statistical analysis in the figure legends and excel source data files.** The legends specify the test, the exact comparisons, and the definition of N. Exact numerical values (means, 95% of Confidence Interval, and p values) are included in the accompanying source data files, so that curious readers can also examine the data at the level of individual experiments if they so wish.*

We believe that by presenting all data points together with clearly annotated statistical tests, and a full reporting of the number of experiments and all values, we allow readers to robustly assess variability and significance. The additional tables are required to present all the data and we believe this is clearly stated.

10 and 12. The authors state that they paid careful attention to DNA content. Please could the authors provide evidence of this, and that the cells are not undergoing cytokinesis failure? At least ARHGEF2 has known roles in cytokinesis: https://pubmed.ncbi.nlm.nih.gov/17488622/

To address this point, we have now included the DNA (DAPI) channel for the ARHGEF2-depleted cells in the revised Figure 5 (now presented in Supplementary Figure 2a). The added panels show that the cells selected for analysis display an apparently normal DNA content.

More generally, throughout the study we systematically excluded from our quantifications any cells showing obvious signs of cytokinesis failure, such as binucleation, multinucleation, or markedly increased DNA content based on DAPI staining. Thus, the centrosome phenotypes reported for siARHGEF2 are quantified only in cells with a single nucleus and no detectable increase in DNA content. This was already stated in the answer to the previous revision'

We add the statement: "Cells displaying signs of cytokinesis failure associated with siArhgef2 depletion (binucleated or multinucleated nuclei, or markedly increased DAPI signal) were excluded from all quantifications." (please see line 354-356).

14. According to the authors model in figure 6l, increased autophagy of centrosome components in the absence of ARHGAP15, leads to loss of amplified centrosomes in cells treated with DOX. This is also supported by their data in figure 1c where loss of ARHGAP15 in control (-DOX) cells has no impact on normal centrosome number. Thus, according to the authors this is specific for cells with extra centrosomes, otherwise the expectation would

be that ARHGAP15 loss drives centrosome loss in normal cells as well. Thus, what was asked to the authors, maybe not as clearly as it should, was to test that co-localization of LC3B and CEP170 puncta in ARHGAP15 depleted +DOX cells with and without amplified centrosomes.

Our model in Fig. 6l implies that loss of ARHGAP15 relieves the negative regulation of centrosome-directed autophagy. This effect is expected to occur in all cells, including those with PLK4-induced centrosome amplification. As the reviewer notes, ARHGAP15 depletion has no detectable effect on centrosome number in −DOX cells (Fig. 1c), which is consistent with a regulatory mechanism in which, under basal conditions, normal centrosomes do not efficiently signal for autophagic degradation. We propose that centrosomes trigger autophagy when they are amplified but we believe there exists also a basal level of autophagic flux as shown by the data showed and as discussed.

The existence of a centrosomal signal mediated by CEP170 is an integral part of this model; the observed co-localisation of CEP170 with LC3B is compatible with a mechanism in which CEP170, is mobilised from centrosomes to microtubules, particularly under conditions of centrosome amplification, to contribute to trigger autophagy and to target centrosomal material to autophagosomes. We find CEP170 is not the only centrosomal protein present in LC3B-positive vesicles and targeting CEP170 alone would not be expected to counteract centriole duplication per se. This would require elimination of key components directly involved in the ability of centrioles to duplicate. In support of this we show enhanced centrosome-directed autophagy upon ARHGAP15 loss, we also observe localisation of the distal appendage marker CEP164 and of PLK4 within LC3B-positive vesicles in −DOX cells and, more prominently, in +DOX cells (Fig. 3a–e), consistent with an overall increase in autophagic flux of centrosomal material. We, therefore, do not see why the specific request would help to clarify any additional point. We stress that our model is "a model" and we are hopeful that it will be tested in subsequent studies by our group and others.

Reviewer #3 (Remarks to the Author):

This is an important study revealing the number control of centrosome assembly by autophagy through a molecular pathway involving CEP170-ARHGEF2/ARHGAP15-RAC1-ATG16L1. While I fully support its publication, the complete centrosome loss phenotype induced by the R317M mutant of ARHGAP15 even when PLK4 is elevated (see Figure 2b and Extended Figure 2b) is unusual/unexpected with regard to the current understanding of centrosome assembly or other data provided in this study. I think it should be pointed out and discussed.

We thank the reviewer for this insightful comment. We agree that the near-complete centrosome loss induced by the R317M mutant, even under conditions of elevated PLK4, is striking and at first sight unexpected given the central role of PLK4 in driving centriole duplication. Consistent with this, ARHGAP15 depletion shows a milder effect than expression of the GAP-dead R317M mutant. In our view, these findings point to the existence of modifiers of ARHGAP15 activity with a direct impact on centrosome homeostasis and we now explicitly highlight and discuss this point in the revised manuscript. Line 248-251.

"This unusually strong centrosome loss phenotype of R317M, even in the presence of elevated PLK4, suggests that hyperactivation of the RAC1–ATG16L1–dependent autophagy can override PLK4-driven centriole biogenesis, pointing to additional layers of regulation of ARHGAP15 and centrosome homeostasis that remain to be elucidated."

Reviewer #4 (Remarks to the Author):

I co-reviewed this manuscript with one of the reviewers who provided the listed reports. This is part of the Nature Communications initiative to facilitate training in peer review and to provide appropriate recognition for Early Career Researchers who co-review manuscripts.
