# Supplementary material for: Sensing centrosome amplification: the interface between centriole duplication and autophagy
Source: Nat Commun. 2026 Jun 21;17:7815. doi: 10.1038/s41467-026-74702-9 (PMC13439419; doi:10.1038/s41467-026-74702-9)
Supplement: Supplementary file 3 [file 41467_2026_74702_MOESM1_ESM.pdf]

Supplementary Materials

Sensing Centrosome Amplification: the Interface between Centriole Duplication and Autophagy

Paula A. Coelho, Agnieszka Fatalska, Marco Geymonat, Ramona Lattao, David M Glover

This file contains Supplementary Fig. 1-7 and Source Data 1-2 related to Supplementary Fig. 3a and Supplementary Fig. 4, respectively.

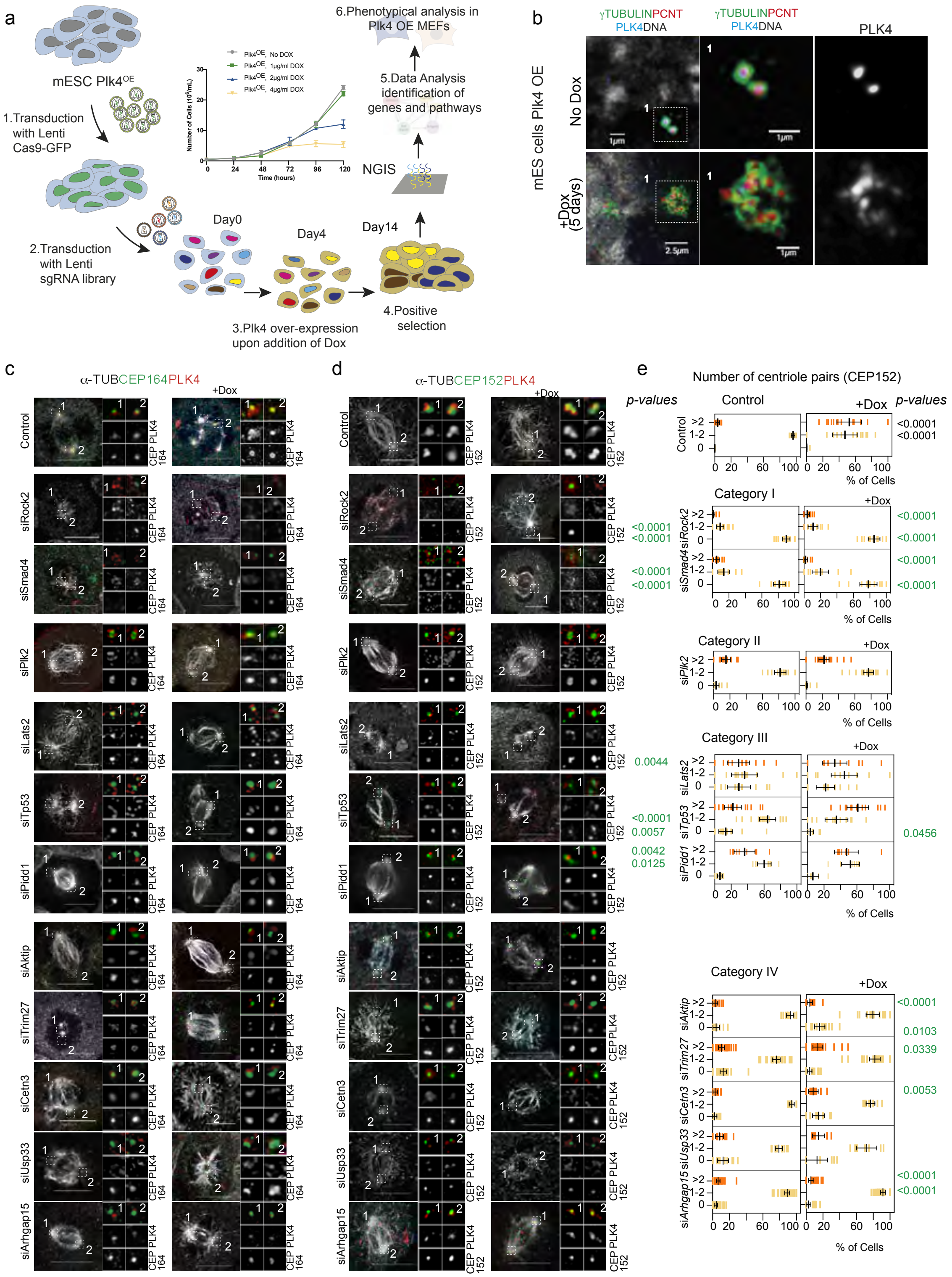

Supplementary Fig. 1 Characterization of CRISPR–Cas9 hits affecting centrosome surveillance in MEFs.

(**a)** Schematic of the screening strategy. Mouse embryonic stem cells (mESCs) carrying a doxycycline-inducible *Plk4* transgene were infected with lentivirus encoding Cas9–EGFP. EGFP-positive cells were selected and clonal isogenic lines established. Cells were then transduced with a genome-wide sgRNA library and cultured for 4 days without doxycycline. *Plk4* overexpression was induced with doxycycline (4 μg ml^{-1}), with media changes every 2 days and passaging on days 8 and 14. After 13 days, genomic DNA was extracted and sgRNAs amplified for Illumina sequencing. (**b)** *Plk4* overexpression (+Dox) induces centrosome amplification in mESCs. Representative images before and after doxycycline treatment show PLK4 (blue), γ-tubulin (green), pericentrin (red), and DNA (DAPI, white). Insets highlight centrioles. Scale bars, 10 μm and 2 μm as indicated. (**c)** Representative mitotic Tet^{ON}–*Plk4* MEFs following siRNA-mediated knockdown of candidate genes, cultured −Dox or +Dox for 5 days. Immunostaining shows PLK4 (red), CEP164 (green), and α-tubulin (white). CEP164 marks mother centrioles. Scale bar, 10 μm. Quantification is shown in Fig. 1. (**d)** Representative mitotic cells following siRNA knockdown, cultured −Dox or +Dox, stained for PLK4 (red), CEP152 (green), and α-tubulin (white). CEP152 labels all centrioles; amplification is defined as >2 centriole pairs per cell. Scale bar, 10 μm. (e) Quantification of centriole number per cell (0, 1, 2, >2). Individual values are shown in yellow, with amplified centrosomes (>2) in orange; bars represent means ± 95% CI. Data are from independent biological replicates: n = 10 for Control, Control +Dox, siSmad4, siSmad4 +Dox, siTrp53, siTrp53 +Dox, siCetn3, siCetn3 +Dox, siLats2 and siArhgap15 +Dox; n = 8 for siRock2, siRock2 +Dox, siPlk2, siLats2 +Dox, siPidd1 +Dox and siAktip +Dox; n = 9 for siPlk2 +Dox and siUsp33 +Dox; n = 11 for siTrim27, siTrim27 + Dox, siUsp33 and siArhgap15; n = 7 for siPidd1 and siAktip. Statistical analysis was performed using Kruskal–Wallis test followed by Dunn's multiple comparisons test. P values are indicated in black for control −Dox versus control +Dox, and in green for control versus corresponding samples under equivalent Plk4OE conditions. Sample sizes and full statistical details are provided in Source Data Supp. Fig. 1.

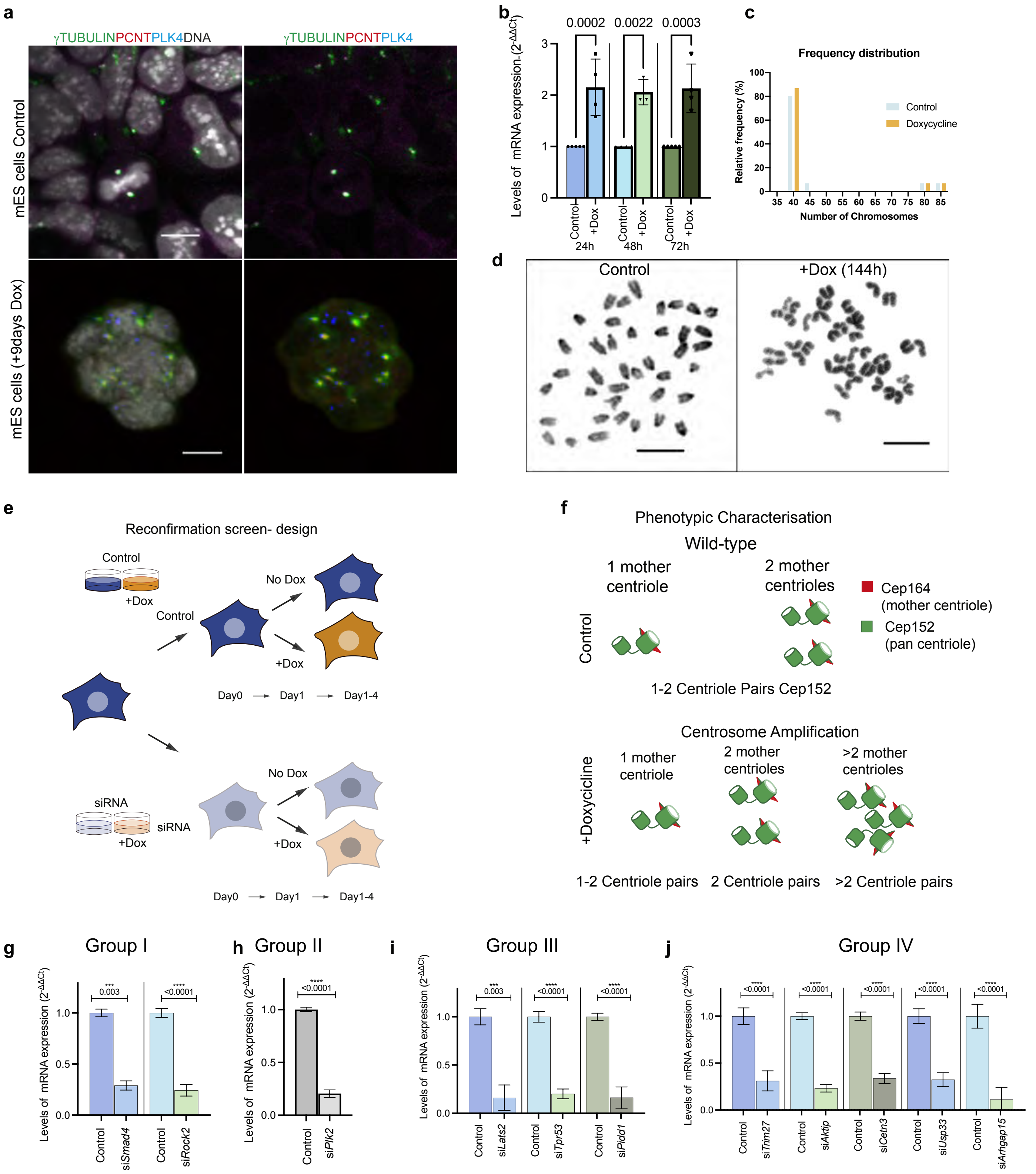

Supplementary Fig. 2. Screening conditions in mouse embryonic stem cells and design of the follow-up screen in mouse embryonic fibroblasts.

(a) PLK4 overexpression in mouse embryonic stem cells (mESCs) induces centrosome amplification. Representative images of control cells and cells treated with doxycycline (+Dox) for 9 days are shown. Cultures were initiated with 1 × 10^6 mESCs; after 9 days of +Dox treatment, only approximately 500 cells remained adherent, of which approximately 90% displayed amplified centrosomes. Immunostaining shows PLK4 (blue), γ-tubulin (green), pericentrin (red), and DNA (DAPI, white). Magnified centrioles boxed in white are shown as merged or PLK4-only images. Scale bars are indicated in merged panels. (**b)** RT–qPCR analysis of selected transcripts in mESCs during PLK4 overexpression. The experiment was repeated independently three times. (**c,d)** Karyotyping of control (n = 10 metaphase spreads) and PLK4-overexpressing (PLK4 OE; n = 10 metaphase spreads) mESCs on day 6. Frequency plots show that chromosome number is not detectably altered by PLK4 overexpression. Representative karyotypes are shown for control and PLK4 OE cells. The experiment was repeated independently three times. (**e)** Schematic of the reconfirmation screen in MEFs. siRNA treatments were applied on day 0, and doxycycline was added on day 1 to induce PLK4 overexpression. Media were refreshed on day 3, and cells were harvested or fixed on day 4 (96 h) for immunofluorescence and quantification. (**f)** Schematic of the centriole quantification strategy. Centrosome amplification was scored using the mother centriole marker CEP164 and the pan-centriolar marker CEP152, and was defined as >2 mother centrioles or >2 CEP152-positive centriole pairs per cell. (**g–j)** Validation of siRNA-mediated knockdown by RT–qPCR. mRNA levels were normalized to *Gapdh* and are shown as relative expression ($2^{-\Delta\Delta Ct}$) versus control cells. Each dot represents an independent biological replicate (n = 3), calculated as the mean of technical replicates; bars indicate mean ± SEM. Statistical significance was determined by one-way ANOVA with Tukey's multiple comparisons test; $P < 0.0001$. (**g)** Group I: si*Smad4*, si*Rock2*. (**h)** Group II: si*Plk2*.(**i)** Group III: si*Lats2*, si*Trp53*, si*Pidd1*. (**j)** Group IV: si*Trim27*, si*Aktip*, si*Cetn3*, si*Usp33* and si*Arhgap15*. Data are provided in Source Data Supplementary Fig. 2.

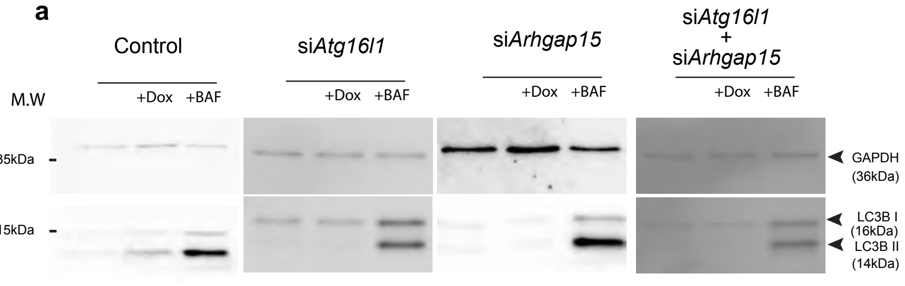

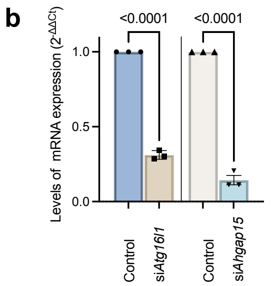

Supplementary Fig. 3 ATG16L1 and ARHGAP15 exert opposing effects on autophagic flux.
(a) Autophagic flux was assessed by monitoring LC3B-II levels after siRNA-mediated depletion of Arhgap15, Atg16l1, or both, compared with control MEFs, in the presence or absence of doxycycline (+Dox). Cells were also treated with bafilomycin A1 (+BAF, 16 h), an inhibitor of autophagosome–lysosome fusion, added 80 h after initiation of siRNA knockdown. Loss of Arhgap15 expression resulted in increased LC3B-II levels relative to control, indicating that ARHGAP15 is required to restrain autophagy. The experiment was repeated independently three times (n = 3). LC3B-II was quantified by densitometry of Western blots and normalized to GAPDH. Western blotting was performed using membranes sequentially incubated with antibodies against LC3B and GAPDH. The corresponding quantification is shown in Fig. 4f. Full, unprocessed Western blot membranes are provided in Source Data 1. **(b)** Validation of Arhgap15 and Atg16l1 siRNA-mediated gene knockdown by RT–qPCR. mRNA expression levels of the indicated genes following siRNA transfection were measured using quantitative RT–PCR and normalized to GAPDH. Data are presented as relative expression (2–ΔΔCt) compared with control cells. Each dot represents an independent biological replicate (n = 3), calculated as the mean of technical replicates. Bars indicate mean ± SEM. Statistical significance was determined using one-way ANOVA followed by Tukey's comparisons test. ****P < 0.0001. Data are provided in Source Data Supplementary Fig. 3.

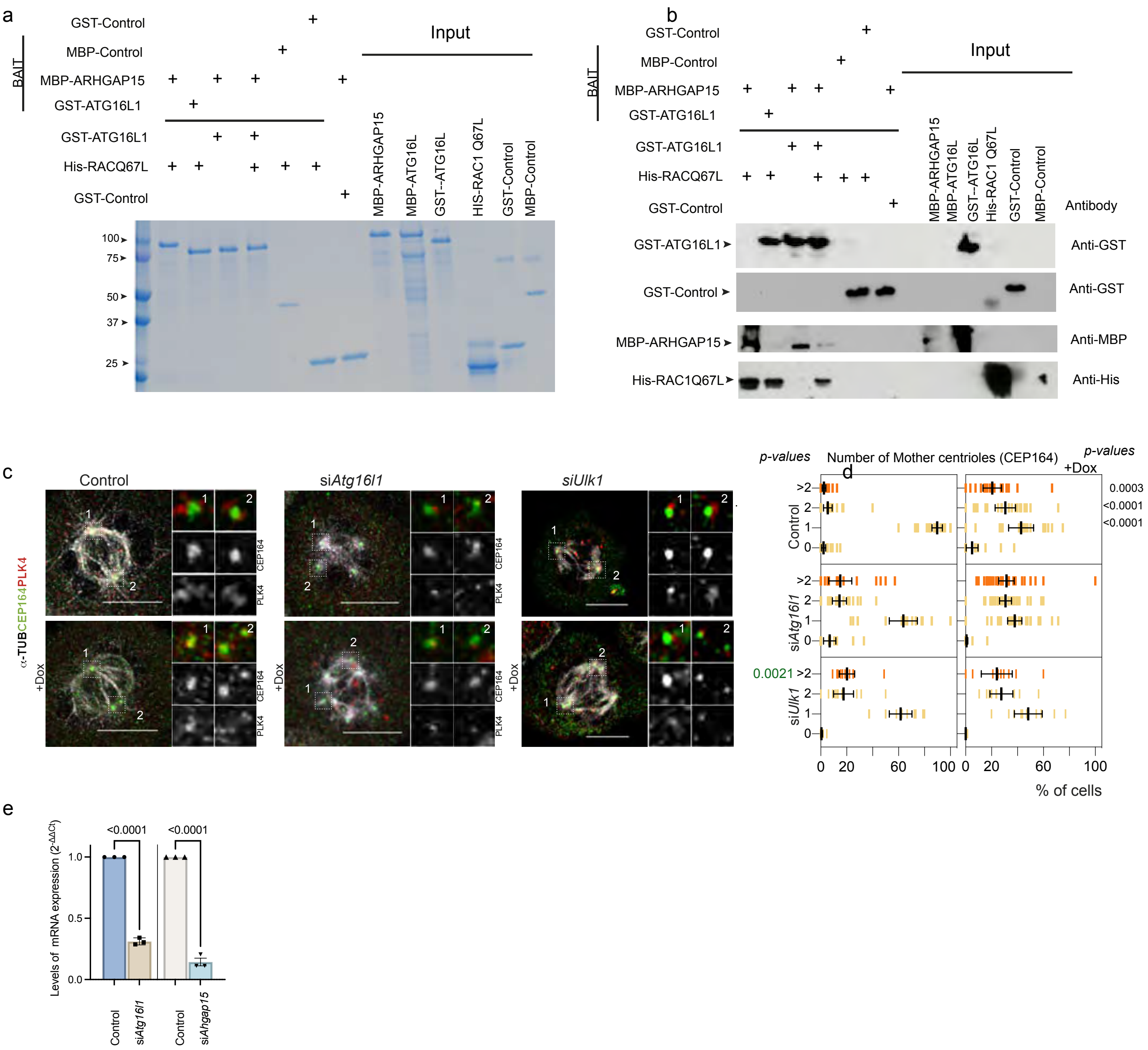

Supplementary Fig. 4. The autophagy pathway controls centrosome number, and interaction with the small GTPase RAC1 acts as a potential modulator.

(**a**) Binding of purified recombinant proteins GST::ATG16L1, His::RAC1-Q67L, and GST as control to MBP::ARHGAP15, MBP::ATG16L1, or MBP as control. Each GST- or His-tagged protein was incubated with MBP-tagged proteins immobilized on maltose-binding beads. Pulldown fractions were analyzed by SDS–PAGE and Coomassie blue staining. **(b)** Western blot analysis of the binding assay shown in (**a**) using anti-MBP and anti-GST antibodies, consistent with binding of ARHGAP15 and ATG16L1 to RAC1 under these assay conditions. Experiments were performed in three independent replicates (n=3). Unprocessed Western blot data are provided in Source Data 2. **(c)** Immunostaining of control Tet^{ON}-*Plk4* MEFs and cells depleted of *Atg16l1* or *Ulk1* by siRNA. Centrosomal components are PLK4 (red), the distal appendage protein CEP164 (green); the spindle is marked with α-tubulin (white). CEP164 labels mother centrioles in each cell. Scale bar, 10 µm. **(d)** Quantification of the percentage of cells with 0, 1, 2, or >2 mother centrioles. Categories are shown as bars (0–2 in yellow; >2 in orange). Error bars indicate mean ± 95% CI. Data are from independent biological replicates: control (n = 27), control +Dox (n = 21), si*Atg16l1* (n = 20), si*Atg16l1* +Dox (n = 35), si*Ulk1* (n = 13) and si*Ulk1* +Dox (n = 11). Statistical significance is indicated by black (control −Dox vs +Dox) and green (control vs corresponding Plk4 overexpression conditions) *p-values*, calculated using the Kruskal–Wallis test with Dunn's multiple comparisons test. Sample sizes and full statistical details are provided in Source Data Supplementary Fig. 4**. (e)** Validation of *Ulk1* and *Atg16l1* siRNA-mediated gene knockdown by RT–qPCR**.** mRNA expression levels of the indicated genes following siRNA transfection were measured using quantitative RT–PCR and normalized to GAPDH. Data are presented as relative expression ($2^{-\Delta\Delta Ct}$) compared with control cells. Each dot represents an independent biological replicate (n = 3), where each replicate is the mean of technical replicates. Bars indicate mean ± SEM. Statistical significance was determined using one-way ANOVA followed by Tukey's comparisons test. ****P < 0.0001.

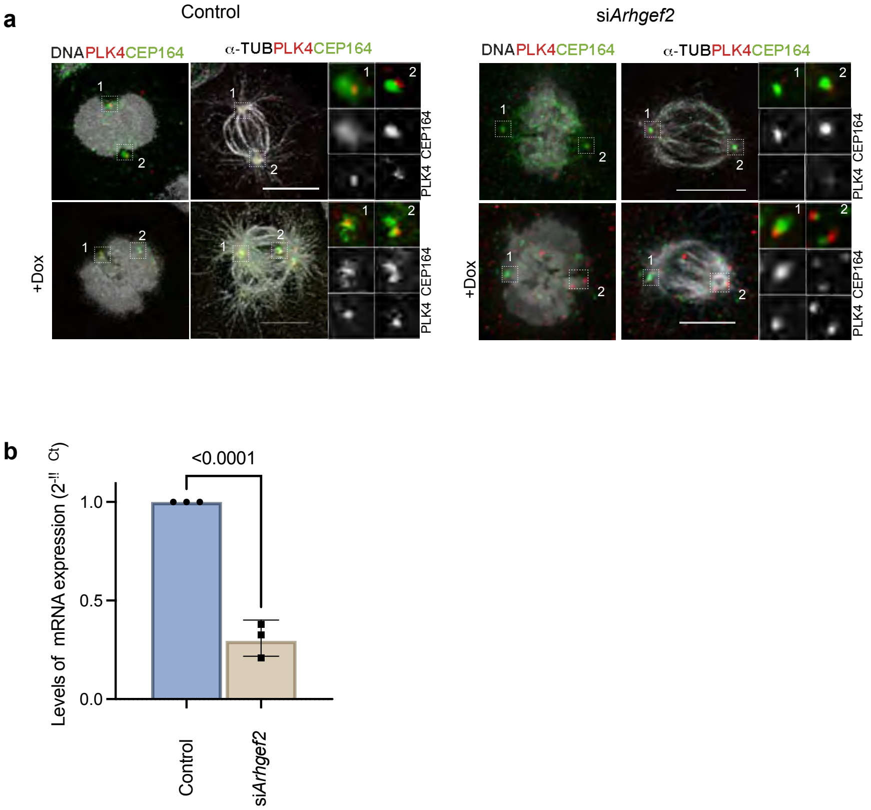

Supplementary Fig. 5. Regulation of centrosome number by the guanine nucleotide exchange factor ARHGEF2. (a) Representative mitotic figures in the presence and absence of doxycycline (Dox)-induced Plk4 overexpression following siRNA-mediated knockdown of Arhgef2. Scale bar, 10 μm. Immunostaining reveals centrosomal PLK4 (green), the distal appendage protein CEP164 (red), and spindle microtubules stained by anti-α-tubulin antibody (white) or DAPI to reveal the DNA. This panel corresponds to Fig. 8b, shown with the additional DNA channel. **(b)** Validation of Arhgef2 siRNA-mediated gene knockdown by RT–qPCR. mRNA expression levels of the indicated genes following siRNA transfection were measured using quantitative RT–PCR and normalized to GAPDH. Data are presented as relative expression (2–ΔΔCt) compared with control cells. Each dot represents an independent biological replicate (n = 3), where each replicate is the mean of technical replicates. Bars indicate mean ± SEM. Statistical significance was determined using one-way ANOVA followed by Tukey's comparisons test. ****P< 0.0001.

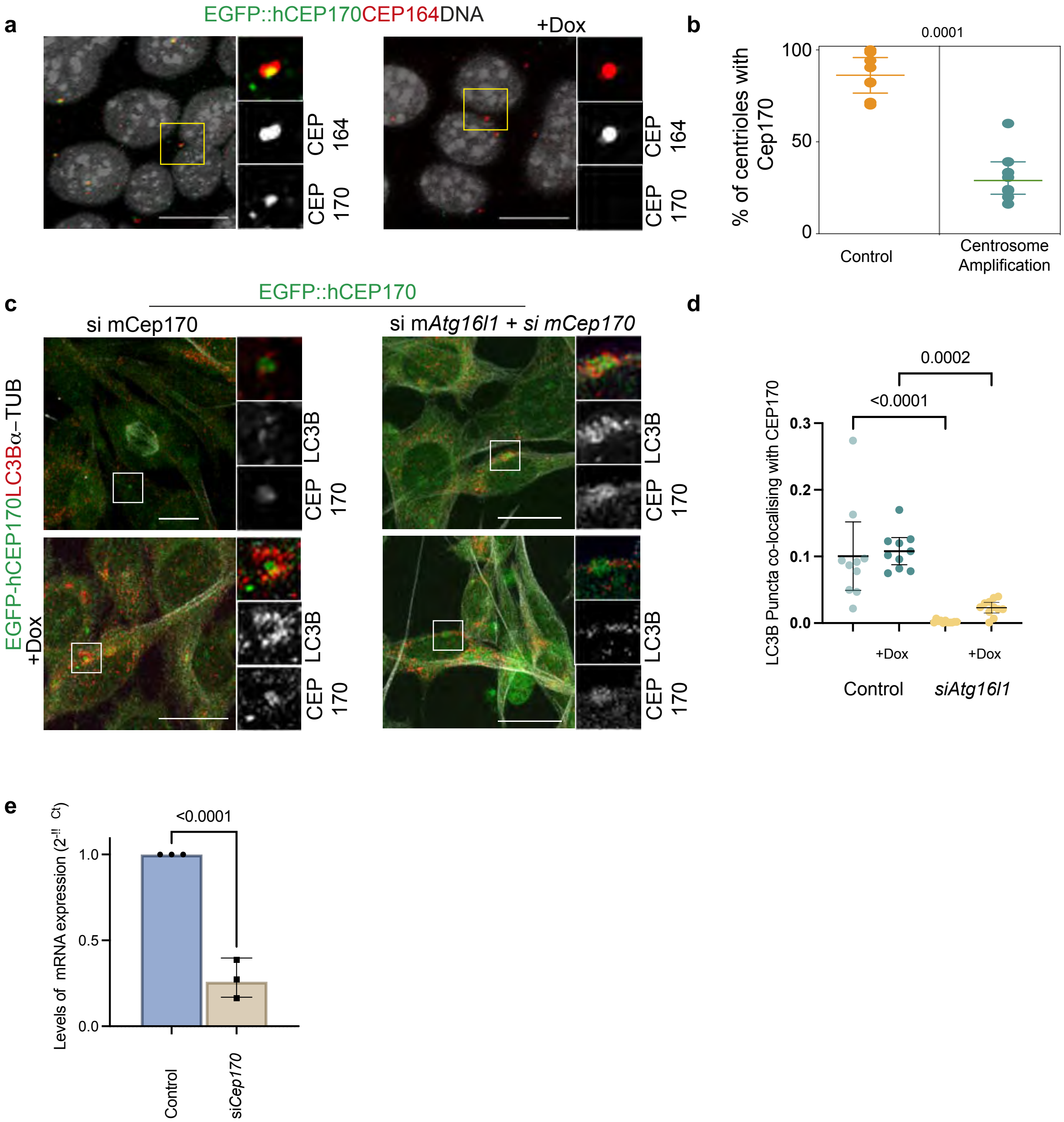

Supplementary Fig. 6 CEP170 localizes to centrioles and autophagosomes.

(a) Representative images of control *Tet^{ON}–Plk4* MEFs and *Tet^{ON}–Plk4* MEFs expressing EGFP–human CEP170 following siRNA-mediated knockdown of endogenous *Cep170*. Cells are shown in the absence or presence of doxycycline (+Dox) to induce PLK4 expression and were stained for CEP170 (green), CEP164 (red) and DNA (white). Insets show enlarged views of centriolar staining. (**b)** Quantification of the percentage of centrioles showing CEP170 colocalization with CEP164. Error bars indicate mean ± 95% CI. Statistical significance was assessed using a two-tailed exact Mann–Whitney U test; n = 9 independent biological replicates per group, U = 0, P < 0.0001. (**c)** Representative *Tet^{ON}–Plk4* MEFs expressing EGFP–human CEP170 following siRNA-mediated knockdown of endogenous *Cep170*, alone or together with *Atg16l1* depletion, in the absence or presence of doxycycline-induced PLK4 expression. Cells were stained for CEP170 (green), LC3B (red), and α-tubulin or DNA (white), as indicated. Scale bar, 10 μm. (**d)** Quantification of the percentage of LC3B-positive puncta colocalizing with CEP170. Error bars indicate mean ± 95% CI. Data were collected from independent biological replicates: control, n = 10; siAtg16l1, n = 11. Individual P values are shown for statistically significant comparisons. Sample sizes and additional P values are provided in Source Data Supplementary Fig. 6. **(e)** Validation of *Cep170* siRNA-mediated knockdown by RT–qPCR. mRNA levels were normalized to *Gapdh* and are shown as relative expression values calculated using the $2^{-\Delta\Delta Ct}$ method versus control cells. Each dot represents an independent biological replicate (n = 3), calculated as the mean of technical replicates; bars indicate mean ± SEM. Statistical significance was determined using one-way ANOVA followed by Tukey's multiple comparisons test; ****P < 0.0001.

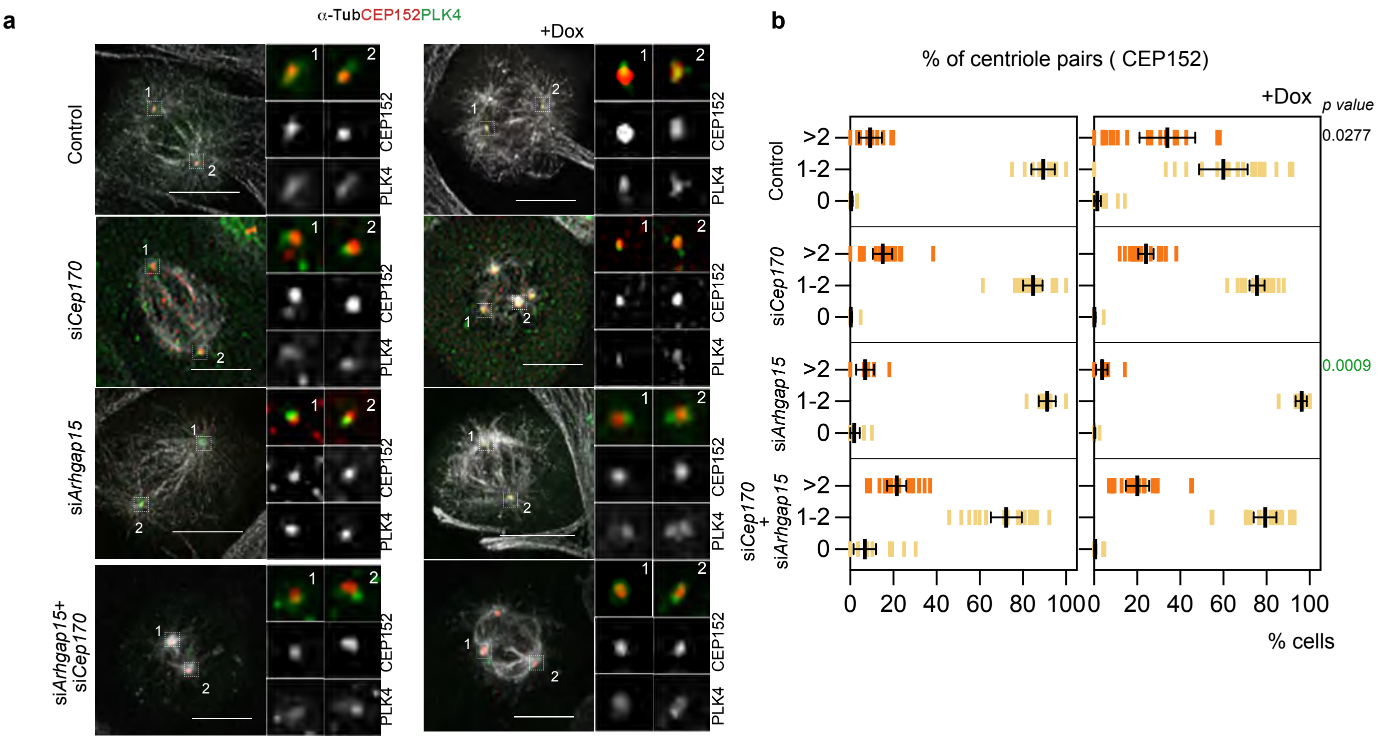

Supplementary Fig. 7 CEP170 and autophagy regulators. (a) Representative mitotic cells shown in the absence or presence of doxycycline (+Dox)-induced PLK4 expression in TetON–Plk4 MEFs following siRNA-mediated knockdown of Cep170 or Arhgap15, either singly or in the indicated combinations. Cells were stained for PLK4 (green), CEP152 (red) and α-tubulin (white). Magnified insets from two spindle poles are shown. Scale bar, 10 µm. **(b)** Quantification of the percentage of cells with 0, 1–2 or >2 centriole pairs per cell. Categories 0–2 are shown in yellow and >2 in orange. Error bars indicate mean ± 95% CI. Data are from independent biological replicates: control (n = 11), control +Dox (n = 12), siCep170 (n = 12), siCep170 +Dox (n = 12), siArhgap15 (n = 12), siArhgap15 +Dox (n = 12), and siArhgap15 + siCep170 +Dox (n = 12). Statistical analysis was performed using the Kruskal–Wallis H test followed by Dunn's multiple comparisons test with Bonferroni correction. Statistical significance is indicated by black P values for control −Dox versus control +Dox, and green P values for control versus corresponding PLK4-overexpression conditions. Raw data and additional statistical details are provided in Source Data Supplementary Fig. 7.

Source Data 1

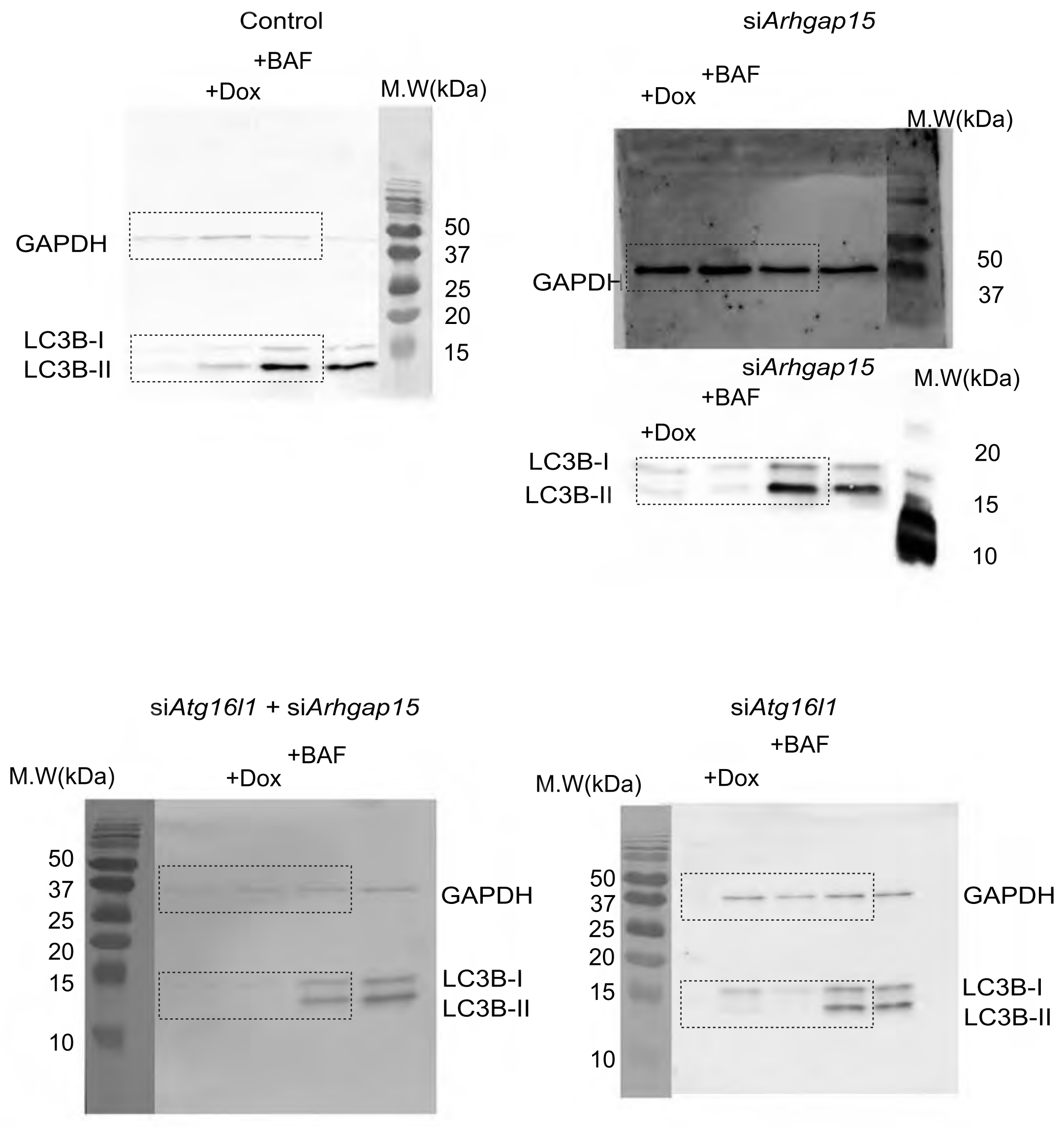

Source Data 1. Unprocessed Western blots for Supplementary Fig. 3a

Full, unprocessed Western blot membranes are shown for experiments monitoring LC3B-II levels following siRNA-mediated depletion of *Arhgap15*, *Atg16l1*, or both, compared with control MEFs, in the presence or absence of doxycycline (+Dox). Where indicated, cells were treated with bafilomycin A1 (+BAF; 16 h), an inhibitor of autophagosome–lysosome fusion, added 80 h after initiation of siRNA knockdown. Western blotting was performed using antibodies against LC3B and GAPDH. For this experiment, the membrane was cut after transfer to allow separate incubation and detection of LC3B and GAPDH. The experiment was repeated independently three times.

Source Data 2

a

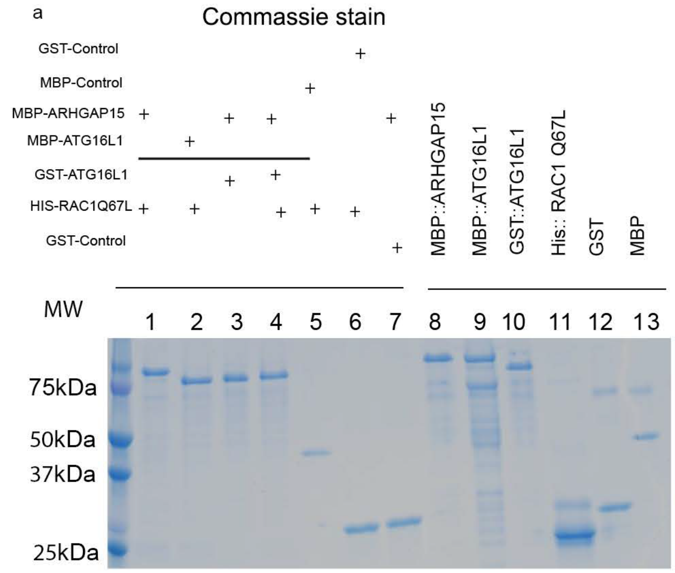

b

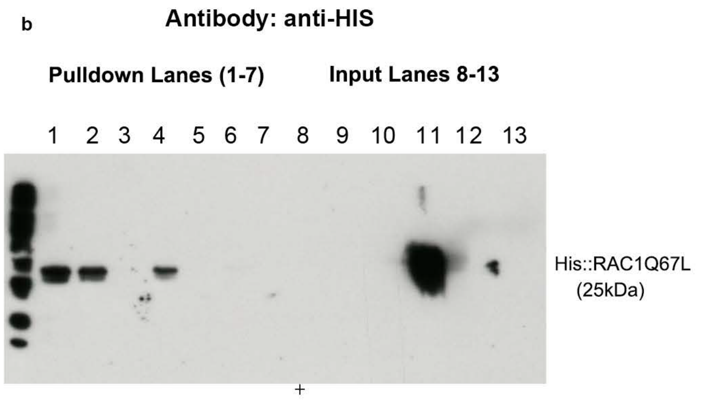

Pulldown Lanes (1-7)

1. MBP::ARHGAP15+ His::RAC1 Q67L
2. GST:: ATG16L1 + His::RAC1 Q67L
3. GST::ATG16L1::MBP::ARHGAP15
4. GST:: ATG16L1 + MBP:: ARHGAP15 + His:: RAC1 Q67L
5. MBP Control + His:: RAC1 Q67L
6. GST Control + His:: RAC1 Q67L
7. GST Control + MBP::ARHGAP15

Input Lanes 8-13

8.MBP::ARHGAP15
9.MBP::ATG16L1
10. GST::ATG16L1
11. His::RAC1 Q67L
12. GST
13. MBP

c

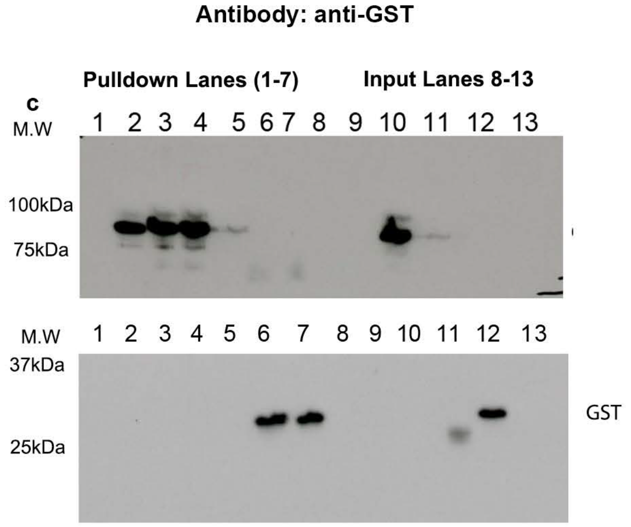

d

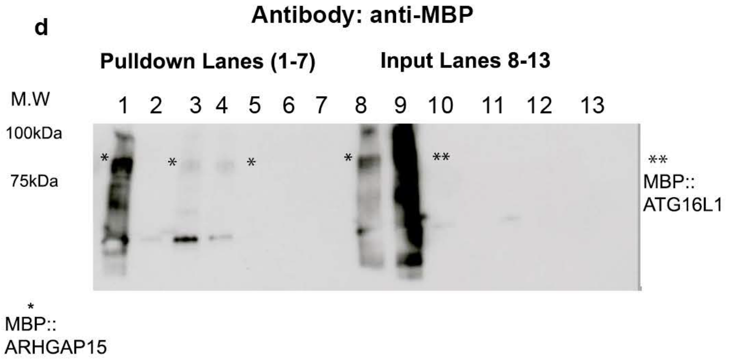

Source Data 2. Unprocessed Western blots for Supplementary Fig. 4

Pulldown assays were performed with MBP–ARHGAP15, GST–ATG16L1, and His–RAC1-Q67L, alone or in combination with control proteins. Bound proteins were analysed by SDS–PAGE followed by Coomassie staining (a), and by immunoblotting with anti-His (b), anti-GST (c), and anti-MBP (d) antibodies. Lanes 1–7 correspond to pulldown reactions, whereas lanes 8–13 represent input controls: lane 8, MBP–ARHGAP15; lane 9, MBP–ATG16L1; lane 10, GST–ATG16L1; lane 11, His–RAC1-Q67L; lane 12, GST; lane 13, MBP. Molecular weight markers are indicated in kDa. One asterisk indicates the MBP–ARHGAP15 signal; two asterisks indicate the MBP–ATG16L1 signal. Boxed regions highlight the areas presented in the corresponding Supplementary Fig. 4 panels. For this experiment, membranes were cut after transfer to allow separate incubation and detection with the indicated antibodies. The experiment was repeated independently three times.
