# Supplementary material for: Sensing centrosome amplification: the interface between centriole duplication and autophagy
Source: Nat Commun. 2026 Jun 21;17:7815. doi: 10.1038/s41467-026-74702-9 (PMC13439419; doi:10.1038/s41467-026-74702-9)
Supplement: Supplementary file 4 [file 41467_2026_74702_MOESM2_ESM.pdf]

Description of Additional Supplementary Files

Supplementary Data 1. Genome-wide CRISPR/Cas9 screen results and pathway enrichment analysis.

Supplementary Data 2. Validation of candidate genes affecting proliferation and mother centriole number following PLK4-induced centrosome amplification. Source data and statistical analyses Fig. 1.

Supplementary Data 3. ARHGAP15 regulates centrosome number through ATG16L1. Source data and statistical analyses Fig. 2.

Supplementary Data 4. Mass spectrometry and BioGRID interaction datasets.

Supplementary Data 5. ARHGAP15 is a negative regulator of autophagy, and RAC1 regulates centrosome amplification. This workbook contains the source data and statistical analyses corresponding to Fig. 3.

Supplementary Data 6. ARHGAP15 is a negative regulator of autophagy.
This workbook contains the source data and statistical analyses corresponding to Fig. 4.

Supplementary Data 7. Inhibition of autophagy affects centrosome number. This workbook contains the source data and statistical analyses corresponding to Fig. 5.

Supplementary Data 8. ATG16L1 binds RAC1 through the coiled-coil domain, and ULK1 kinase activates ATG16L1 in centrosome autophagy. This workbook contains source data and statistical analyses corresponding to Fig. 6.

Supplementary Data 9. ULK1 kinase activates ATG16L1 in centrosome autophagy. This workbook contains source data and statistical analyses corresponding to Fig. 7.

Supplementary Data 10. Regulation of centrosome number by the GTP exchange factor ARHGEF2. This workbook contains source data and statistical analyses corresponding to Fig. 8.

Supplementary Data 11. The relationship between CEP170 and regulators of autophagy. This workbook contains source data and statistical analyses corresponding to Fig. 9.

Supplementary Data 12. Reagents, cell lines, plasmids, siRNAs and primers used in this study.
